# Does Impedance Matter When Recording Spikes With Polytrodes?

**DOI:** 10.3389/fnins.2018.00715

**Published:** 2018-10-08

**Authors:** Joana P. Neto, Pedro Baião, Gonçalo Lopes, João Frazão, Joana Nogueira, Elvira Fortunato, Pedro Barquinha, Adam R. Kampff

**Affiliations:** ^1^CENIMAT/I3N and CEMOP/Uninova, Departamento de Ciência dos Materiais, Faculdade de Ciências e Tecnologia, Universidade Nova de Lisboa, Caparica, Portugal; ^2^Champalimaud Neuroscience Programme, Champalimaud Centre for the Unknown, Lisbon, Portugal; ^3^Sainsbury Wellcome Centre, University College London, London, United Kingdom

**Keywords:** microelectrodes, impedance, spikes, noise, coating

## Abstract

Extracellular microelectrodes have been widely used to measure brain activity, yet there are still basic questions about the requirements for a good extracellular microelectrode. One common source of confusion is how much an electrode’s impedance affects the amplitude of extracellular spikes and background noise. Here we quantify the effect of an electrode’s impedance on data quality in extracellular recordings, which is crucial for both the detection of spikes and their assignment to the correct neurons. This study employs commercial polytrodes containing 32 electrodes (177 μm^2^) arranged in a dense array. This allowed us to directly compare, side-by-side, the same extracellular signals measured by modified low impedance (∼100 kΩ) microelectrodes with unmodified high impedance (∼1 MΩ) microelectrodes. We begin with an evaluation of existing protocols to lower the impedance of the electrodes. The poly (3,4-ethylenedioxythiophene)-polystyrene sulfonate (PEDOT-PSS) electrodeposition protocol is a simple, stable, and reliable method for decreasing the impedance of a microelectrode up to 10-fold. We next record *in vivo* using polytrodes that are modified in a ‘chess board’ pattern, such that the signal of one neuron is detected by multiple coated and non-coated electrodes. The performance of the coated and non-coated electrodes is then compared on measures of background noise and amplitude of the detected action potentials. If the proper recording system is used, then the impedance of a microelectrode within the range of standard polytrodes (∼0.1 to 2 MΩ) does not greatly affect data quality and spike sorting. This study should encourage neuroscientists to stop worrying about one more unknown.

## Introduction

Throughout the electrophysiology literature, an electrode’s impedance magnitude measured at 1 kHz in a saline solution is regularly used as a proxy for its ability to detect the activity of individual neurons (Nam, 2012; Alivisatos et al., 2013; Won et al., 2018). Actually, the impedance is a measure of the ability of the solution-electrode interface circuit to resist the flow of charge across the interface’s phases (i.e., from the ionic to electronic conductor).

How much does an electrode’s impedance affect its signal-to-noise ratio (SNR) and thus ability to detect spikes? Several studies suggest that electrode impedance has a major impact on SNR (Ludwig et al., 2006; Keefer et al., 2008; Ferguson et al., 2009; Ansaldo et al., 2011; Baranauskas et al., 2011; Du et al., 2011; Ludwig et al., 2011; Scott et al., 2012; Chung et al., 2015; Kozai et al., 2015; Zhao et al., 2016). However, there is also literature showing that electrode impedance does not greatly affect SNR (Cui et al., 2001; Suner et al., 2005; Desai et al., 2010). Commercially available silicon probes, also called polytrodes, have relatively high impedance electrodes due to their low surface area and small diameters (<50 μm), which are suitable for recording single unit activity. Materials such as Au, Pt, and Ir are often used as the electrode material in polytrodes, and lowering the electrode impedance prior to recording is a ‘standard’ step in various laboratories (Desai et al., 2010). How does one lower the impedance of commercial polytrodes? Electrodeposition is a simple and reproducible technique, yet has great flexibility to produce a variety of coatings (Ferguson et al., 2009). For more details about electrodeposition techniques see (Santos et al., 2015). By electroplating Au or Pt, the surface roughness increases and the electrode impedance decreases (Cui and Martin, 2003; Ferguson et al., 2009; Desai et al., 2010; Márton et al., 2014). Over the last decade, conductive polymers, particularly poly(3,4-ethylenedioxythiophene) (PEDOT), have been electrodeposited onto electrodes due to their chemical stability and mechanical integrity when implanted in the brain (Ludwig et al., 2006; Ludwig et al., 2011; Kozai et al., 2016). Moreover, when compared to metals, these polymers are typically softer materials offering a more intimate contact between the electrode surface and brain tissue (Green et al., 2008). Prior to the electrodeposition, a dopant is added to the synthesis solution to improve conductivity; the most common dopant molecule is polystyrene sulfonate (PSS) (Guimard et al., 2007; Aregueta-Robles et al., 2014).

Our goal was simply to answer the question: ‘should I reduce the impedance of my polytrode electrodes’? Despite the prevalence of this question in the field, a definitive answer is still lacking. It is important to quantify the impact of an electrode’s impedance on the background noise and amplitude of extracellular spikes to determine if the effort to reduce impedance by electrodeposition is necessary.

## Materials and Methods

### Polytrodes

All experiments were performed with a commercially available 32-channel probe (A1x32-Poly3-5mm-25s-177-CM32, NeuroNexus), with 177 μm^2^ area electrodes (iridium) and an inter-site pitch of 22–25 μm (see **Supplementary Figure S1**).

### Coatings

NanoZ hardware and software (Neuralynx) was used to perform gold and PEDOT-PSS electrodepositions. Moreover, both coatings were galvanostatically deposited in a two electrode cell configuration consisting of the probe microelectrodes individually selected as the working electrode and a platinum wire as the reference electrode. The reference wire was placed around the deposition cup while the probe was maintained at a fixed and equal distance to all points of the reference wire. By selecting ‘Manual Control’ from the NanoZ software it is possible to select individual electrodes.

For gold coatings, a commercial non-cyanide gold solution was obtained from Neuralynx. The deposition solution for PEDOT-PSS consisted of 0.01 M of EDOT (Sigma-Aldrich, 97%, Mw = 142.18) and 0.1 M of PSS (Sigma-Aldrich, Mw = 1000000) dissolved in deionized water. The optimal deposition parameters were -30 nA during 120 s for gold and +30 nA during 5 s for PEDOT-PSS (Baião, 2014). Before and after the deposition, electrode impedance magnitude at 1 kHz was measured in sterile phosphate buffer saline solution (PBS, 1 mM, pH 7.4) with NanoZ. Post-deposition assessment of coating morphology was performed by scanning electron microscopy (SEM-FIB, Zeiss Auriga).

### Electrochemical Characterization

The electrochemical behavior of microelectrodes was studied in PBS (1 mM, pH 7.4) by electrochemical impedance spectroscopy (EIS). For the electrochemical characterization, a potentiostat (Reference 600, Gamry Instruments) was used with a three electrode cell configuration where probe microelectrodes were connected individually as the working electrode, a platinum wire served as the counter electrode, and an Ag-AgCl (3 M KCl, Gamry Instruments) as the reference electrode. The impedance was measured in a frequency range from 1 Hz to 100 kHz by applying a sinusoidal signal with an amplitude of 10 mV.

### *In vivo* Acute Recordings

Before and after each acute recording, the impedance magnitude of each electrode was measured using a protocol implemented by the RHD2000 series chip (Intan Technologies), with the probe microelectrodes placed in a dish with sterile PBS (1 mM, pH 7.4) and a reference electrode, Ag-AgCl wire (Science Products GmbH, E-255). Following each surgery, cleaning was performed by immersing the probe in a trypsin solution (Trypsin-EDTA (0.25%), phenol red, TermoFisher Scientific) for 30–120 min and rinsing with distilled water (Neto et al., 2016).

For the surgeries under ketamine, Long Evans rats (400–700 g, both sexes) were anesthetized with a mixture of ketamine (60 mg/kg) and medetomidine (0.5 mg/kg), and placed in a stereotaxic frame. At the initial stage of each ketamine surgery, atropine was given to suppress mucus secretion (0.1 mg/kg, atropine methyl nitrate, Sigma-Aldrich). For the surgeries under urethane, rats (400–700 g, both sexes) of the Lister Hooded strain were anesthetized with urethane (1.6 g/kg) and placed in a stereotaxic frame. At the initial stage of each urethane surgery, the animal was injected with atropine (0.05 mg/kg), temgesic (20 μg/kg), and rimadyl (5 mg/kg). Ketamine, medetomidine and urethane were administered by intraperitoneal injection, while temgesic and rimadyl were administered by subcutaneous injection. Atropine was administered by intramuscular injection.

Anesthetized rodents then underwent a surgical procedure to remove the skin and expose the skull above the targeted brain region. Small craniotomies (2 mm medial-lateral and 2 mm anterior-posterior) were performed above the target area. The acute recordings were conducted in different brain regions and at different depths (for more details see **Supplementary Figure S2** and **Supplementary Table S1**). The reference electrode Ag-AgCl wire (Science Products GmbH, E-255) was inserted at the posterior part of the skin incision. Equipment for monitoring body temperature as well as a live video system for performing probe insertion were integrated into the setup. For the extracellular recordings we used the Open Ephys [27] acquisition board along with the RHD2000 series interface chip that amplifies and digitally multiplexes the signal from the 32 extracellular electrodes (Intan Technologies). Extracellular signals in a frequency band of 0.1–7,500 Hz were sampled at 20 or 30 kHz with 16-bit resolution and were saved in a raw binary format for subsequent offline analysis using the Bonsai framework (Lopes et al., 2015; Bonsai, 2017).

Animal experiments under urethane were approved by the local ethical review committee and conducted in accordance with Home Office personal and project (I67952617; 70/8116) licenses under the UK Animals (Scientific Procedures) 1986 Act. Animal experiments under ketamine were approved by the Champalimaud Foundation Bioethics Committee and the Portuguese National Authority for Animal Health, Direcção-Geral de Alimentação e Veterinária.

### Analysis

For the noise and signal (spikes amplitude) characterization, a third order Butterworth filter with a band-pass of 250–9,500 or 14,250 Hz (95% of the Nyquist frequency) was used in the forward-backward mode in all datasets.

The magnitude of the background noise was estimated from the median absolute signal, assuming a normal noise distribution, σ_Median_ = median(|signal(t)|/0.6745) avoiding contamination by spike waveforms (Quiroga et al., 2004). Alternatively, the noise was defined as the standard deviation (σ_RMS_) of the signal (Scott et al., 2012).

We ran Kilosort (Pachitariu et al., 2016) for spike sorting on all the datasets with the maximum number of templates set to 128 (four times the number of electrodes on our probe). This algorithm iteratively generates templates and then uses these templates to detect and classify the individual spikes. Each spike is assigned to the template that matches it best. Afterward, we used Phy (Rossant et al., 2016) to check the automatically generated clusters. Phy is a graphical user interface for refining the results of spike sorting. After the manual sorting we used functions to assess cluster quality^1^. The “well isolated” units considered for the signal analysis have simultaneously low interspike interval (ISI) violations and contamination rates, and high isolation distances values. Neurons with more than 200 spikes were considered for further analyses. The average spike waveform of all spikes from each unit on a given recording site was plotted and the respective peak-to-peak (P2P) amplitude was computed (see **Supplementary Figure S3**).

Some results are presented as mean ± standard deviation. Impedance magnitude, background noise and spikes amplitude from pristine and PEDOT coated electrodes were compared for significance using the Mann-Whitney test. Moreover, to evaluate coating stability the impedance magnitude from electrodes after the electrodeposition and after acute surgeries were also compared for significance using the Mann-Whitney test.

## Results

### Microelectrode Coating

**FIGURE 1:**
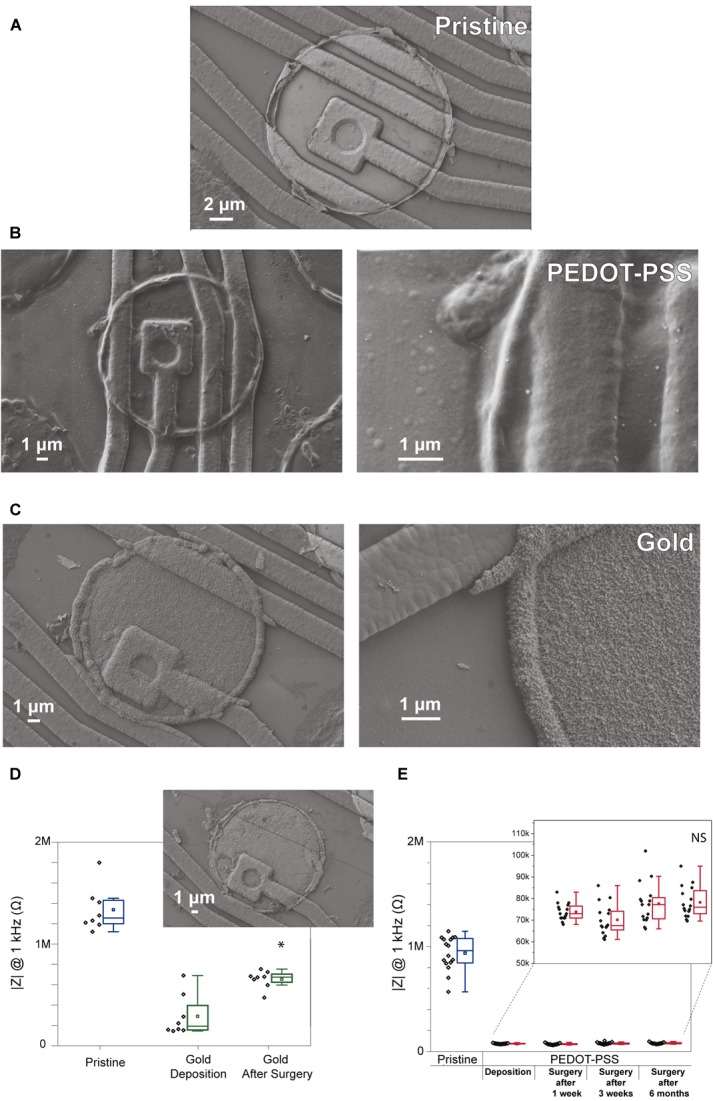
Comparison of gold coated electrodes and PEDOT-PSS coated electrodes. SEM images showing the surface morphology of electrodes from a commercial polytrode in their original state, and after the coatings. **(A)** Pristine electrode, **(B)** PEDOT-PSS coated electrode, and **(C)** gold coated electrode. **(D)** Impedance stability of gold coating for 8 electrodes from one polytrode before, after the deposition and after one acute surgery. SEM image insert of the gold coating from one electrode after the surgery. **(E)** Impedance stability of PEDOT-PSS coating for 16 electrodes from one polytrode before, after the deposition, and after acute surgeries performed 1 week, 3 weeks, and 6 months after the deposition. Black points denote impedance magnitude measured at 1 kHz in saline solution for individual electrodes, and boxplots show the distribution of these values. In the boxplots, line: median, square: mean, box: 1st quartile–3rd quartile, and whiskers: 1.5× interquartile range above and below the box. ^∗^*p* < 0.001 when compared with electrodes after deposition. ‘NS’ not significant (*p* > 0.05) when compared with electrodes after deposition.

This study begins with an evaluation of existing electrodeposition protocols to reduce impedance of microelectrode. **Figures 1A–C** reveals the morphological differences between a pristine iridium electrode, PEDOT-PSS coated electrode, and gold coated electrode (**Figures 1A–C**, respectively). Pristine electrodes typically display a smooth surface with almost no irregularities (although some might occur due to the microfabrication process). Gold coating creates a rough structure on the electrode, which leads to an increase in surface area, one of the key factors in lowering the impedance magnitude at 1 kHz in saline solution (Rivnay et al., 2017). However, in **Figure 1D** we observe that even though the impedance after coating is lower when compared to the pristine counter-part, these values tend to increase following an acute surgery. This may reflect the poor adhesion of the gold coating to the iridium electrodes (**Figure 1D**). The gold instability and delamination was also observed in some previous studies (Scott et al., 2012). In the case of PEDOT-PSS coated electrodes (**Figures 1B,E**), they have a ‘fuzzy’ coating and the impedance values after the deposition remained stable for a long period of time, allowing for repeated acute surgeries (1 week, 3 weeks, and 6 months after the deposition). Therefore, taking into account the impedance value of PEDOT-PSS coated electrodes (values under 100 kΩ) and its stability, this coating was considered ideal for reducing the polytrode microelectrodes impedance.

**FIGURE 2:**
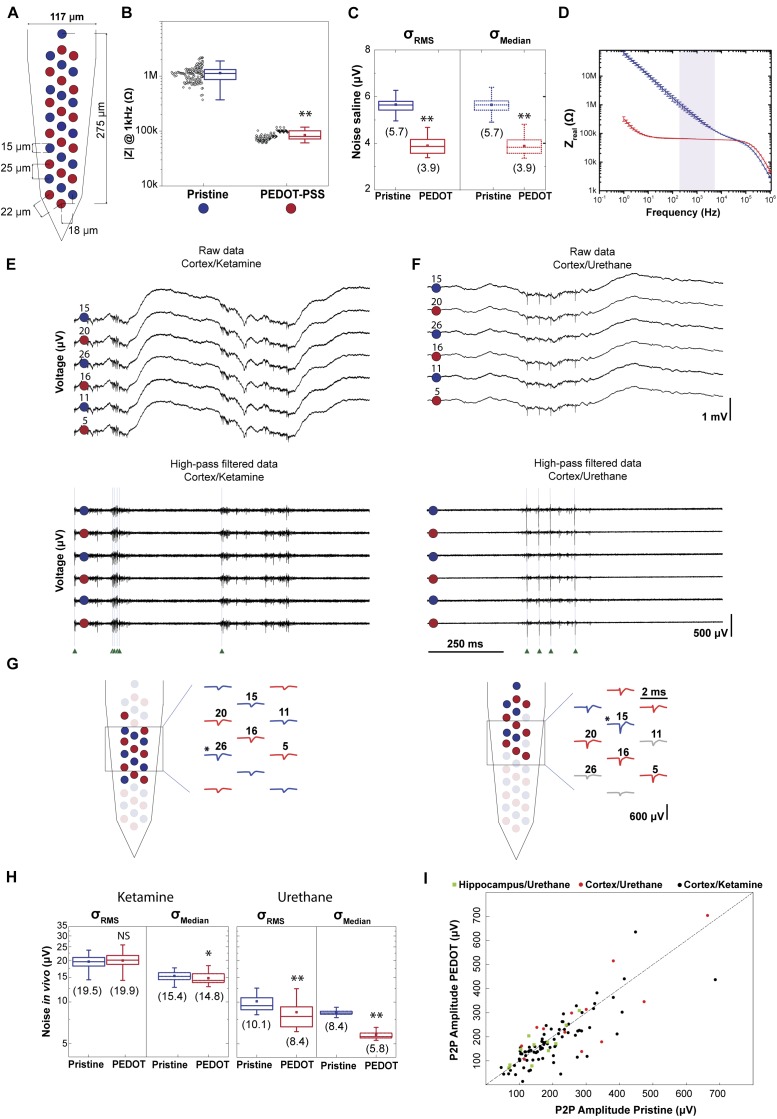
Impact of impedance on data quality. **(A)** Schematic of a polytrode where electrodes were modified in a ‘chess board’ pattern. Red circles represent PEDOT-PSS coated electrodes and blue circles represent pristine electrodes. **(B)** Reliability of PEDOT-PSS electrodeposition protocol. Impedance magnitude measured at 1 kHz in saline solution for 3 polytrodes (*n*_pristine_ = 48 and *n*_PEDOT_ = 48). Black points denote individual measurement for each electrode (3 measurements for each electrode). **(C)** Noise σ_RMS_ and σ_Median_ of recordings performed in saline solution (*n*_pristine_ = 48 and *n*_PEDOT_ = 48). Values in parentheses show mean value. **(D)** Impedance spectroscopy of PEDOT-PSS coated (*n* = 3) and pristine (*n* = 3) electrodes shows a significant decrease in the impedance real value. The light purple shaded area corresponds to the frequency range in which the thermal noise was computed. **(E)** 1 s-long raw data traces from 6 electrodes, 3 coated and 3 non-coated, from the recording ‘*amplifier2014_11_25T23_00_08.bin’*. This recording was carried out in cortex under ketamine anesthesia. Top: signals correspond to the 0.1–7.5 kHz frequency band. Bottom: high-pass filtered traces to highlight spontaneous spiking activity. Green arrows indicate the time of spikes identified for a putative neuron. **(F)** The same representation as in **(E)** for the recording ‘*amplifier2017_02_02T15_49_35.bin’.* This recording was carried out in cortex under urethane anesthesia. **(G)** Representative putative neurons from each of the recordings shown above. Left panel corresponds to the cortex/ketamine recording and right panel to the cortex/urethane recording. Schematic of two polytrodes with red and blue colored waveforms and circles denoting the electrodes with the highest peak-to-peak amplitudes from each unit, respectively. The asterisks indicates the electrode with the maximum amplitude P2P. **(H)** σ_RMS_ and σ_Median_ of 9 acute recordings performed in rat cortex, 6 of which under ketamine, and 3 under urethane (*n*_PEDOT_ket_ = 96, *n*_Pristine_ket_ = 96, *n*_PEDOT_ure_ = 48 and *n*_Pristine_ure_ = 48). Values in parentheses show mean value. **(I)** The maximum P2P amplitude average for coated electrodes and for non-coated is plotted for 103 sorted neurons. In the boxplots, line: median, square: mean, box: 1st quartile–3rd quartile, and whiskers: 1.5× interquartile range above and below the box. ^∗^*p* < 0.001 when compared with pristine electrodes. ^∗∗^*p* < 0.0001 when compared with pristine electrodes. ‘NS’ not significant (*p* > 0.05) when compared with pristine electrodes.

**Figure 2A** illustrates the polytrode microelectrode array design employed to assess the impact of impedance on data quality. Electrodes from three polytrodes were coated in a ‘chess board’ pattern such that the signal of one neuron is detected by both coated and non-coated electrodes. In each polytrode 16 electrodes were coated with PEDOT-PSS. In **Figure 2B** the impedance at 1 kHz for three polytrodes was 1.1 ± 0.4 MΩ for pristine electrodes (*n* = 48) and 0.084 ± 0.015 MΩ for PEDOT coated electrodes (*n* = 48). As can be seen from the figure, the PEDOT-PSS electrodeposition protocol is reliable across probes and electrodes (3 polytrodes, *n*_pristine_ = 48 and *n*_PEDOT_ = 48).

### Noise Characterization: In Saline

First, the performance of PEDOT-PSS coated electrodes was compared to pristine electrodes in terms of noise, both in saline solution and during *in vivo* recordings. The contribution of all non-biological noise sources was measured by recording signals from microelectrodes immersed in a saline solution. The non-biological sources include the electronic noise due to the amplifier, thermal noise, and noise associated with the double layer interface (Hassibi et al., 2004; Baranauskas et al., 2011). At room temperature, the actual noise measured in saline solution for pristine and PEDOT coated microelectrodes is shown in **Figure 2C**. The σ_Median_ noise value in saline, for pristine electrodes was 5.7 ± 0.4 μV and for PEDOT coated electrodes was 3.9 ± 0.4 μV, which represents a reduction of about 30%. Additionally, the σ_RMS_ and σ_Median_ values are similar in saline solution.

The thermal noise depends on the real part of the measured impedance. The thermal noise computed in the 200–8,000 Hz frequency band for pristine (*n* = 3) microelectrodes was 5.0 μV and for PEDOT coated (*n* = 3) microelectrodes was 2.8 μV (**Figure 2D** and for a detailed description see **Supplementary Material**). Additionally, the electronic noise due to the amplifier in our system, measured by shorting the headstage inputs, was 2.0 ± 0.1 μV. We can predict the non-biological noise value as the square root of the sum of the squared thermal noise (5.0 μV and 2.8 μV for pristine and coated microelectrodes, respectively) with the squared electronic noise (∼2.0 μV). The predicted values for the noise in saline (5.4 μV in non-coated and 3.4 μV in coated) were similar to the measured noise values (5.6 μV in non-coated and 3.9 μV in coated).

### Noise Characterization: *In vivo*

We next recorded *in vivo* using the same polytrodes with the ‘chess board’ pattern described in **Figure 2A**. These recordings were conducted in different brain regions and at different depths (**Supplementary Figure S2** and **Supplementary Table S1**). Also, ketamine and urethane anesthesia was used to compare noise and signal magnitude recorded during different brain states (Hildebrandt et al., 2017; **Figures 2E,F**). Under ketamine, the cortex switches between periods of higher neuronal activity and periods of much lower activity (up and down states) (Ruiz-Mejias et al., 2011). Under urethane anesthesia, the activity is similar to natural brain activity during sleep (Clement et al., 2008; Pagliardini et al., 2013).

**Figures 2E,F** highlight the variability of noise *in vivo* (i.e., biological noise magnitude is highly variable due to variations in background neural firing rate). Note that, in general, the magnitude of noise under ketamine is higher compared to urethane, due to the increase in this background activity. Moreover, the values of noise vary with the method used to compute the noise magnitude. Higher values for the noise *in vivo* were found when taking into consideration σ_RMS_ values, probably due to the contribution of spikes. The σ_RMS_ value is based on the standard deviation of the signal, which increases with the firing rate (Quiroga et al., 2004). Therefore, the σ_Median_ noise values were used to compare the noise between experiments, and within an experiment. Under urethane, the σ_Median_ values from coated electrodes are smaller compared to the non-coated electrodes. On average, the σ_Median_ value was reduced from 8.4 ± 0.4 μV in non-coated to 5.8 ± 0.5 μV in PEDOT coated microelectrodes, a 30% reduction. Under ketamine the σ_Median_ noise was 15.4 ± 1.2 μV in non-coated and 14.8 ± 1.3 μV in PEDOT coated microelectrodes.

The noise values found for *in vivo* recordings are highly variable (**Figure 2H**) and the noise reduction observed in saline is likely preserved *in vivo*, yet masked by the much larger variation in background spiking activity. Does the difference in noise observed between coated and non-coated electrodes matter for detecting spikes? Usually, the negative voltage deflection of a well isolated unit exceeds 40–70 μV. Therefore, the benefits resulting from the ∼2 μV noise reduction achieved by coating electrodes would be irrelevant for detecting spikes.

### Signal Characterization: Amplitude of Action Potentials

Although not resulting in a major reduction of noise at relevant frequencies, it is still possible that coating electrodes might increase the amplitude of each spike (see **Supplementary Figure S5** for more details about attenuation of signal). **Figure 2G** shows two examples of putative neurons where each waveform corresponds to the average of all the spikes from the respective neuron on a given recording electrode. Additionally, red and blue colored waveforms and circles denote electrodes where the peak-to-peak average amplitude is larger than half of the maximum peak-to-peak average amplitude of the isolated neuron. Therefore, they represent the electrodes with the highest peak-to-peak amplitude from each neuron.

For each of the 103 putative neurons sorted from 11 recordings, the largest average peak-to-peak amplitudes from the pristine and PEDOT electrode groups were plotted (**Figure 2I**). Therefore, for each neuron, two values are plotted in **Figure 2I**, corresponding to the pristine and PEDOT electrode with the largest average peak-to-peak amplitude. If the largest peak-to-peak amplitude spikes are detected by the PEDOT coated electrodes (low impedance electrodes), then the scatter points would fall above the unity line. However, if the largest peak-to-peak amplitude spikes are detected in the pristine electrodes (high impedance electrodes), the scatter points would fall below the line. Our results show that the probability of recording the largest peak-to-peak amplitude spikes is similar for coated and non-coated electrodes and the peak-to-peak amplitude values from the pristine and PEDOT electrode groups are similar (see **Supplementary Figure S6**). Therefore, there is no obvious relationship between impedance and the peak-to-peak amplitude of sorted neurons in this impedance range.

## Discussion

### Side-by-Side Impedance Comparison

The ability to record from closely spaced electrodes permitted accurate comparisons between electrodes with two very different impedance values. The PEDOT-PSS electrodeposition protocol made it possible to decrease impedance up to 10-fold on average, from 1.1 ± 0.4 MΩ to 0.084 ± 0.015 MΩ. We divided our noise analysis into non-biological noise (noise measured in saline solution) and biological noise, where the level of noise was assessed during acute recordings within the cortex of anesthetized rats. As expected with the impedance reduction, we found a reduction in noise magnitude in saline after coating, since the thermal noise is proportional to the square root of the real part of the impedance (Baranauskas et al., 2011). The reduction in impedance resulted in ∼30% decrease in the non-biological noise. Nevertheless, when using electrodes *in vivo*, this reduction in the thermal noise is largely overwhelmed by the much larger biological noise and would not improve the detection of spikes with commercial polytrodes. Moreover, we found no significant effect of impedance on spike peak-to-peak amplitude and detection probability on both coated and non-coated electrodes. In summary, the impedance values found at 1 kHz in commercial silicon polytrode microelectrodes don’t seem to affect data quality during spike recording. Moreover, the entire dataset used to quantify the effect of an electrode’s impedance on data quality is available online^2^ and summarized in **Supplementary Table S1**.

### But Why Different Views About the Impact of Impedance?

Electrophysiological studies report different views of the impedance impact on data quality. Many studies show that decreasing the impedance improves the signal-to-noise ratio because of thermal noise reduction, while others find that impedance reduction did not affect greatly the signal-to-noise ratio.

In studies where researchers use tetrodes and single microwires, lowering the impedance is beneficial because a low-impedance electrode minimizes signal loss through shunt pathways (usually capacitive coupling to ground). Shunt capacitance can be significant in long, thinly insulated electrode wires (Robinson, 1968). Thus, for tetrodes and microwires, lowering impedance will result in a larger signal for both local field potentials and spikes (Ferguson et al., 2009). However, with silicon polytrodes, shunt capacitance is much smaller and does not appear to cause signal attenuation for typical values of polytrode electrodes impedance (Obien et al., 2015).

However, if polytrodes, particularly those with higher impedance values (>2 MΩ), are used with a differential amplifier that has a (relatively) low input impedance, then a voltage-divider is formed between the electrode and amplifier. The amplifier from Intan Technologies has an input impedance of 13 MΩ, and with electrode impedances of 1 MΩ and 100 kΩ, the signal loss is around 7% and 1%, respectively, which may be negligible, but for an electrode with 3 MΩ impedance, this signal loss is around 20%. For more details about the voltage divider occurrence, see **Supplementary Figure S5**.

Do we need to coat our polytrode electrodes? No, assuming we have a good amplifier and low shunt capacitance. But we propose that microelectrode coatings, in chronic applications, may do more than just reduce the impedance. Some coatings may help to promote cell health at the electrode surface and minimize the immune response of surrounding brain tissue. Strong neural attachment to implanted electrodes is desirable as it increases interface stability and improves electrical transfer across the tissue-electrode interface (Green et al., 2008; Nam, 2012; Jorfi et al., 2015; Kook et al., 2016). We thus propose that we stop worrying about impedance magnitude (as long as it stays well below the input impedance of the amplifier) and start focusing on bio-compatible materials (Bellamkonda et al., 2012; Chen and Allen, 2012; Jorfi et al., 2015).

## Data Availability

The datasets generated for this study can be found in the http://www.kampff-lab.org/polytrode-impedance/.

## Author Contributions

JPN, GL, JF, and AK conceived and designed the research. JPN, JF, JN, and PBa performed the experiments. JPN and AK analyzed the data. JPN, JF, PBar, EF, and AK interpreted the results of experiments. JN and AK prepared the figures. JN and AK drafted the manuscript. All authors read and approved the submitted version of the manuscript.

## Conflict of Interest Statement

The authors declare that the research was conducted in the absence of any commercial or financial relationships that could be construed as a potential conflict of interest.

## Footnotes

1 https://github.com/cortex-lab/sortingQuality

2 http://www.kampff-lab.org/polytrode-impedance/
