# Supplementary material for: Does Impedance Matter When Recording Spikes With Polytrodes?
Source: Front Neurosci. 2018 Oct 8;12:715. doi: 10.3389/fnins.2018.00715 (PMC6188074; doi:10.3389/fnins.2018.00715)
Supplement: Supplementary file 1 [file Presentation_1.pdf]

Supplementary Material

Does impedance matter when recording spikes with polytrodes?

Joana P Neto*, Pedro Baião, Gonçalo Lopes, João Frazão, Joana Nogueira, Elvira Fortunato, Pedro Barquinha, Adam R Kampff

*** Correspondence:** Joana Neto: j.neto@ucl.ac.uk

Commercial polytrode

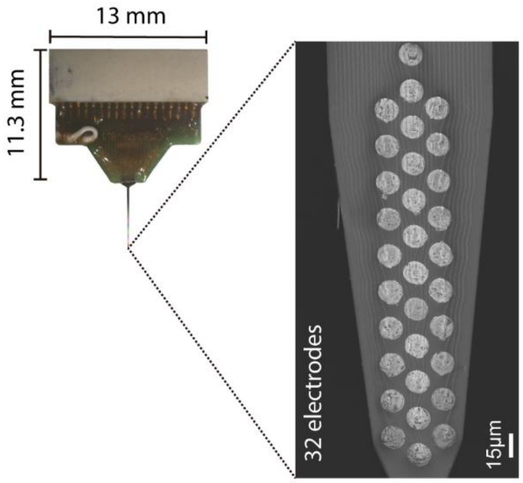

Supplementary Figure 1. Commercial polytrode containing 32 iridium electrodes (15 µm diameter and 22-25 µm inter-site pitch) arranged in a dense array. For noise and spikes amplitude characterization, sixteen electrodes were coated with PEDOT-PSS in a 'chess board' pattern. This allow us to directly compare, side-by-side, the same extracellular signals measured by PEDOT-coated microelectrodes (low impedance) with non-coated microelectrodes (high impedance).

Acute recordings

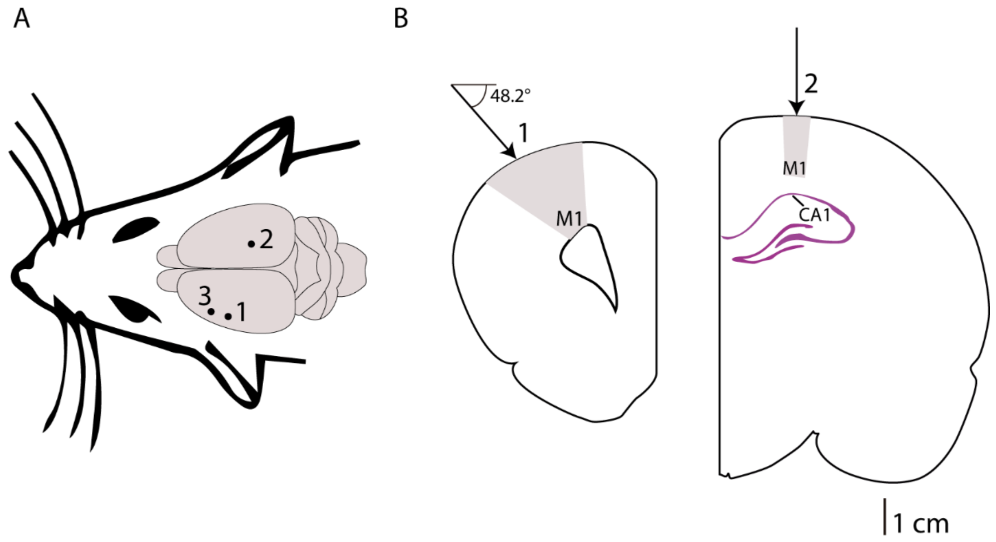

Supplementary Figure 2. Acute recordings performed with polytrodes containing 32 electrodes that are modified in a ‘chess board’ pattern. **(A)** Schematic of a rat brain signaling the probe insertion positions (black dots); **(B)** In the coronal slices the arrows represent the direction and position of probe at the insertion point. Under insertion points 1, 2 and 3 we recorded from primary motor cortex (M1). Under the insertion 2 we also recorded from hippocampus CA1. The estimated recording position in the brain was based on the depth (distance from the point of insertion to the bottom electrode in the array) and physiological signatures. The probe direction was similar in insertion 1 and 3.

Dataset summary

Filename	Depth (µm)	Brain region /Anaesthesia	Number of putative neurons	Probe ID	Animal ID	AP (mm)
amplifier2015-05-05T17_20_09.bin	—	Short-circuit	—			
amplifier2015-05-11T11_59_54.bin	—	Saline solution	—	P1		
amplifier2017-02-02T10_30_29.bin	—	Saline solution	—	P2		
amplifier2016-11-01T19_59_18.bin	—	Saline solution	—	P3		
amplifier2017-02-02T14_38_11.bin	715	M1 / urethane	1	P2	A	3.1
amplifier2017-02-02T15_03_44.bin	915	M1 / urethane	6	P2	A	3.1
amplifier2017-02-02T15_49_35.bin	1245	M1 / urethane	5	P2	A	3.1
amplifier2017-02-02T16_57_16.bin	2149	Hippocampus CA1/ urethane	6	P2	A	3.1
amplifier2017-02-02T17_18_46.bin	2243	Hippocampus CA1/ urethane	5	P2	A	3.1
amplifier2014-11-13T14_59_40.bin	400	M1 / ketamine	9	P1	B	3.4
amplifier2014-11-13T15_35_31.bin	700	M1 / ketamine	17	P1	B	3.4

amplifier2014-11-13T18_05_50.bin	1100	M1 / ketamine	24	P1	B	3.4
amplifier2014-11-25T20_32_48.bin	1300	M1 / ketamine	11	P1	C	3.2
amplifier2014-11-25T21_27_13.bin	1400	M1 / ketamine	7	P1	C	3.2
amplifier2014-11-25T23_00_08.bin	1300	M1 / ketamine	12	P1	C	3.2

Supplementary Table 1. For each recording (i.e., filename), we described the distance (depth) from the brain surface to the bottom electrode in the array, the estimated recorded brain region (M1 or CA1), the anaesthesia (ketamine or urethane), the number of putative neurons sorted for each recording, the probe and animal identification (ID), and the anterior-posterior (AP) distance relative to bregma for each recording. Acute recordings from anesthetized rats are approximately 10 to 15 minutes long. Additionally, the recordings used to calculate the noise in saline and the electronic noise due to the amplifier contribution are also presented. This dataset is available online (http://www.kampff-lab.org/polytrode-impedance/). M1 denotes primary motor cortex and CA1 an hippocampal area.

Putative neurons

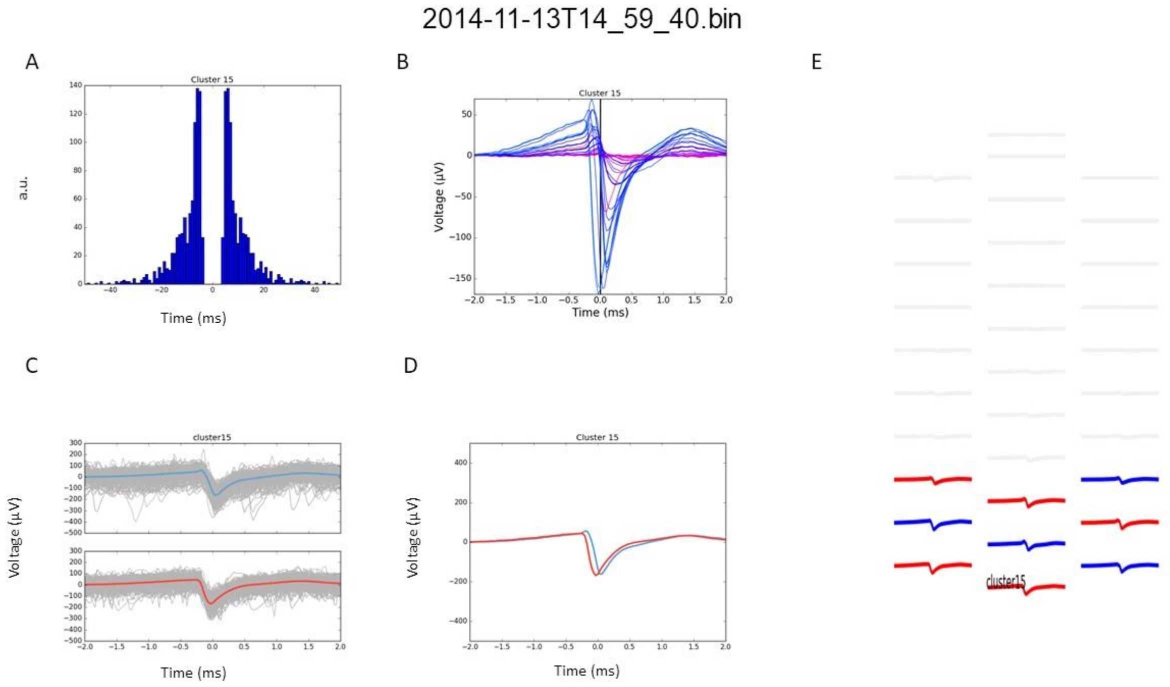

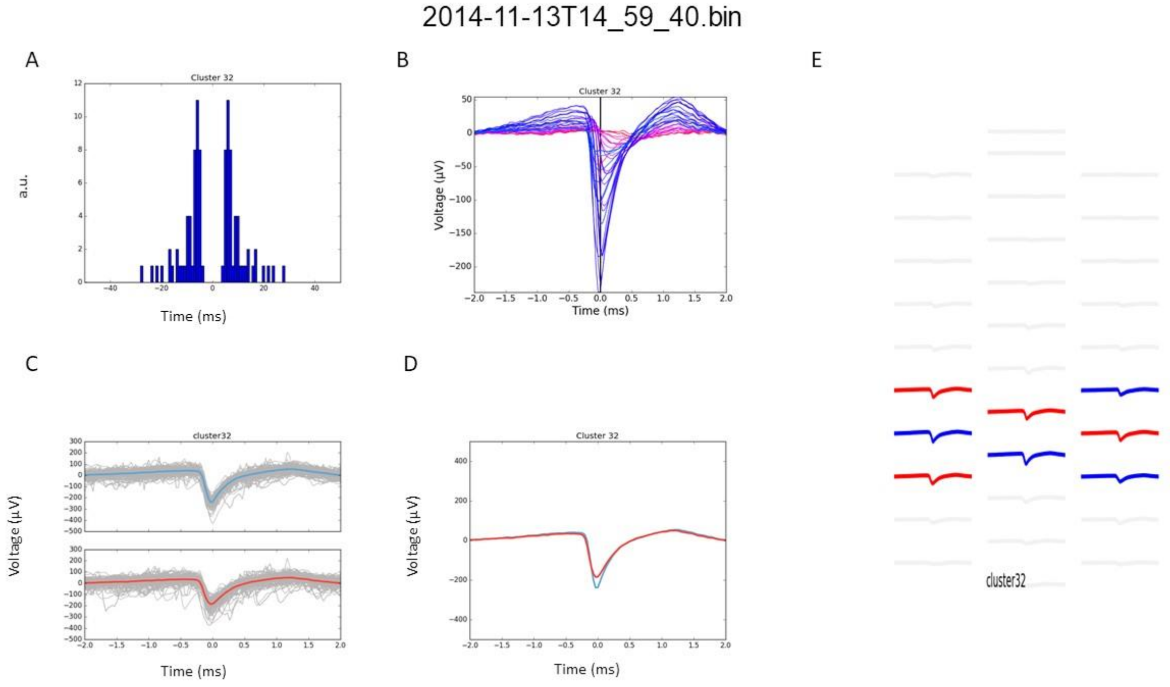

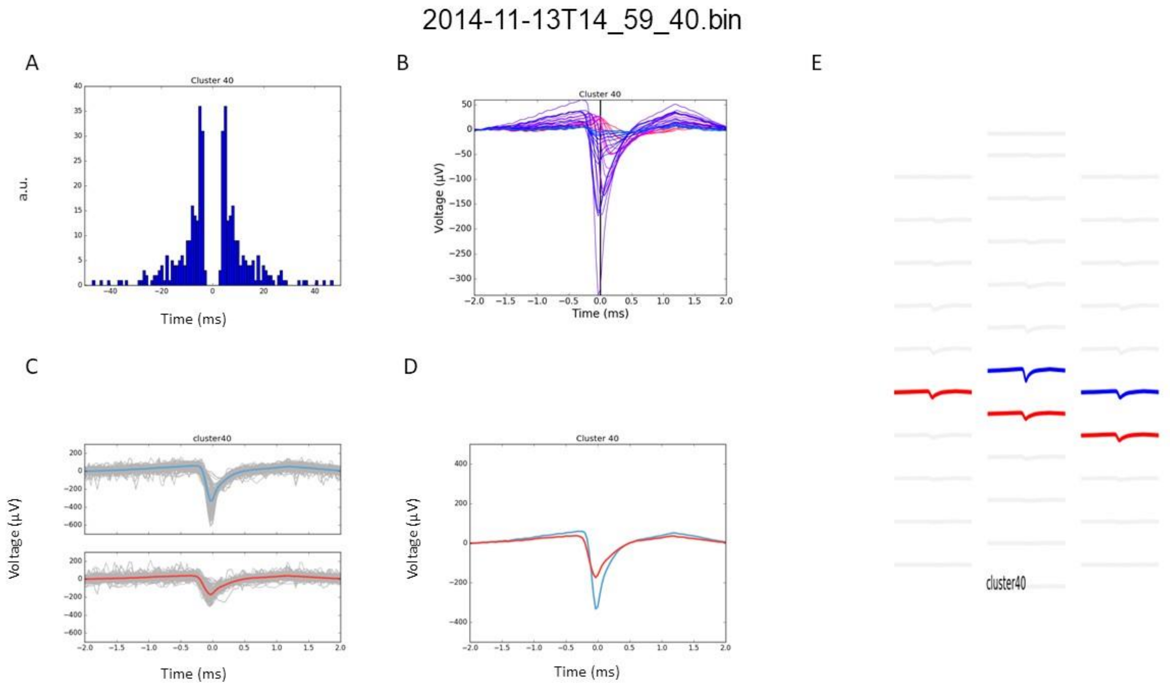
2014-11-13T14_59_40.bin
A
B
E
Cluster 40
a.u.
Time (ms)
Voltage (µV)
C
D
cluster40
Voltage (µV)
Time (ms)
Cluster 40
Voltage (µV)
Time (ms)
cluster40

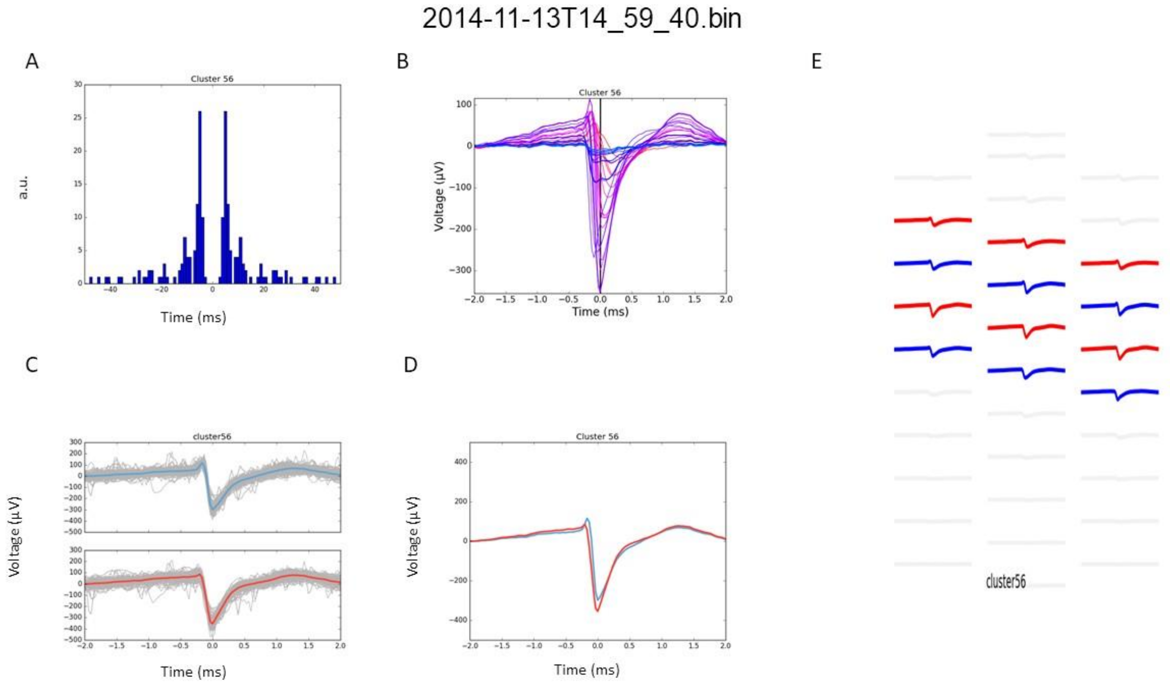
2014-11-13T14_59_40.bin
A
B
E
Cluster 56
a.u.
Time (ms)
Voltage (µV)
C
D
cluster56
Voltage (µV)
Time (ms)
Cluster 56
Voltage (µV)
Time (ms)
cluster56

2014-11-13T14_59_40.bin

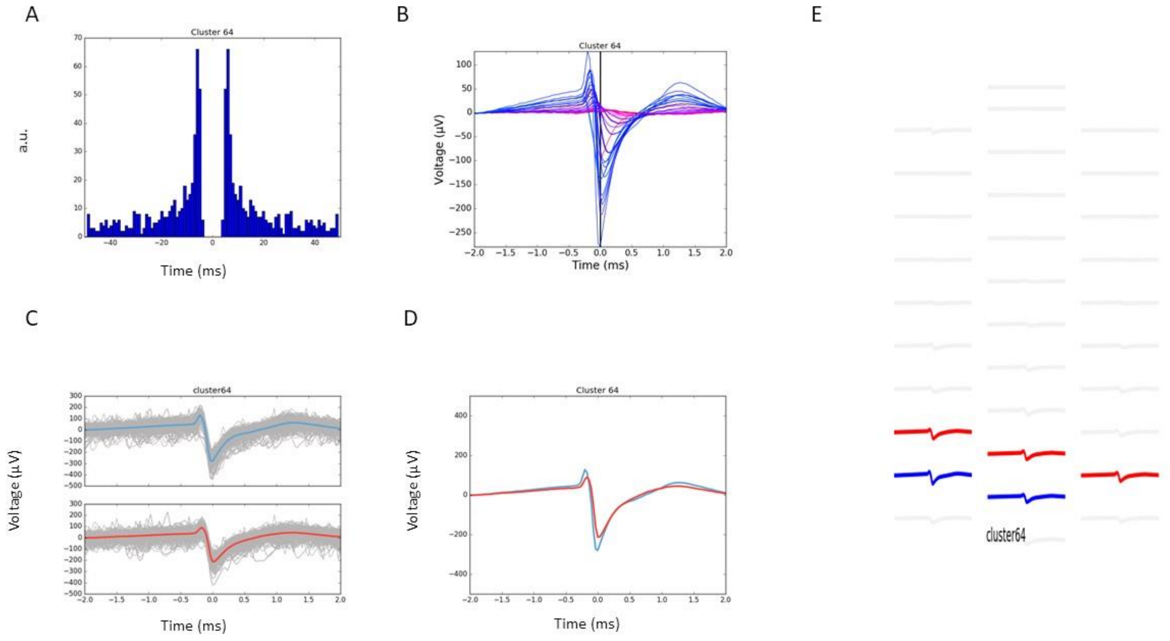

2014-11-13T14_59_40.bin

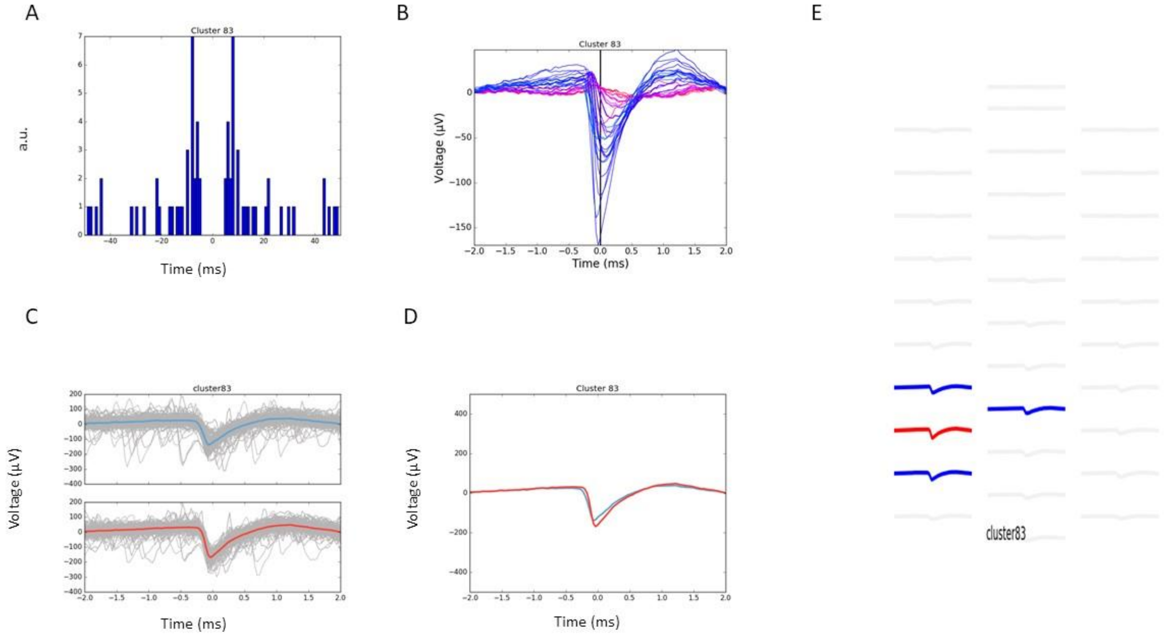

2014-11-13T14_59_40.bin

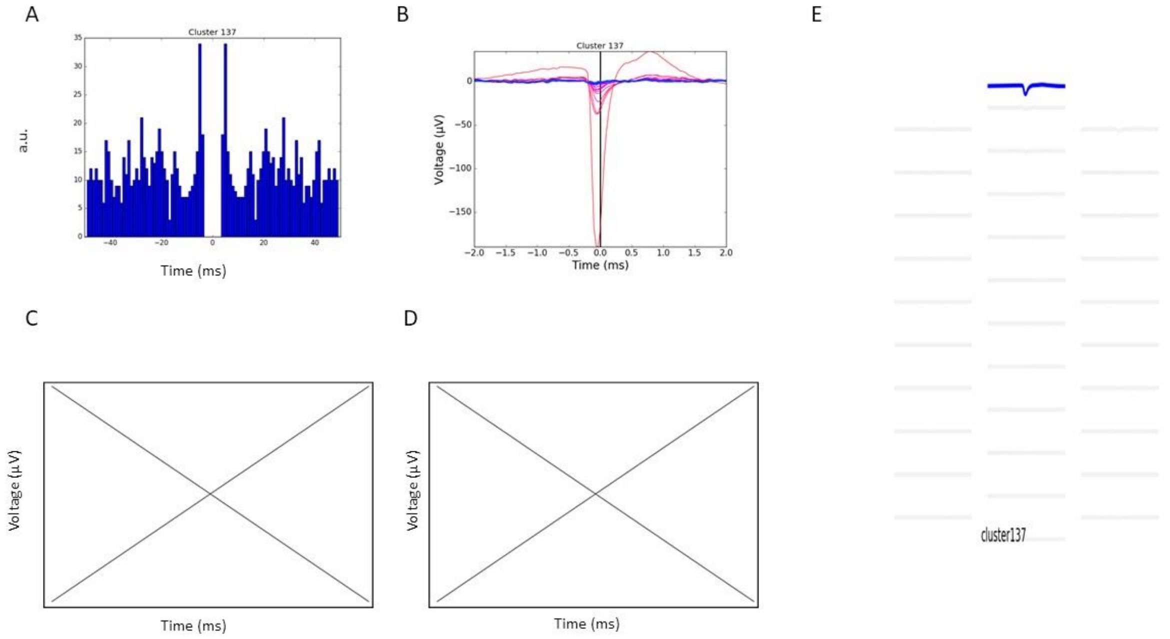

2014-11-13T14_59_40.bin

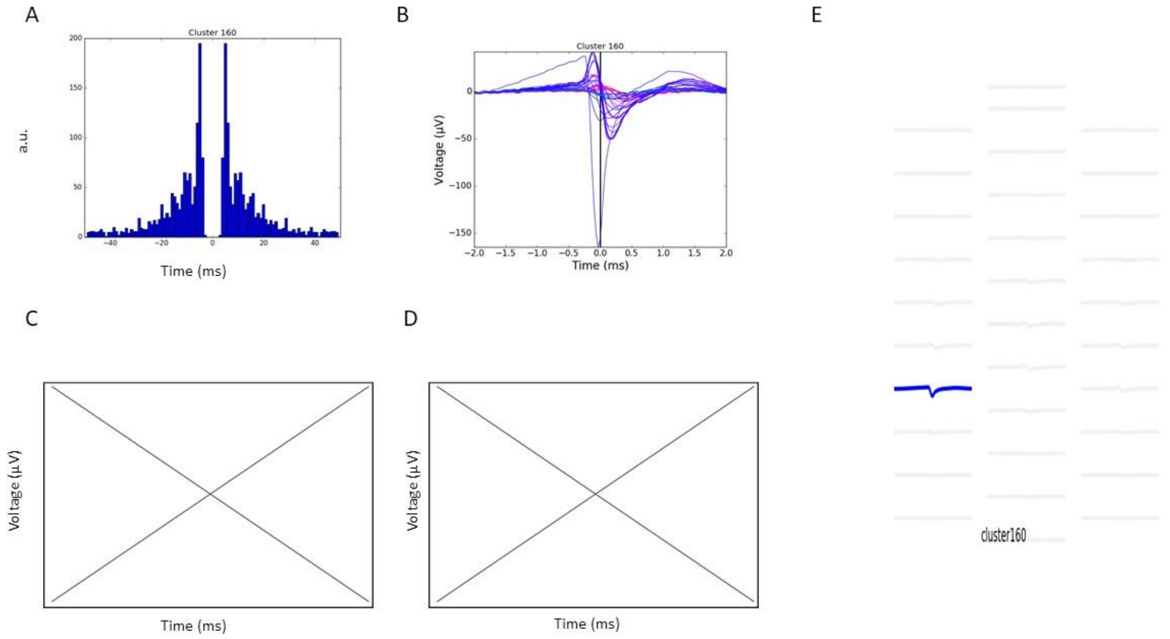

2014-11-13T14_59_40.bin

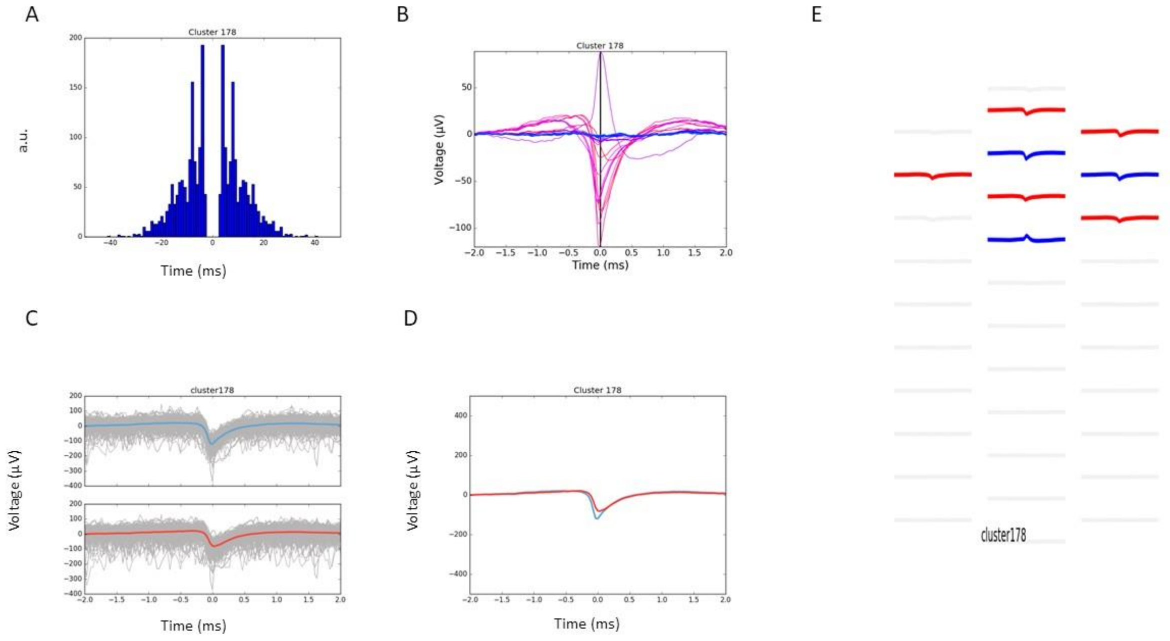

2014-11-13T15_35_31.bin

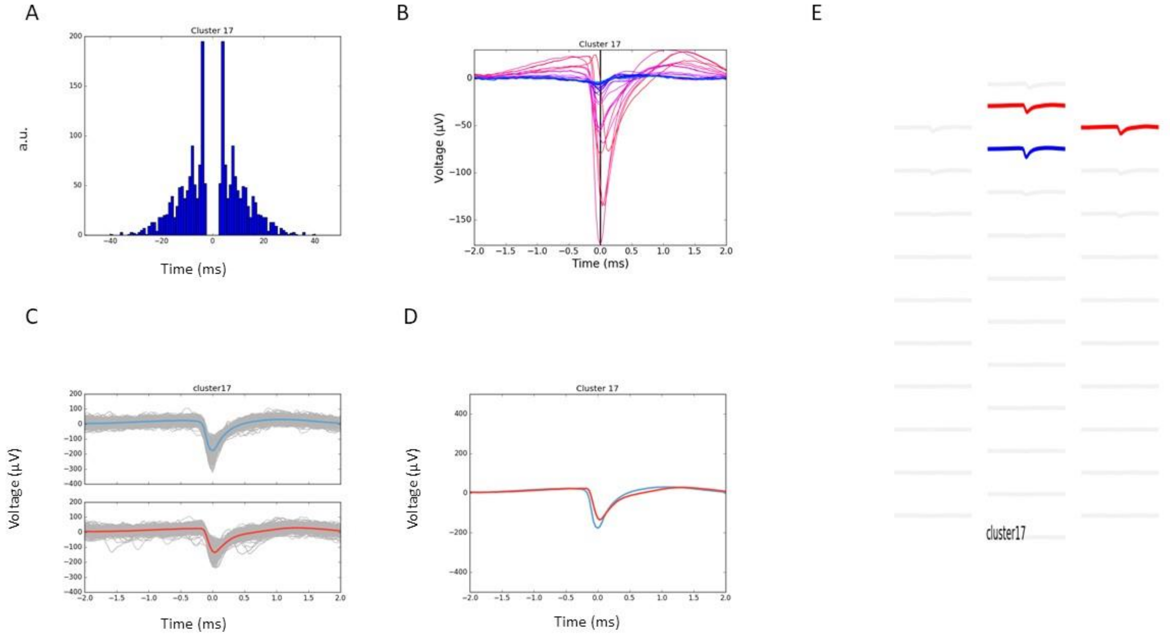

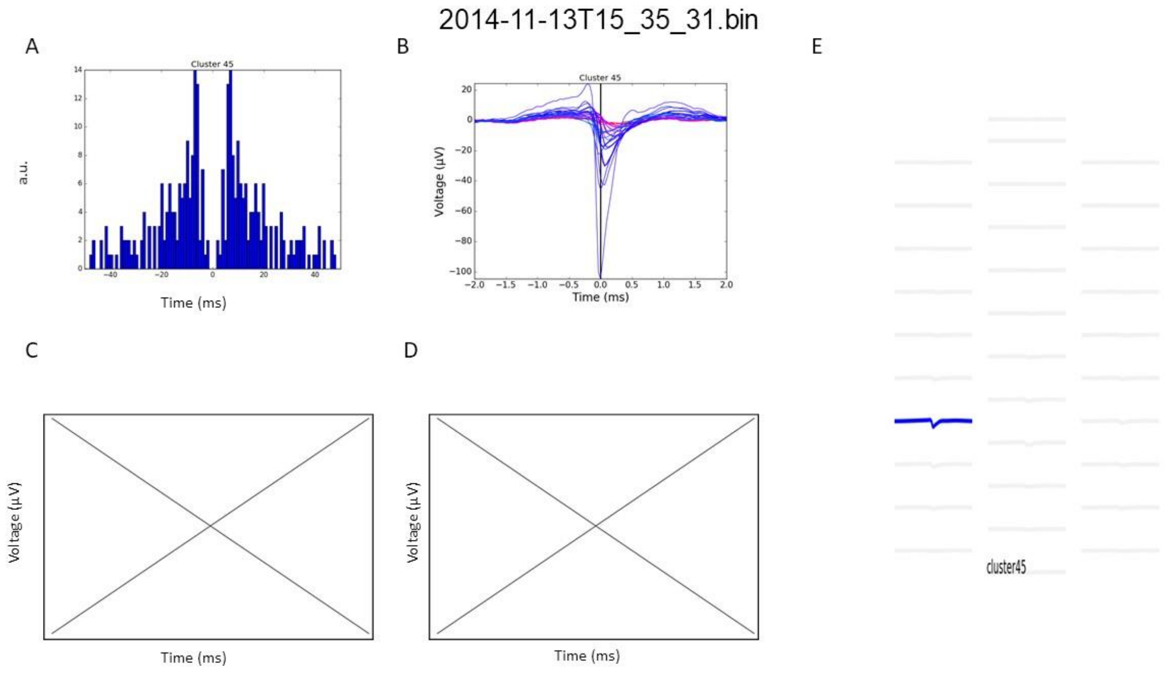
2014-11-13T15_35_31.bin
A
Cluster 45
a.u.
Time (ms)
B
Cluster 45
Voltage (μV)
Time (ms)
E
C
Voltage (μV)
Time (ms)
D
Voltage (μV)
Time (ms)
cluster45

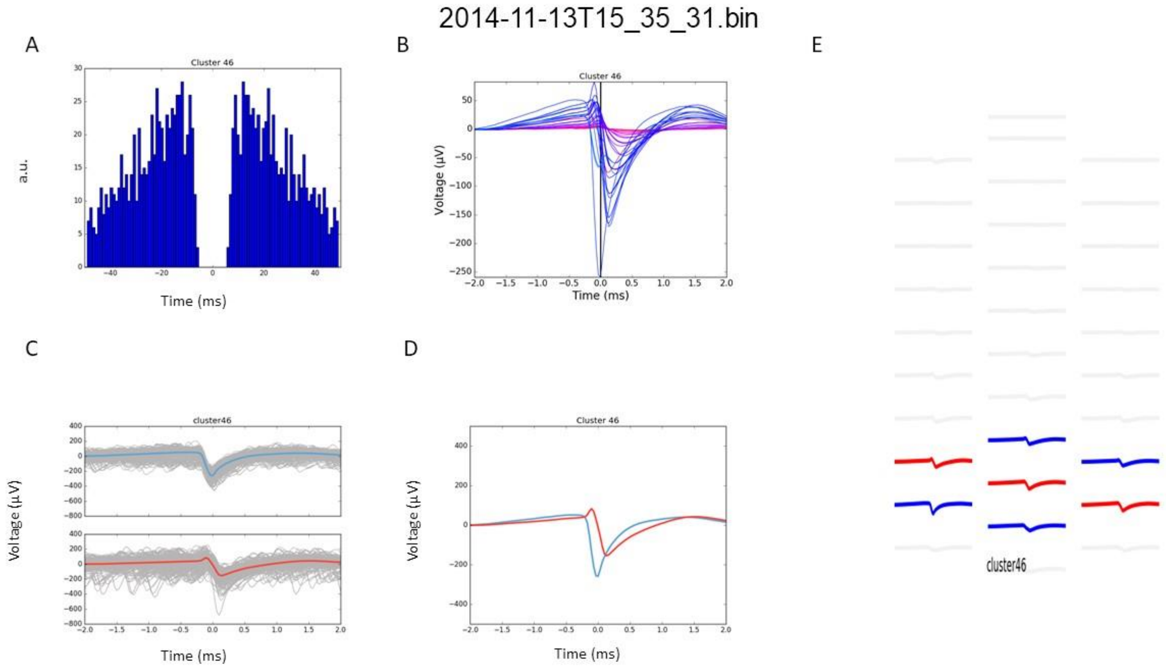
2014-11-13T15_35_31.bin
A
Cluster 46
a.u.
Time (ms)
B
Cluster 46
Voltage (μV)
Time (ms)
E
C
cluster46
Voltage (μV)
Time (ms)
D
Cluster 46
Voltage (μV)
Time (ms)
cluster46

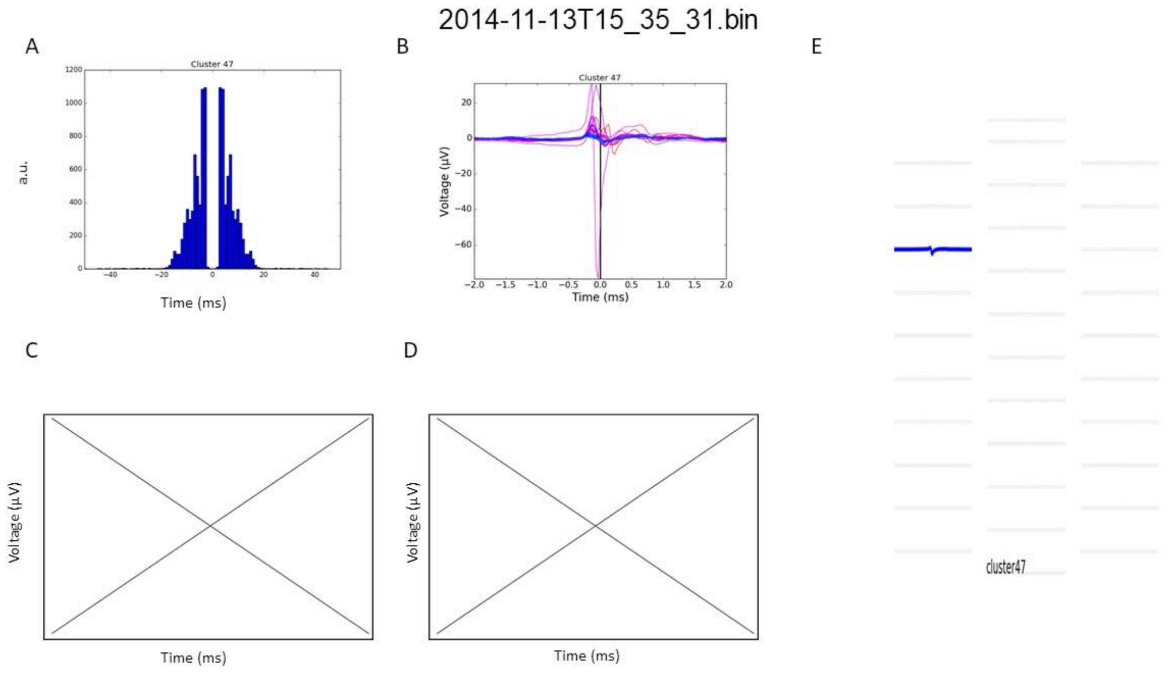
2014-11-13T15_35_31.bin
A
B
E
Cluster 47
a.u.
Time (ms)
Voltage (µV)
C
D
cluster47

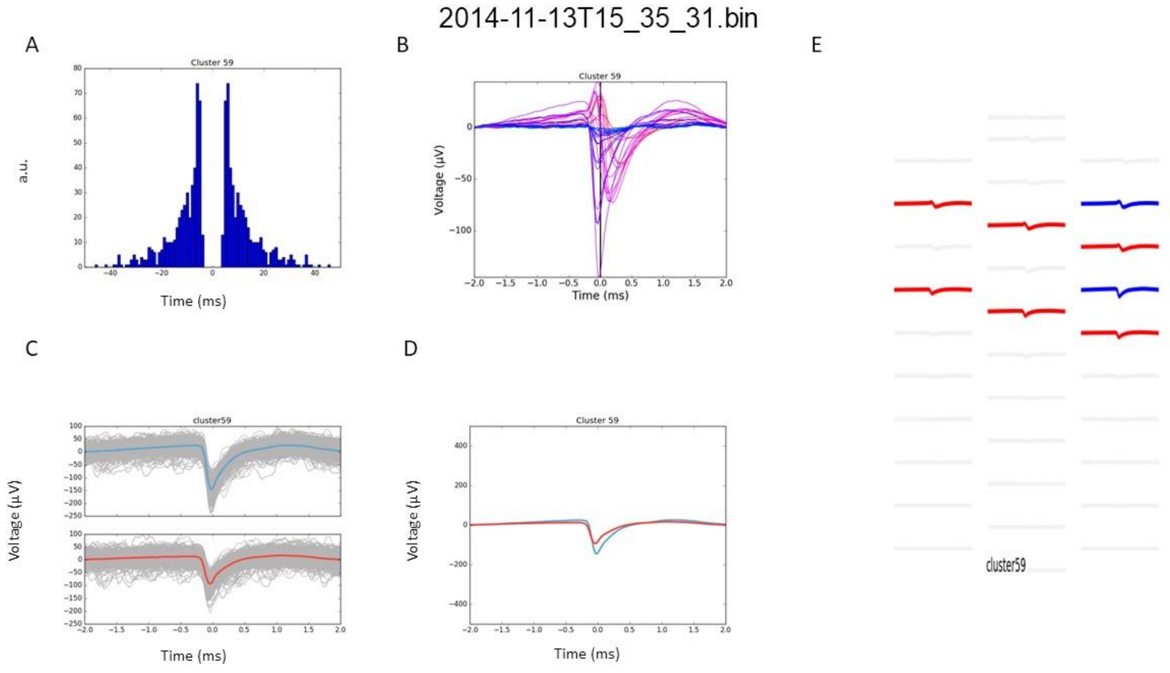
2014-11-13T15_35_31.bin
A
B
E
Cluster 59
a.u.
Time (ms)
Voltage (µV)
C
D
cluster59

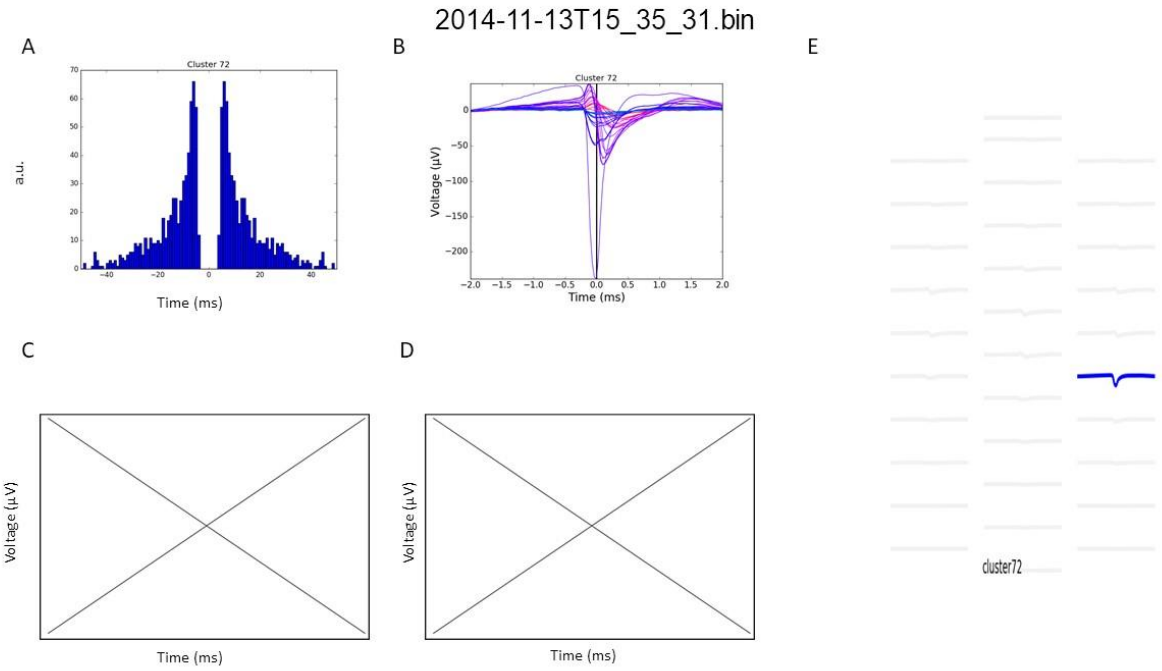
2014-11-13T15_35_31.bin
A
Cluster 72
a.u.
Time (ms)
B
Cluster 72
Voltage (μV)
Time (ms)
E
cluster72
C
Voltage (μV)
Time (ms)
D
Voltage (μV)
Time (ms)

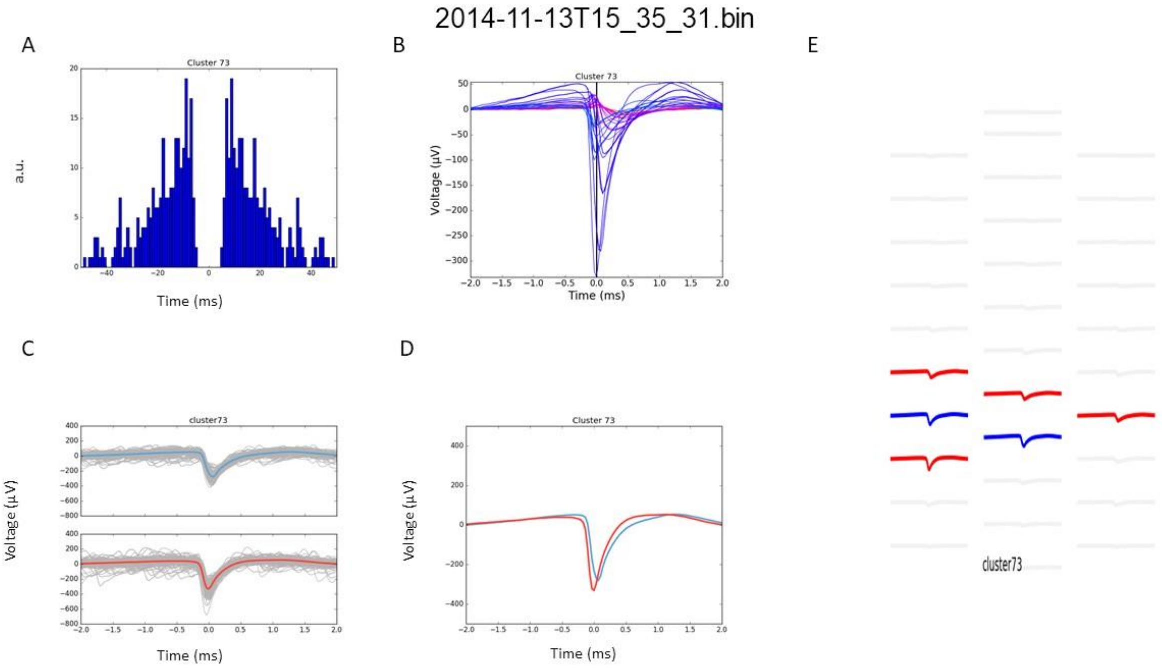
2014-11-13T15_35_31.bin
A
Cluster 73
a.u.
Time (ms)
B
Cluster 73
Voltage (μV)
Time (ms)
E
cluster73
C
cluster73
Voltage (μV)
Time (ms)
D
Cluster 73
Voltage (μV)
Time (ms)

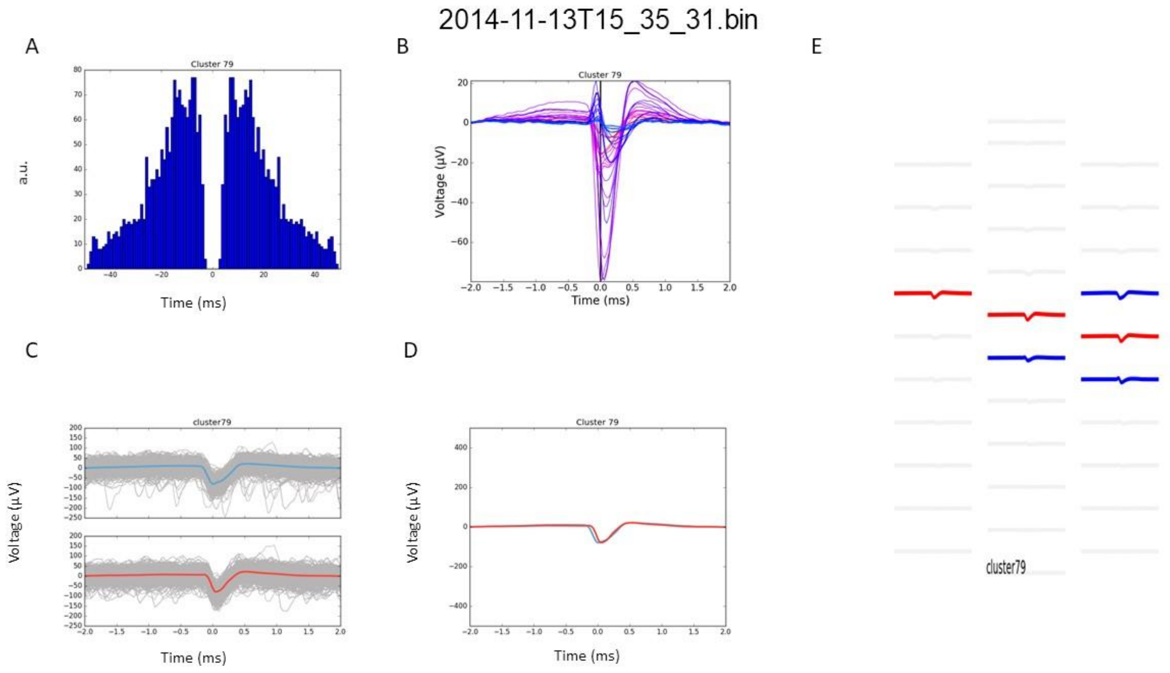
2014-11-13T15_35_31.bin
A
Cluster 79
a.u.
Time (ms)
B
Cluster 79
Voltage (μV)
Time (ms)
C
cluster79
Voltage (μV)
Time (ms)
D
Cluster 79
Voltage (μV)
Time (ms)
E
cluster79

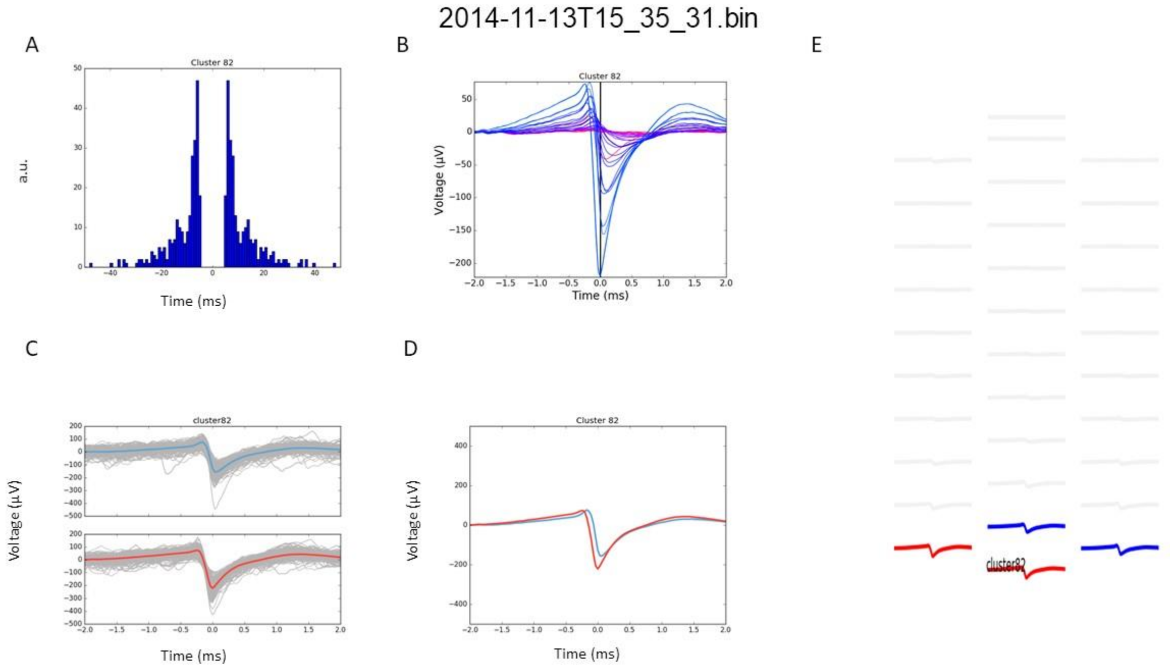
2014-11-13T15_35_31.bin
A
Cluster 82
a.u.
Time (ms)
B
Cluster 82
Voltage (μV)
Time (ms)
C
cluster82
Voltage (μV)
Time (ms)
D
Cluster 82
Voltage (μV)
Time (ms)
E
cluster82

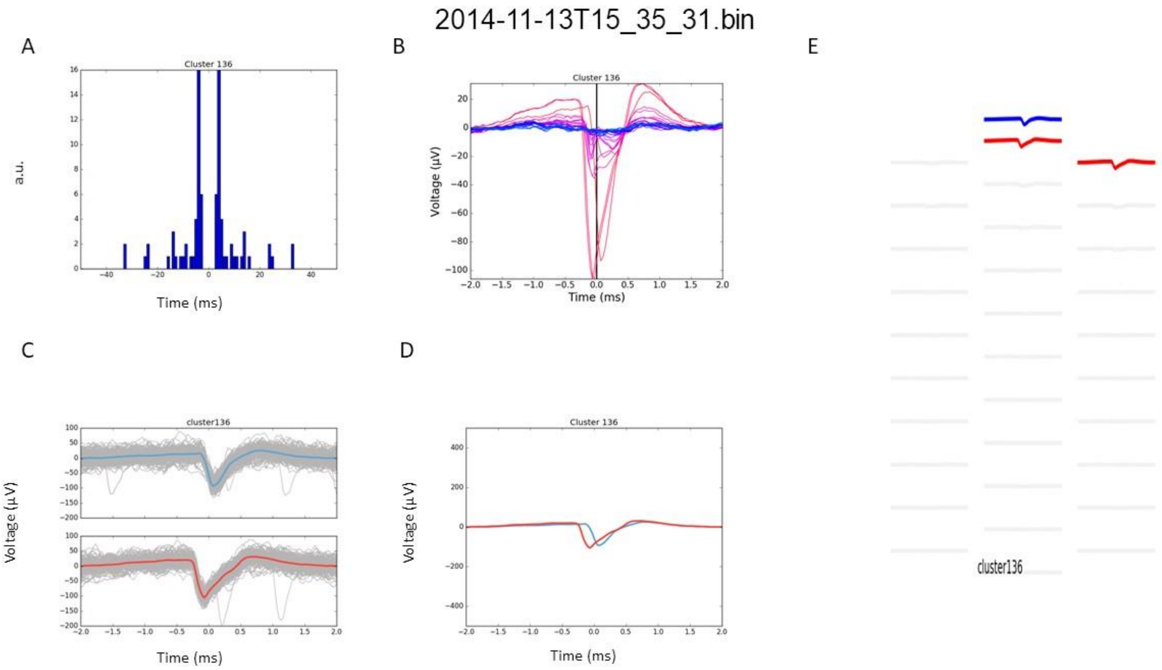
2014-11-13T15_35_31.bin
A
B
E
Cluster 136
a.u.
Time (ms)
Voltage (µV)
C
D
cluster136
Voltage (µV)
Time (ms)
Cluster 136
Voltage (µV)
Time (ms)
cluster136

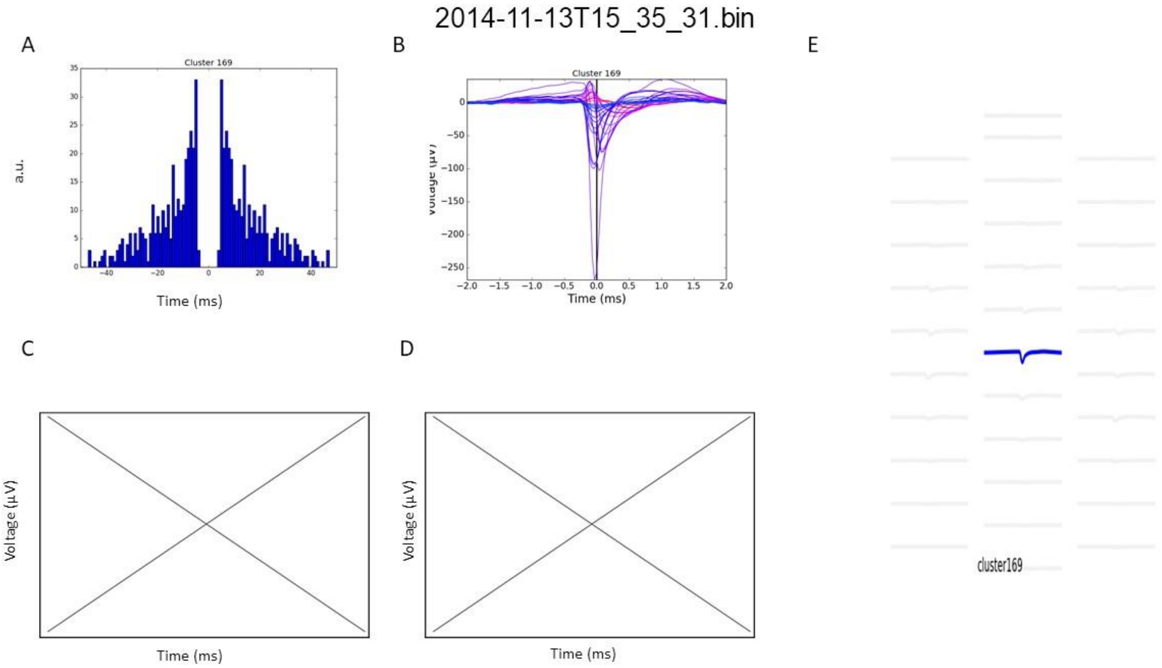
2014-11-13T15_35_31.bin
A
B
E
Cluster 169
a.u.
Time (ms)
Cluster 169
Time (ms)
C
D
Voltage (µV)
Time (ms)
Voltage (µV)
Time (ms)
cluster169

2014-11-13T15_35_31.bin

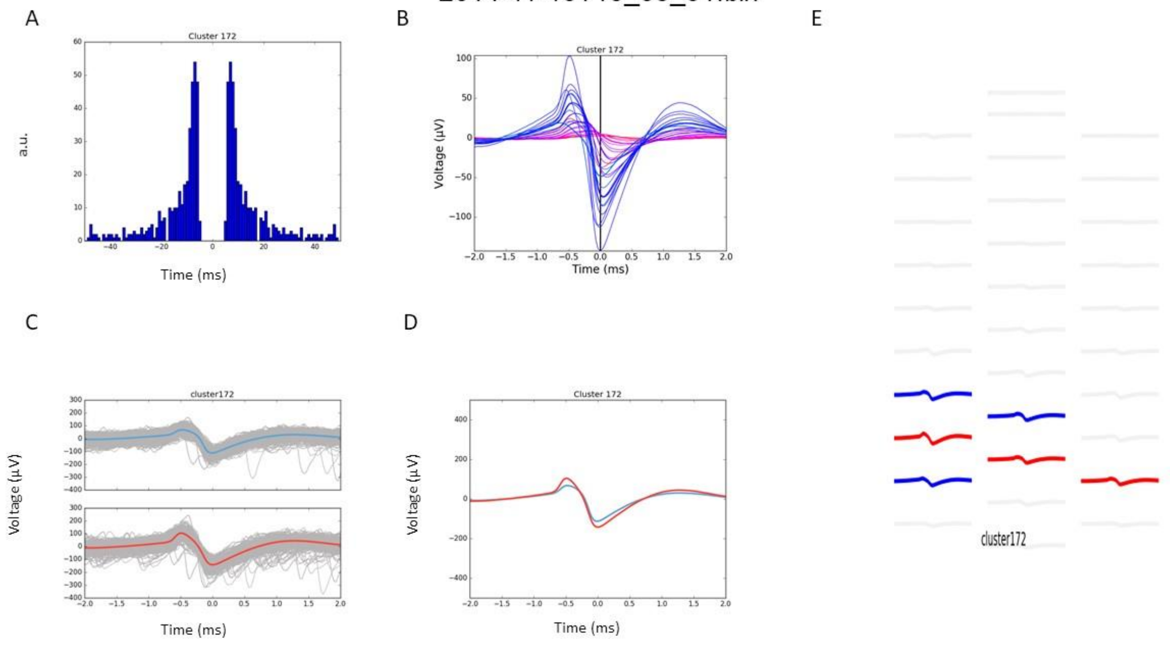

2014-11-13T15_35_31.bin

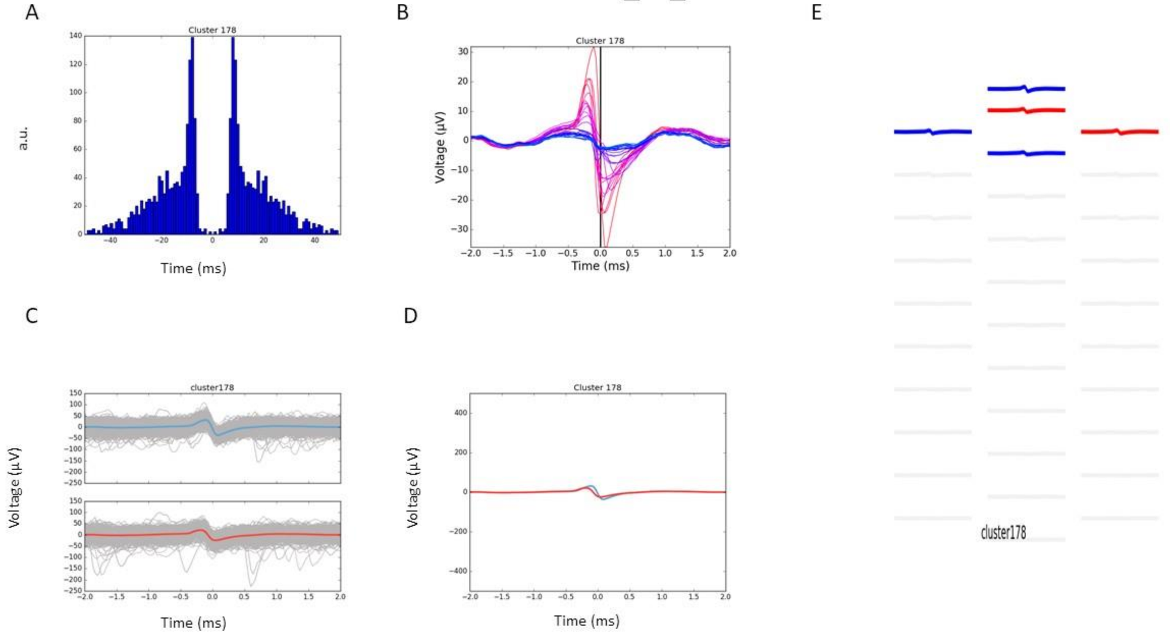

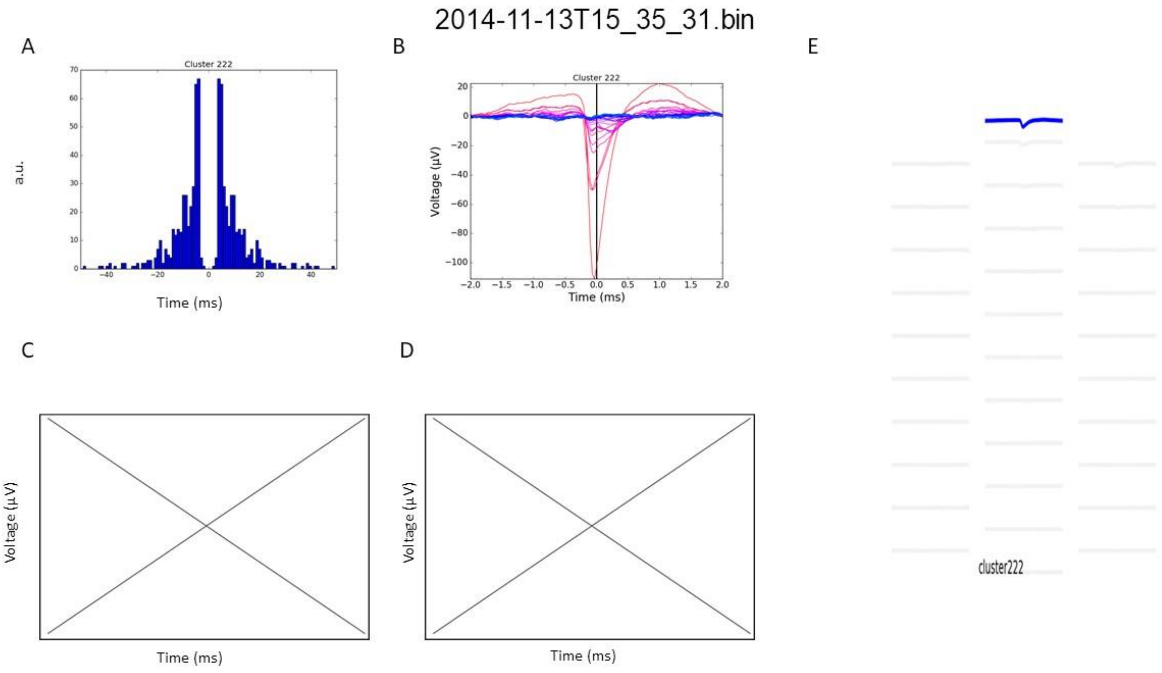
2014-11-13T15_35_31.bin
A
Cluster 222
a.u.
Time (ms)
B
Cluster 222
Voltage (µV)
Time (ms)
C
Voltage (µV)
Time (ms)
D
Voltage (µV)
Time (ms)
E
cluster222

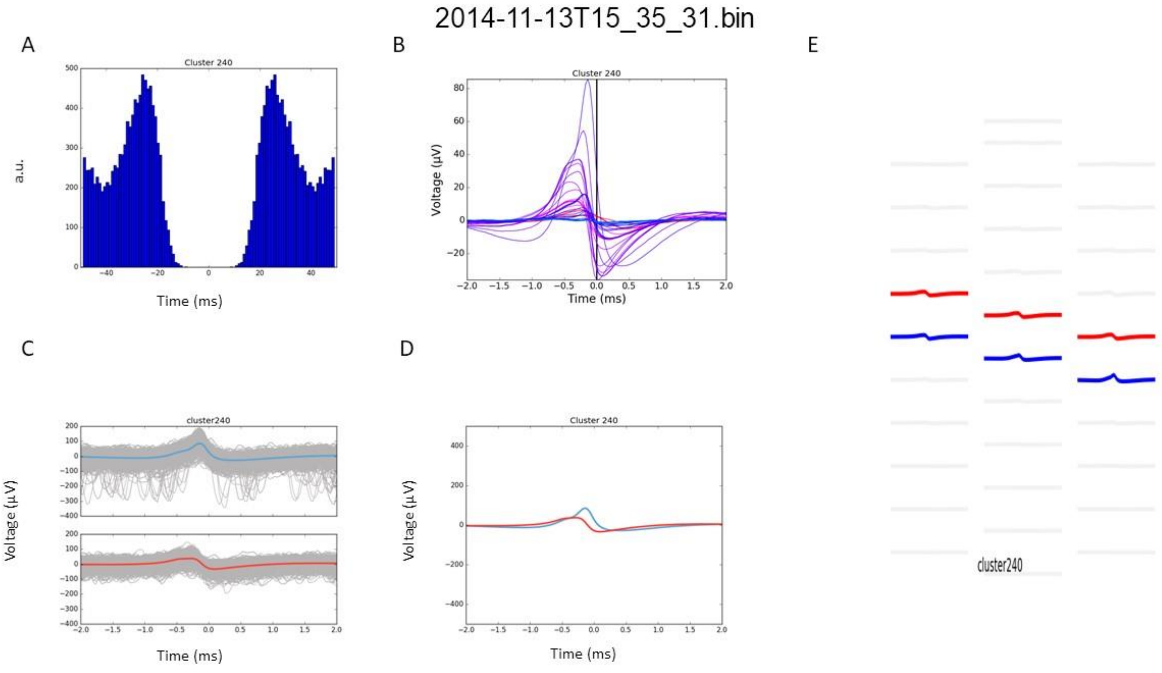
2014-11-13T15_35_31.bin
A
Cluster 240
a.u.
Time (ms)
B
Cluster 240
Voltage (µV)
Time (ms)
C
cluster240
Voltage (µV)
Time (ms)
D
Cluster 240
Voltage (µV)
Time (ms)
E
cluster240

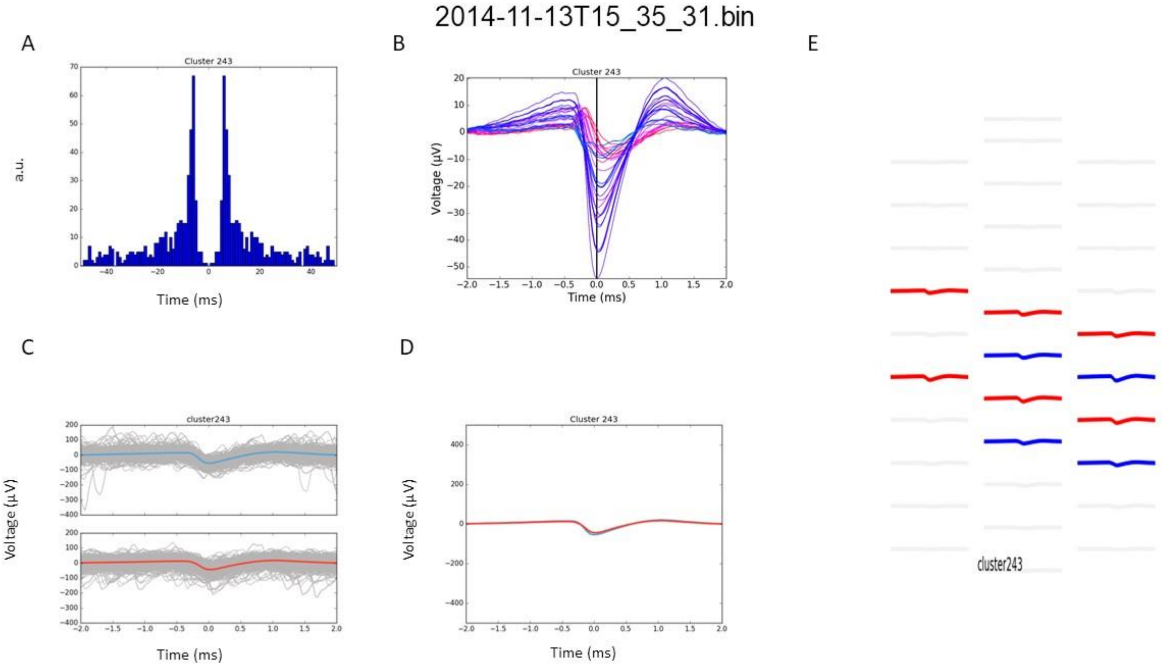
2014-11-13T15_35_31.bin
A
Cluster 243
a.u.
Time (ms)
B
Cluster 243
Voltage (μV)
Time (ms)
C
cluster243
Voltage (μV)
Time (ms)
D
Cluster 243
Voltage (μV)
Time (ms)
E
cluster243

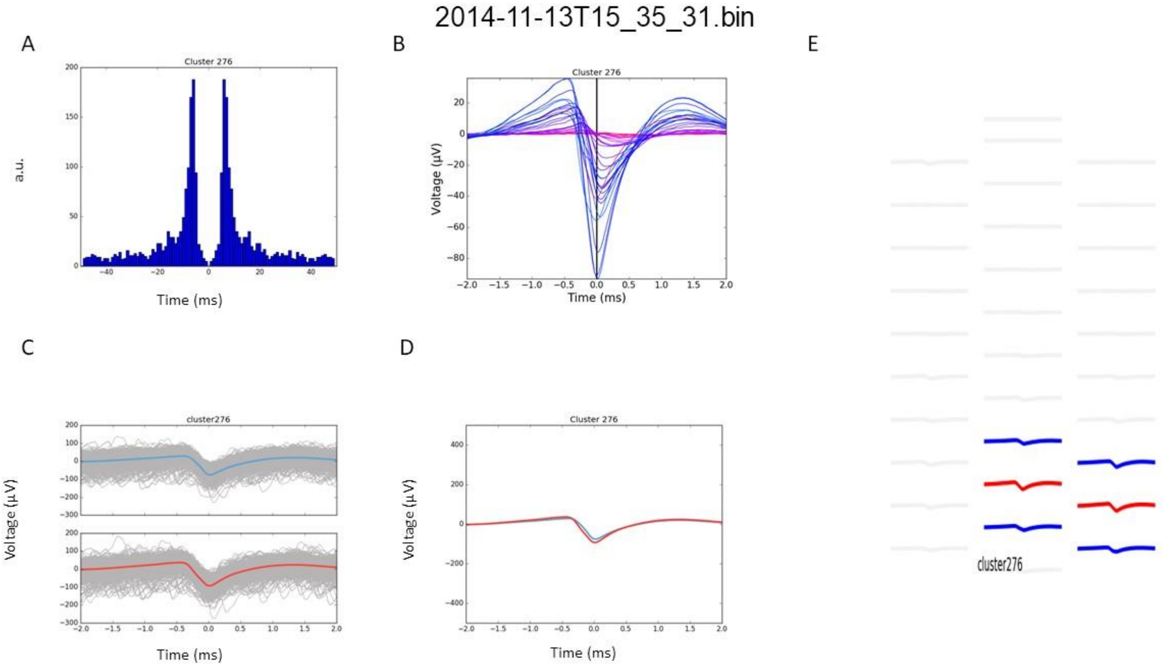
2014-11-13T15_35_31.bin
A
Cluster 276
a.u.
Time (ms)
B
Cluster 276
Voltage (μV)
Time (ms)
C
cluster276
Voltage (μV)
Time (ms)
D
Cluster 276
Voltage (μV)
Time (ms)
E
cluster276

2014-11-13T18_05_50.bin

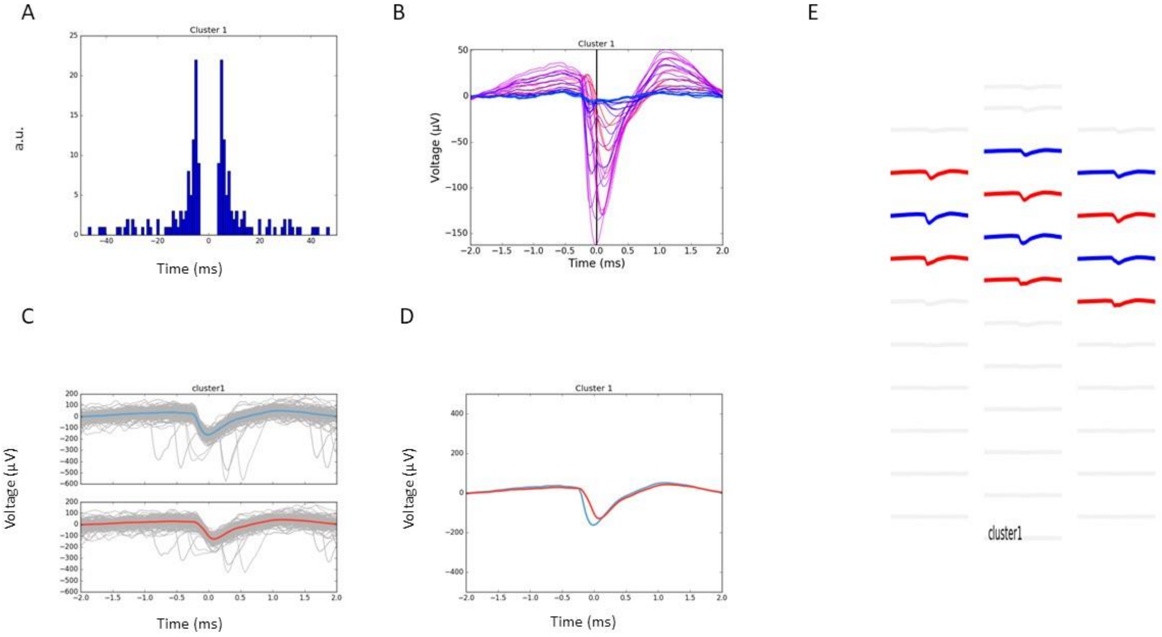

2014-11-13T18_05_50.bin

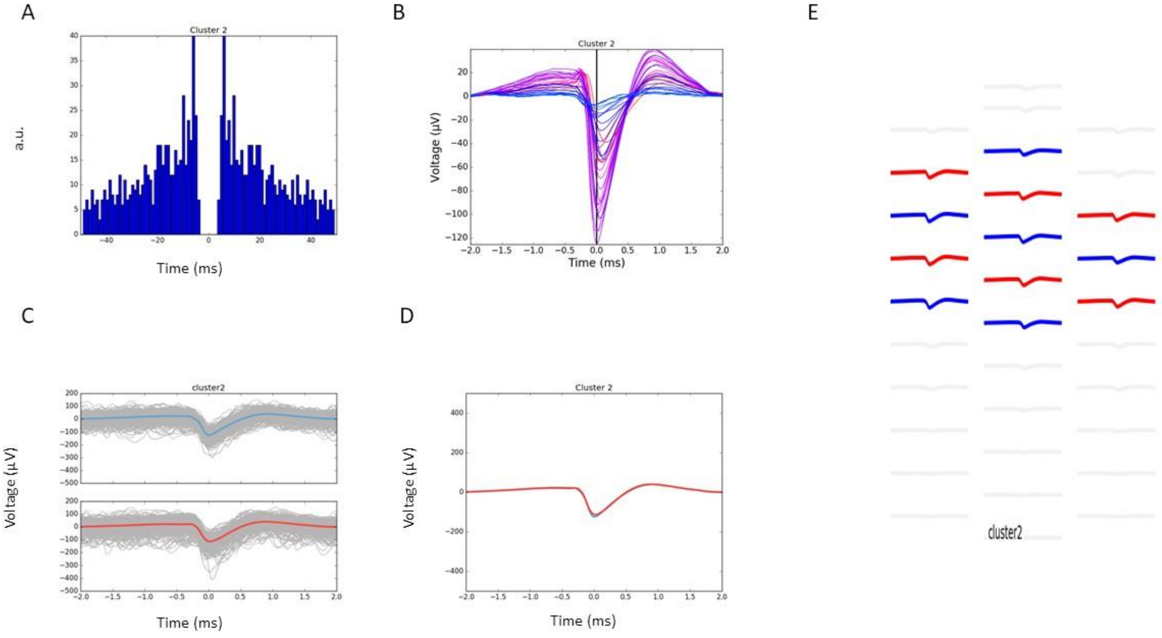

2014-11-13T18_05_50.bin

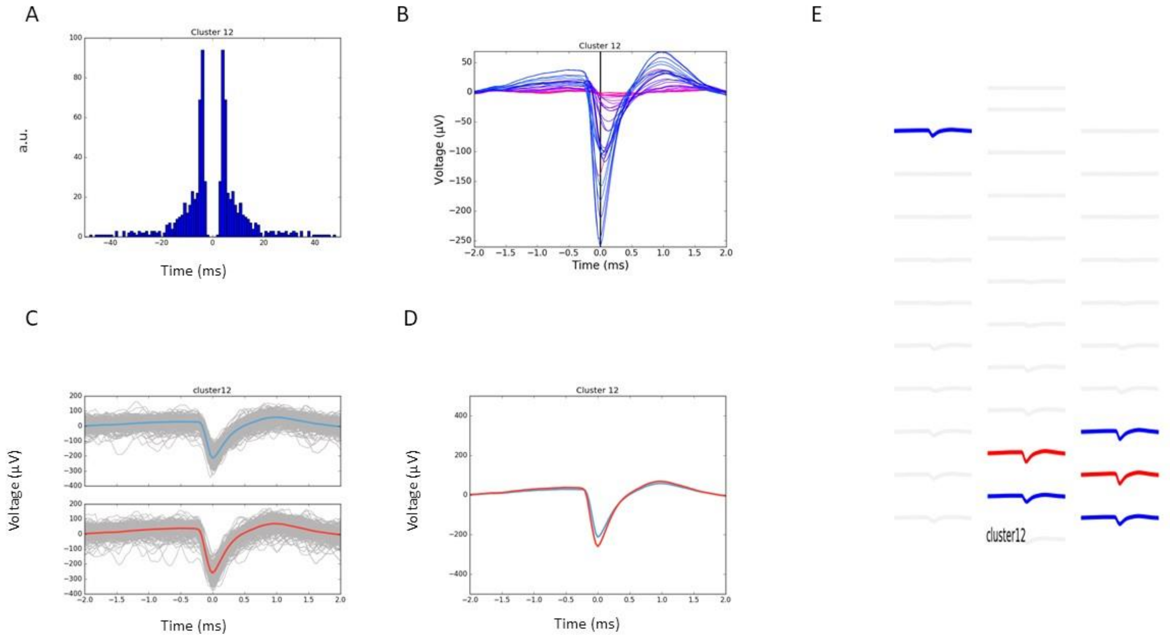

2014-11-13T18_05_50.bin

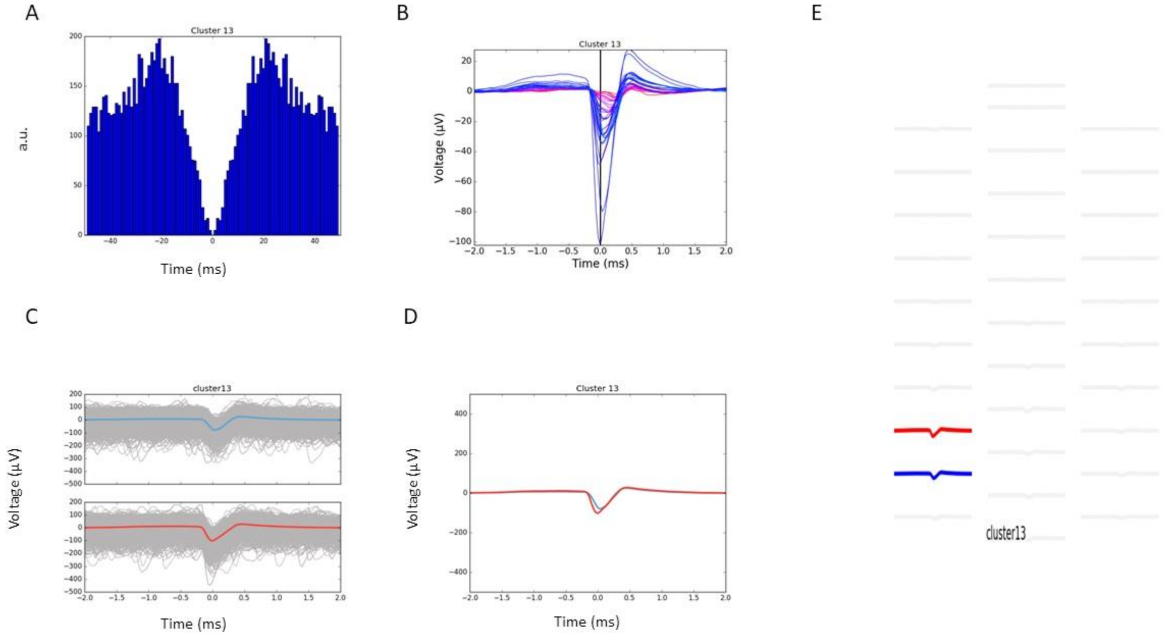

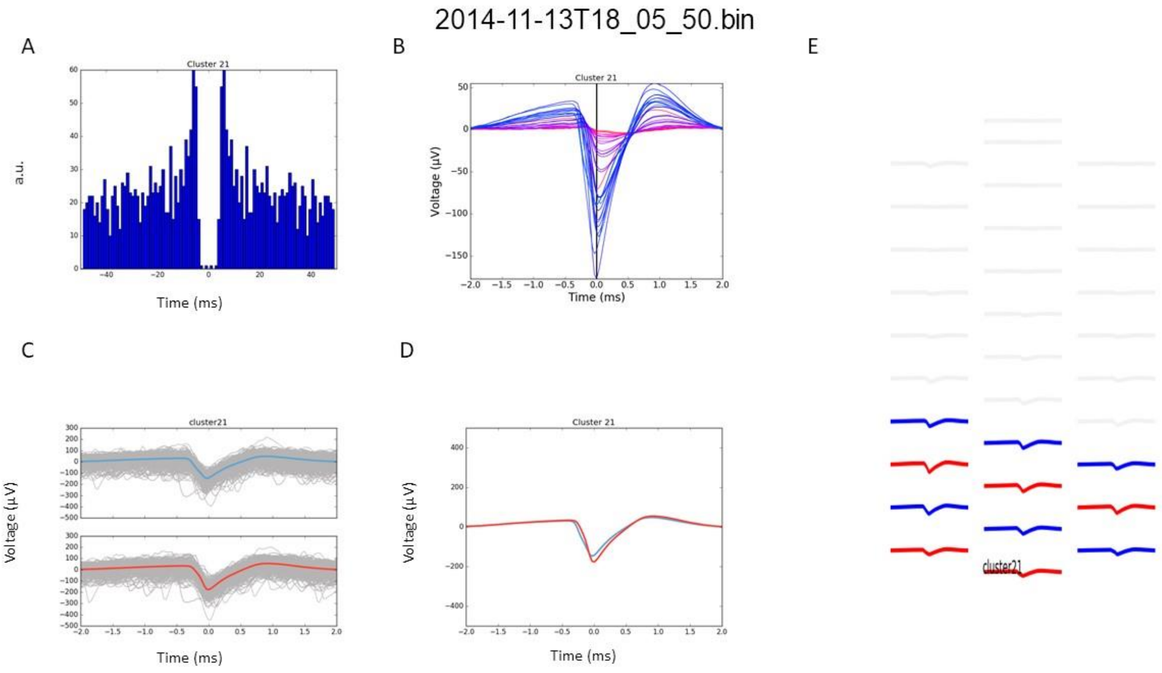
2014-11-13T18_05_50.bin
A
Cluster 21
a.u.
Time (ms)
B
Cluster 21
Voltage (μV)
Time (ms)
C
cluster21
Voltage (μV)
Time (ms)
D
Cluster 21
Voltage (μV)
Time (ms)
E
cluster21

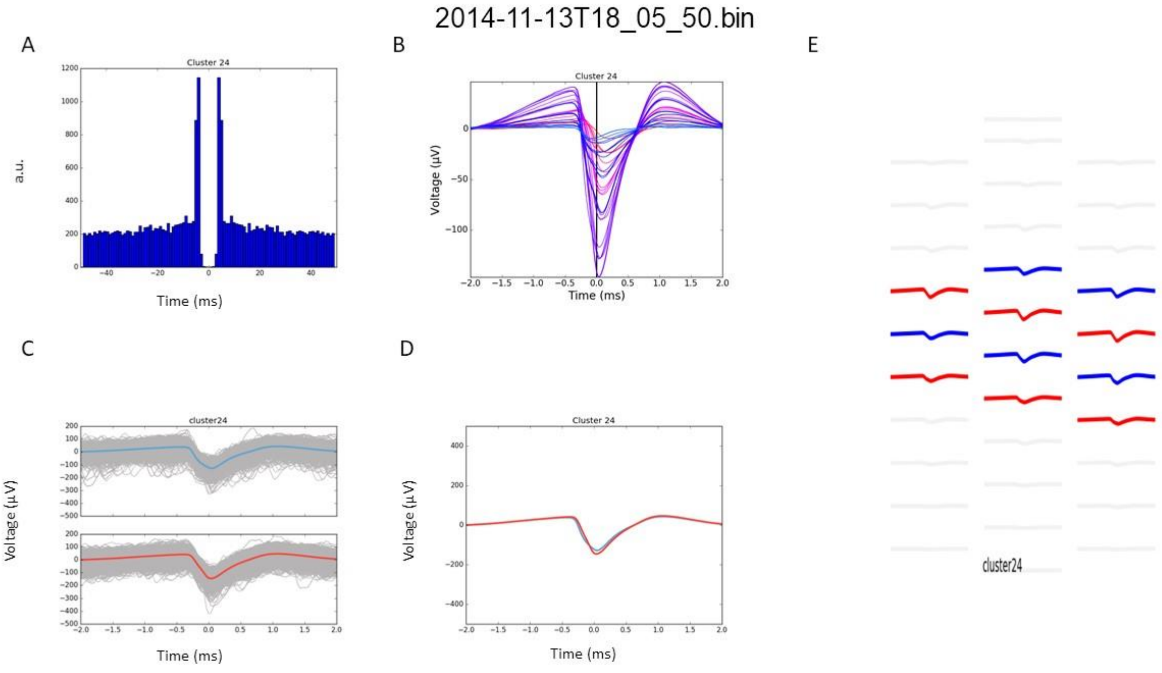
2014-11-13T18_05_50.bin
A
Cluster 24
a.u.
Time (ms)
B
Cluster 24
Voltage (μV)
Time (ms)
C
cluster24
Voltage (μV)
Time (ms)
D
Cluster 24
Voltage (μV)
Time (ms)
E
cluster24

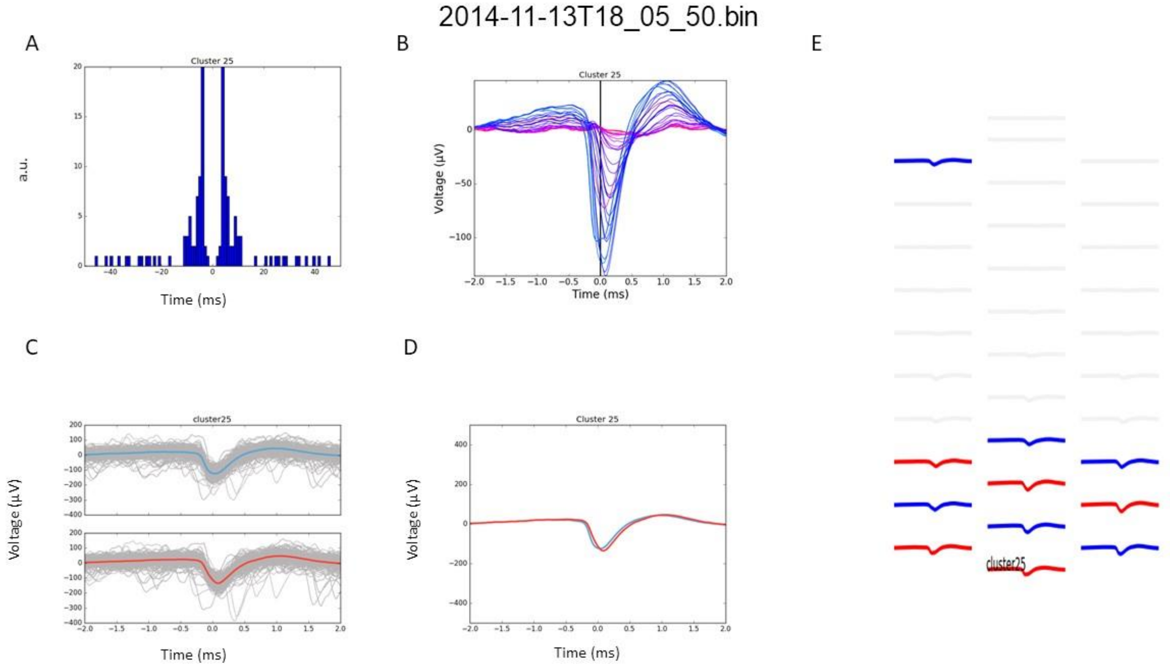
2014-11-13T18_05_50.bin
A
B
C
D
E
Cluster 25
a.u.
Time (ms)
Voltage (µV)
cluster25

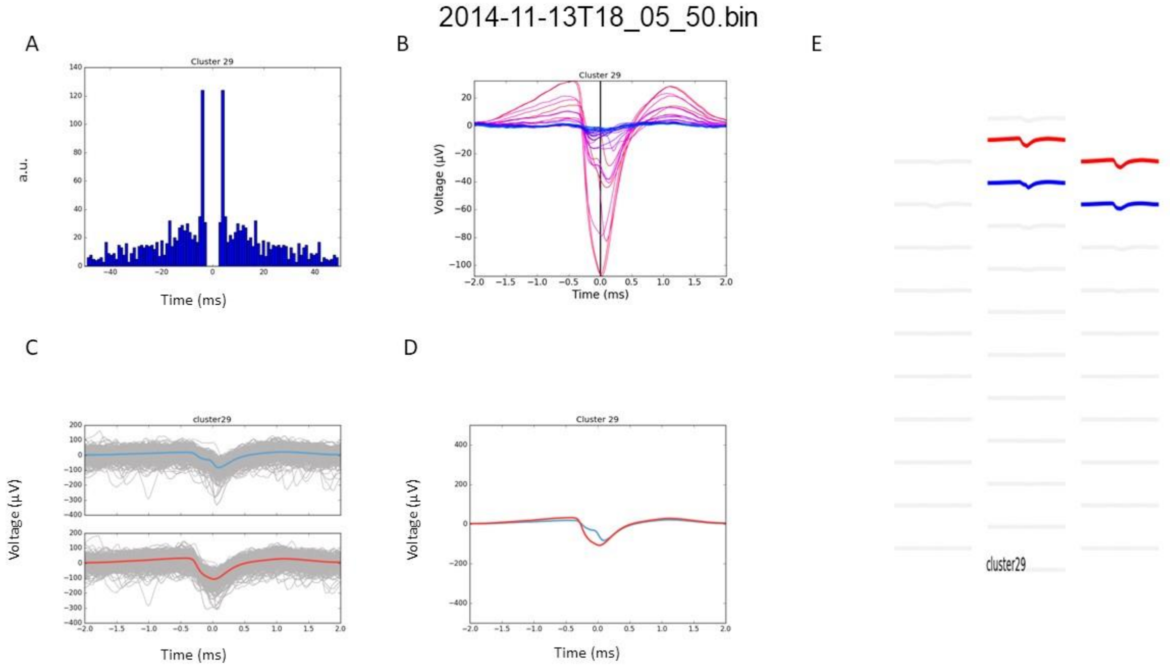
2014-11-13T18_05_50.bin
A
B
C
D
E
Cluster 29
a.u.
Time (ms)
Voltage (µV)
cluster29

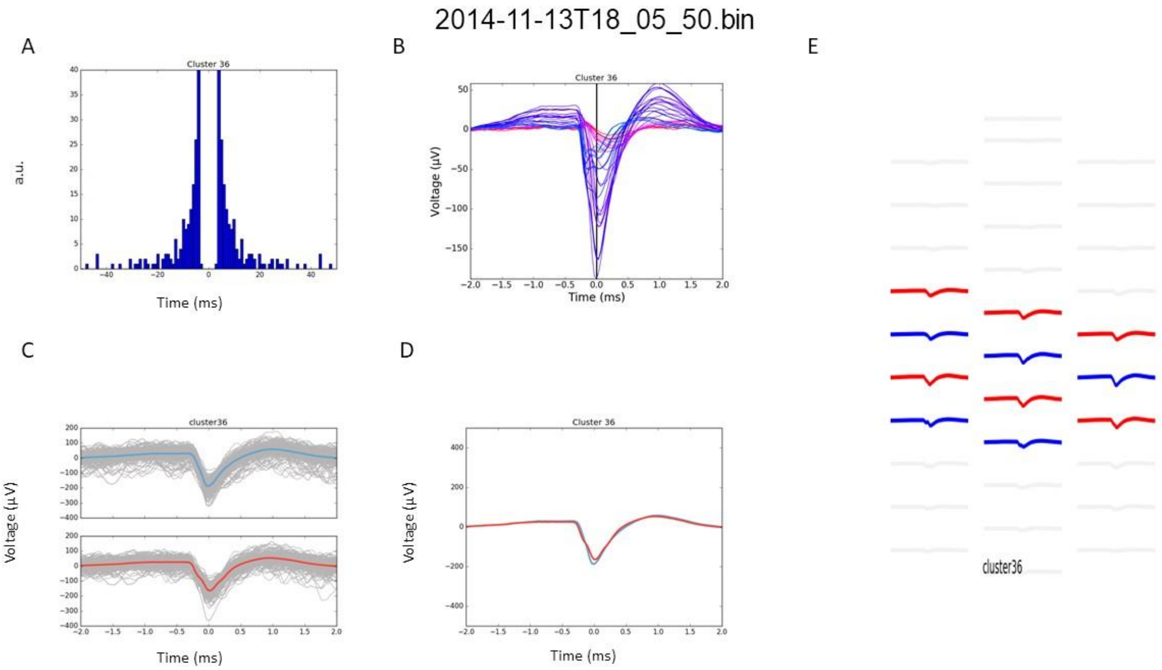
2014-11-13T18_05_50.bin
A
B
E
Cluster 36
a.u.
Time (ms)
Voltage (µV)
C
D
cluster36
Cluster 36
cluster36

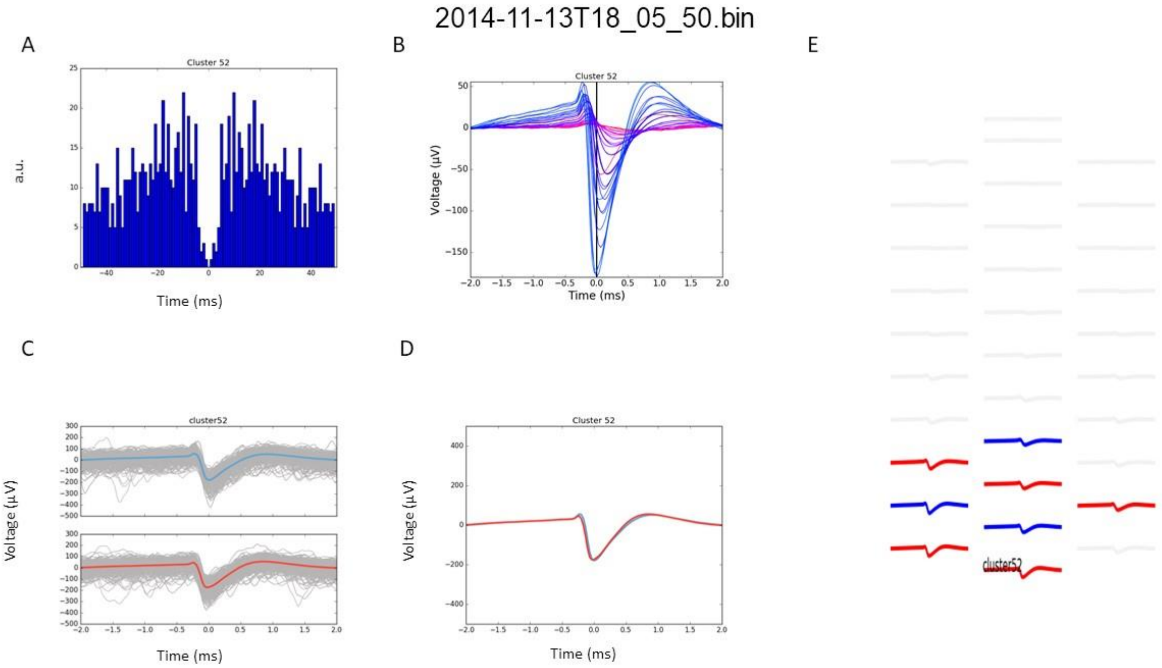
2014-11-13T18_05_50.bin
A
B
E
Cluster 52
a.u.
Time (ms)
Voltage (µV)
C
D
cluster52
Cluster 52
cluster52

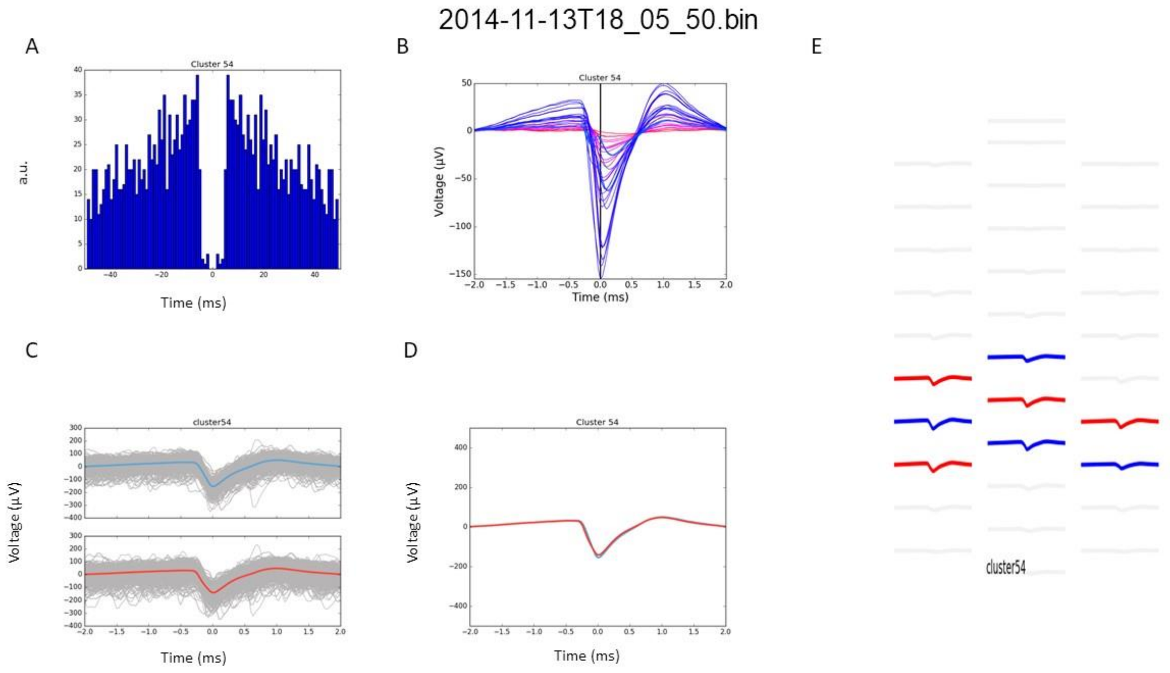
2014-11-13T18_05_50.bin
A
Cluster 54
a.u.
Time (ms)
B
Cluster 54
Voltage (µV)
Time (ms)
E
C
cluster54
Voltage (µV)
Time (ms)
D
Cluster 54
Voltage (µV)
Time (ms)
cluster54

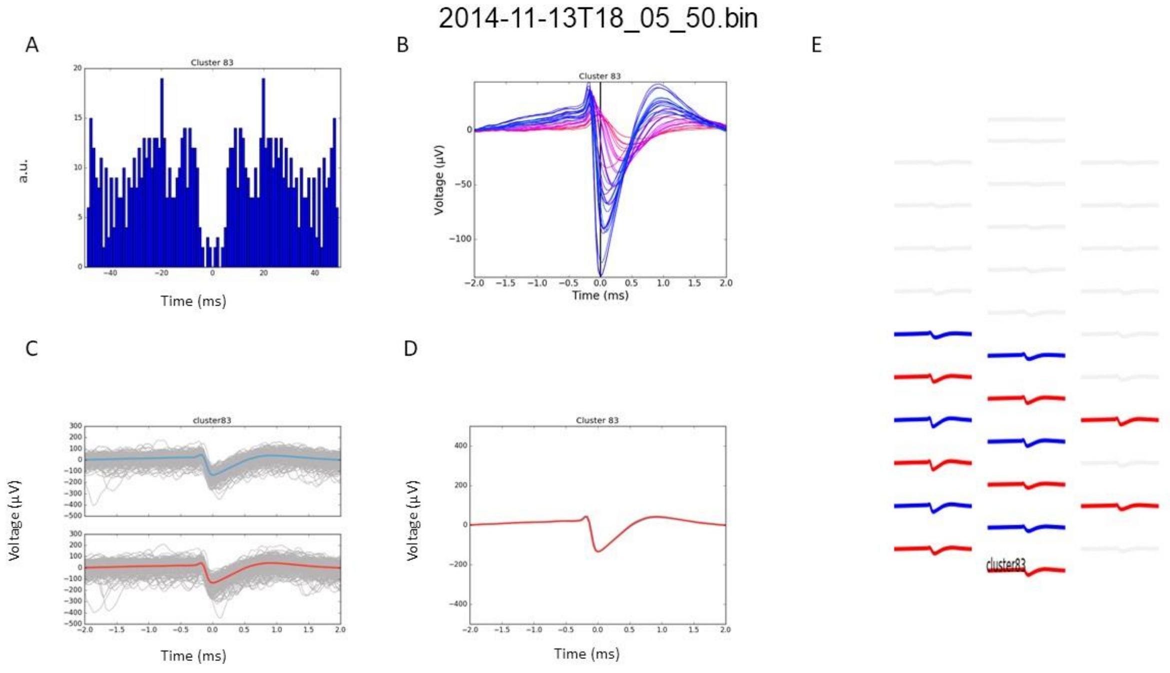
2014-11-13T18_05_50.bin
A
Cluster 83
a.u.
Time (ms)
B
Cluster 83
Voltage (µV)
Time (ms)
E
C
cluster83
Voltage (µV)
Time (ms)
D
Cluster 83
Voltage (µV)
Time (ms)
cluster83

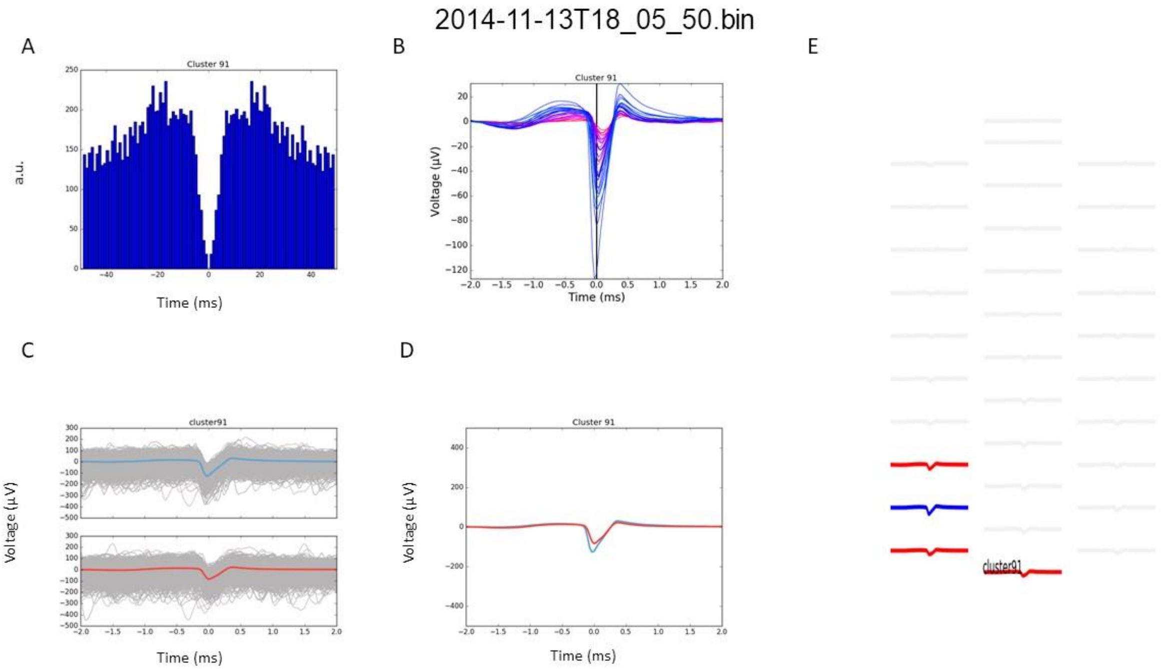
2014-11-13T18_05_50.bin
A
B
E
Cluster 91
a.u.
Time (ms)
Voltage (µV)
C
D
cluster91
Cluster 91

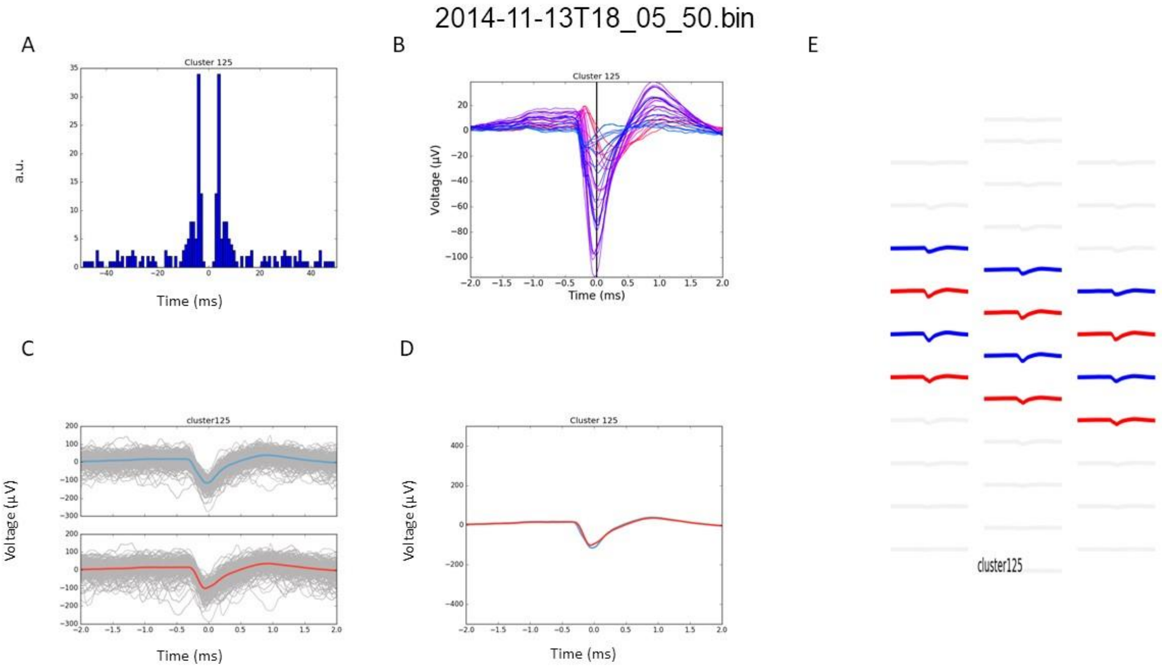
2014-11-13T18_05_50.bin
A
B
E
Cluster 125
a.u.
Time (ms)
Voltage (µV)
C
D
cluster125
Cluster 125

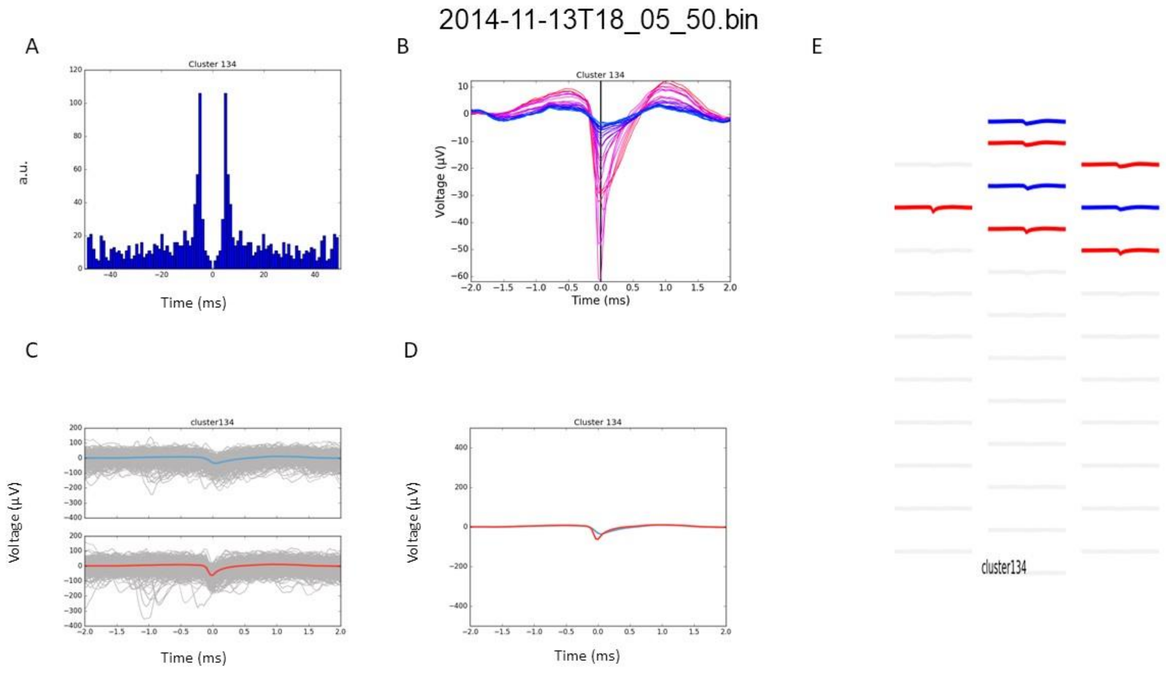
2014-11-13T18_05_50.bin
A
Cluster 134
a.u.
Time (ms)
B
Cluster 134
Voltage (µV)
Time (ms)
E
cluster134
C
cluster134
Voltage (µV)
Time (ms)
D
Cluster 134
Voltage (µV)
Time (ms)

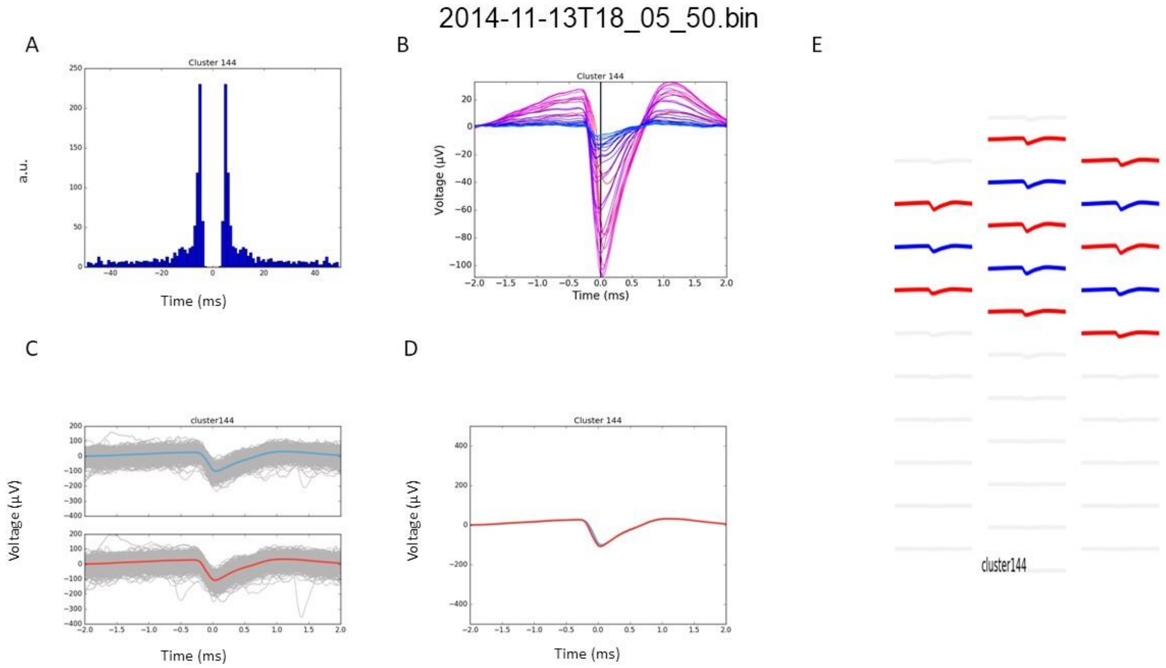
2014-11-13T18_05_50.bin
A
Cluster 144
a.u.
Time (ms)
B
Cluster 144
Voltage (µV)
Time (ms)
E
cluster144
C
cluster144
Voltage (µV)
Time (ms)
D
Cluster 144
Voltage (µV)
Time (ms)

2014-11-13T18_05_50.bin

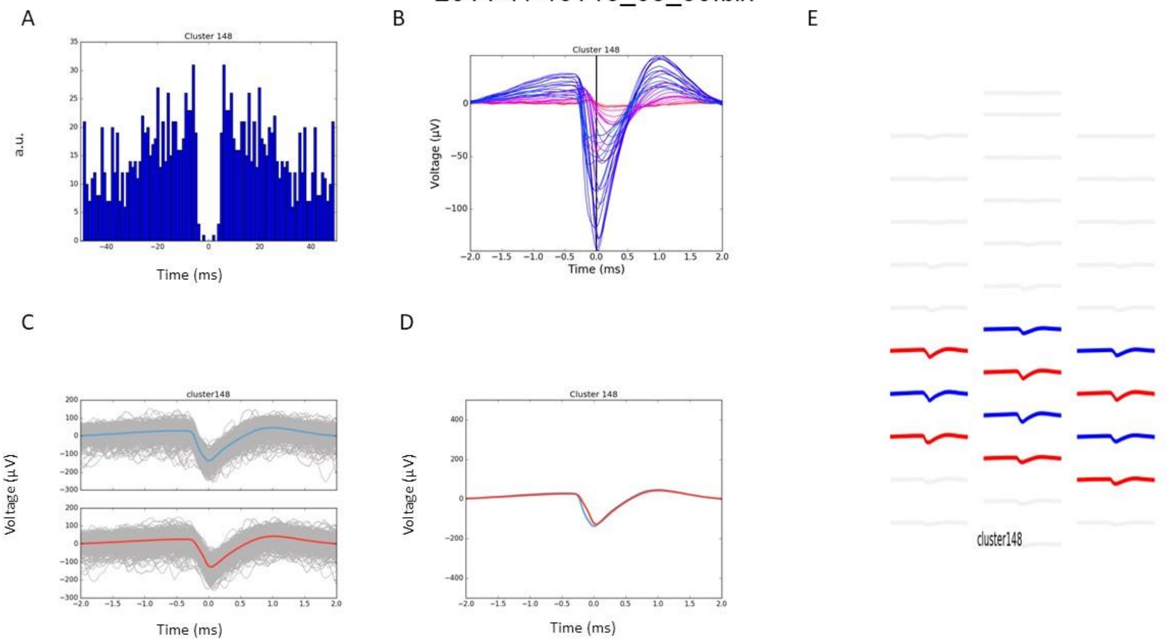

2014-11-13T18_05_50.bin

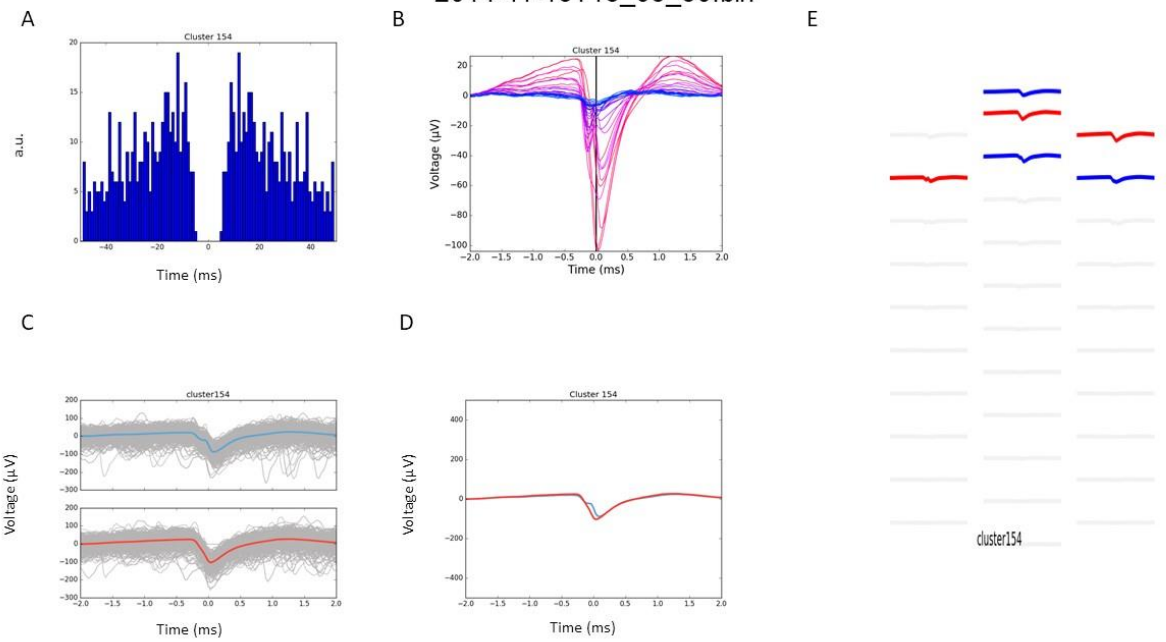

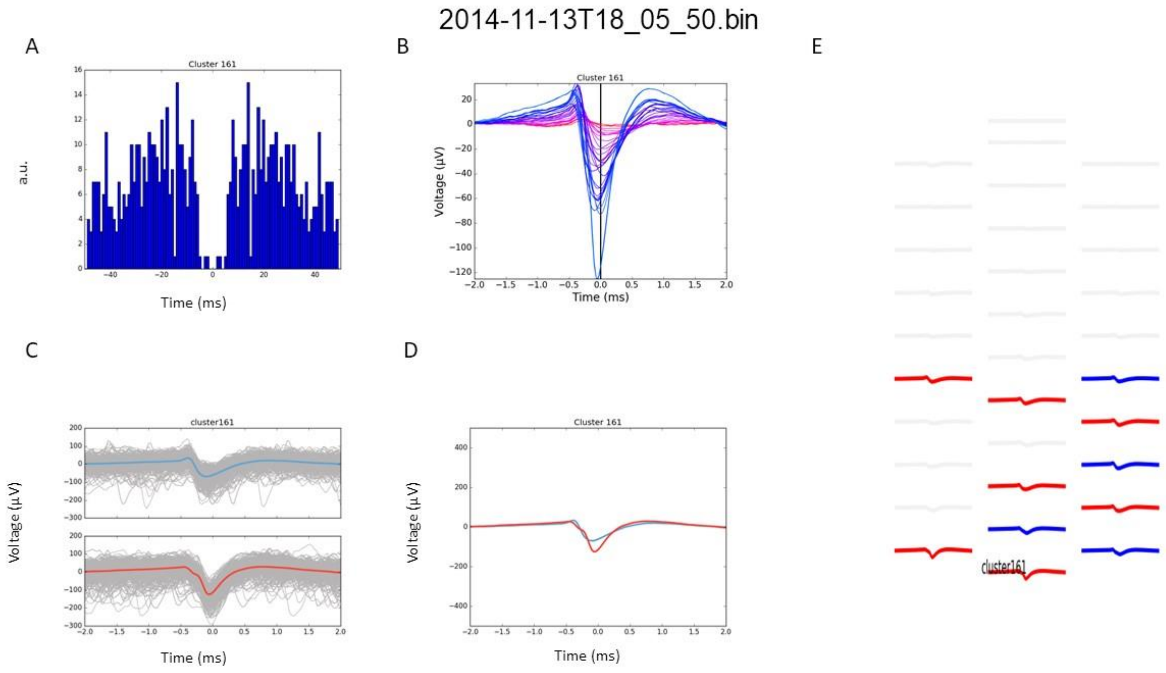
2014-11-13T18_05_50.bin
A
Cluster 161
a.u.
Time (ms)
B
Cluster 161
Voltage (µV)
Time (ms)
E
cluster161
C
cluster161
Voltage (µV)
Time (ms)
D
Cluster 161
Voltage (µV)
Time (ms)

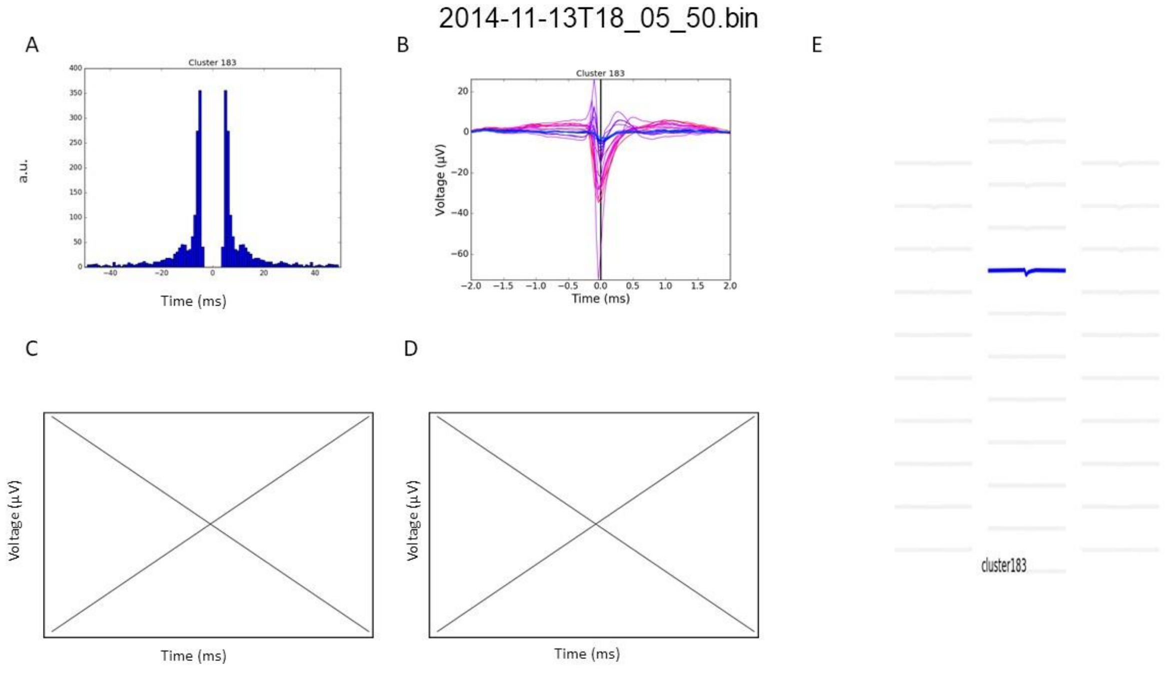
2014-11-13T18_05_50.bin
A
Cluster 183
a.u.
Time (ms)
B
Cluster 183
Voltage (µV)
Time (ms)
E
cluster183
C
Voltage (µV)
Time (ms)
D
Voltage (µV)
Time (ms)

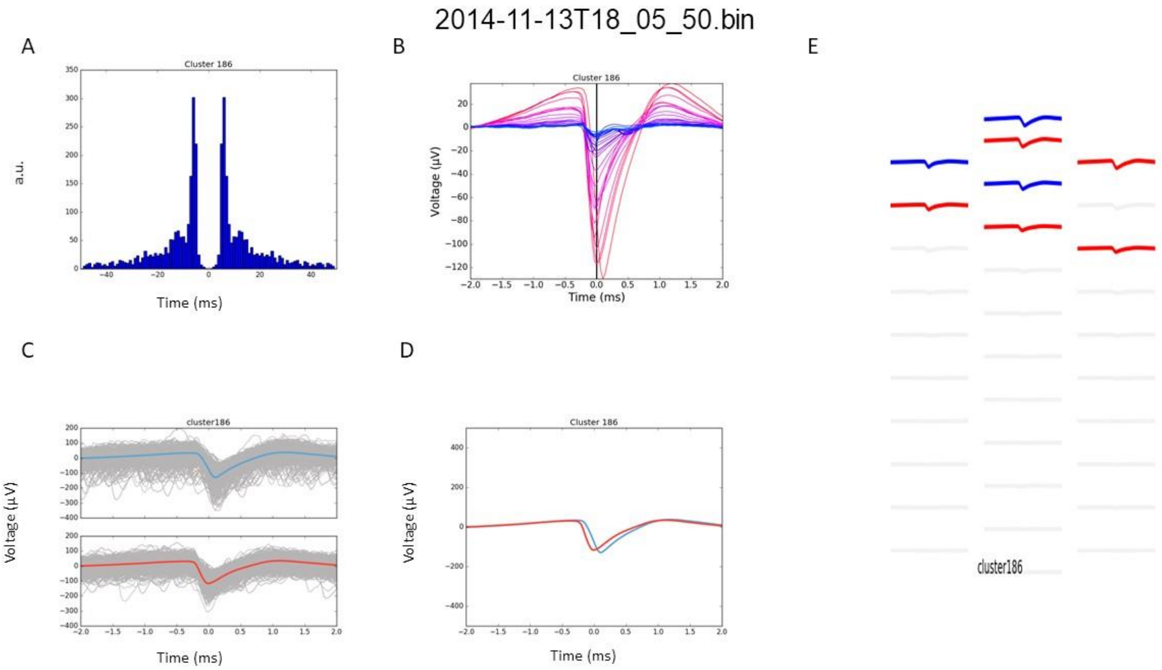
2014-11-13T18_05_50.bin
A
B
E
Cluster 186
a.u.
Time (ms)
Voltage (µV)
C
D
cluster186
Cluster 186
cluster186

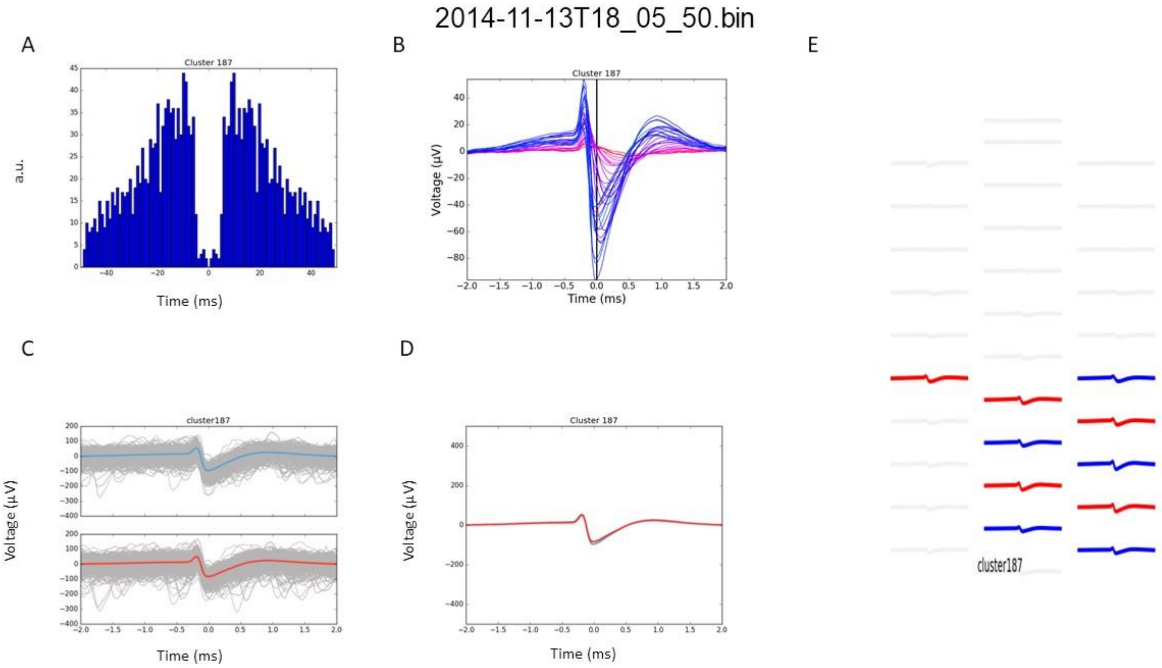
2014-11-13T18_05_50.bin
A
B
E
Cluster 187
a.u.
Time (ms)
Voltage (µV)
C
D
cluster187
Cluster 187
cluster187

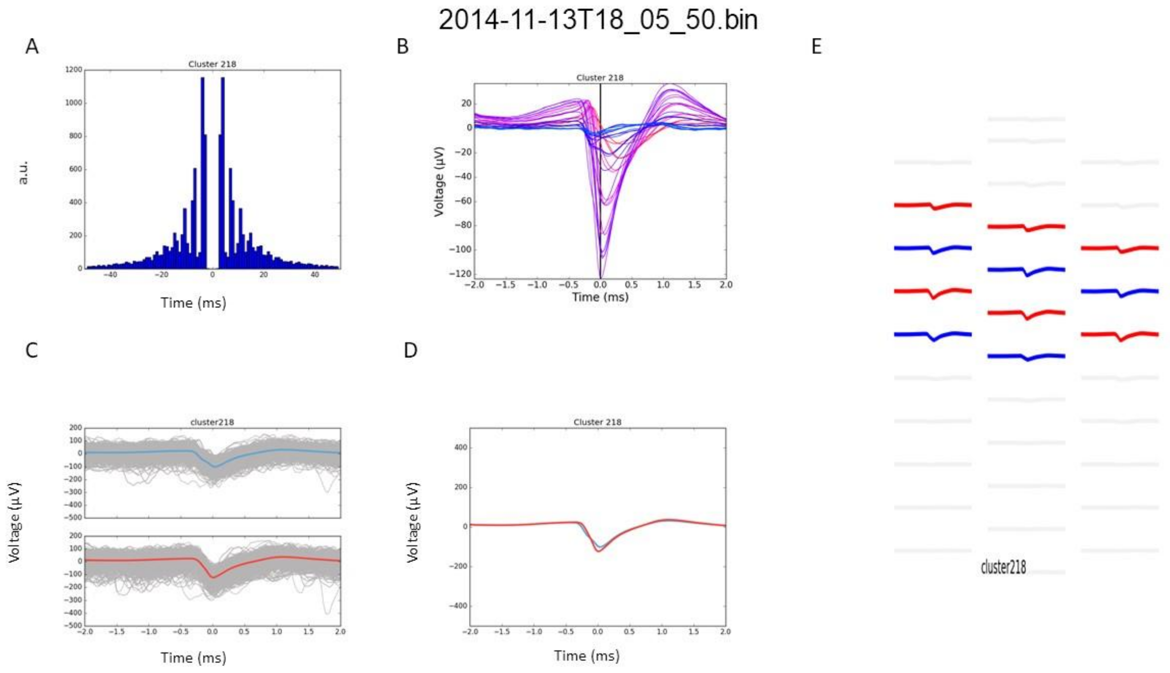
2014-11-13T18_05_50.bin
A
Cluster 218
a.u.
Time (ms)
B
Cluster 218
Voltage (μV)
Time (ms)
E
C
cluster218
Voltage (μV)
Time (ms)
D
Cluster 218
Voltage (μV)
Time (ms)
cluster218

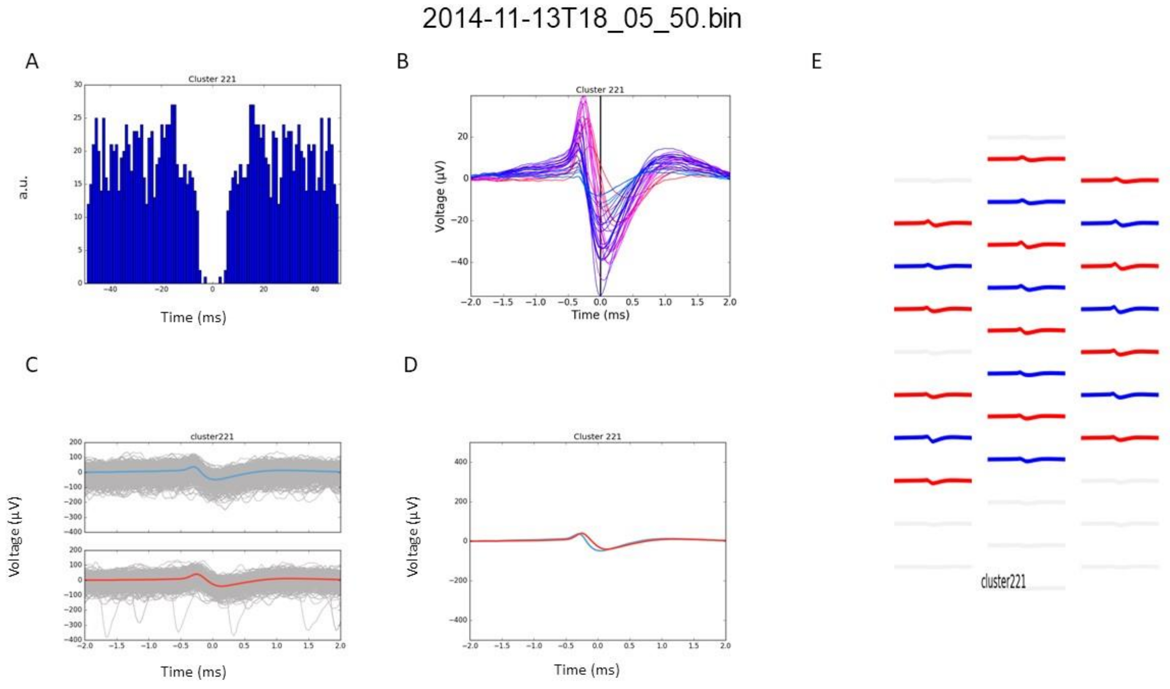
2014-11-13T18_05_50.bin
A
Cluster 221
a.u.
Time (ms)
B
Cluster 221
Voltage (μV)
Time (ms)
E
C
cluster221
Voltage (μV)
Time (ms)
D
Cluster 221
Voltage (μV)
Time (ms)
cluster221

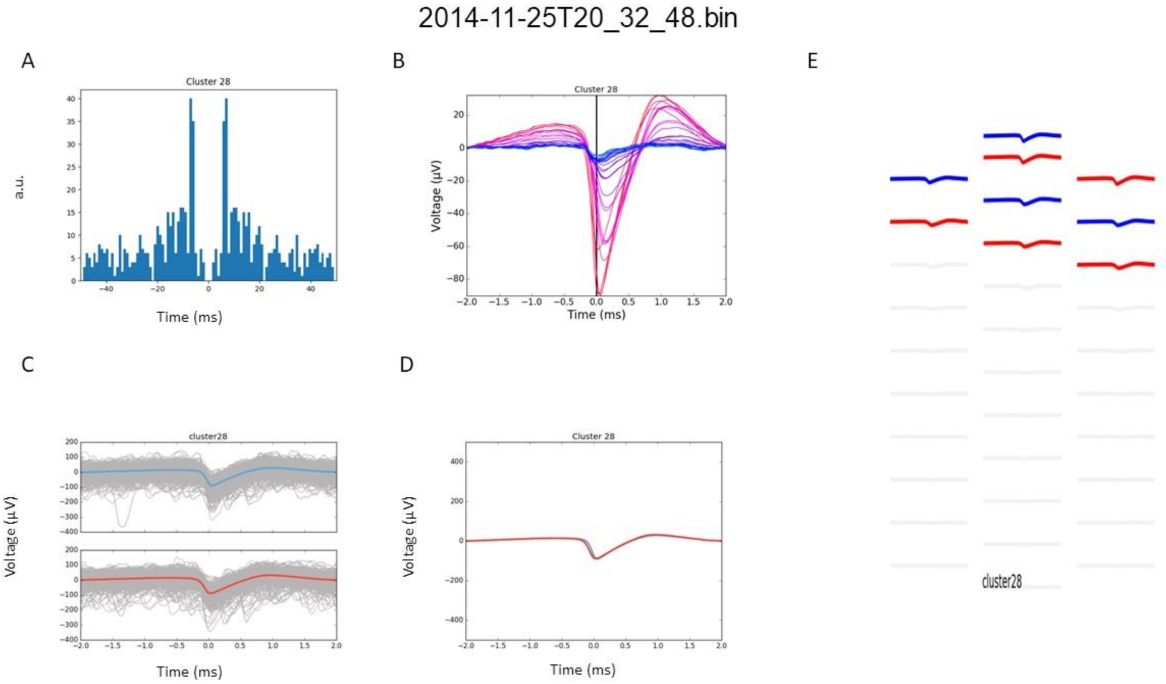
2014-11-25T20_32_48.bin
A
B
E
Cluster 28
a.u.
Time (ms)
Voltage (µV)
C
D
cluster28
cluster28

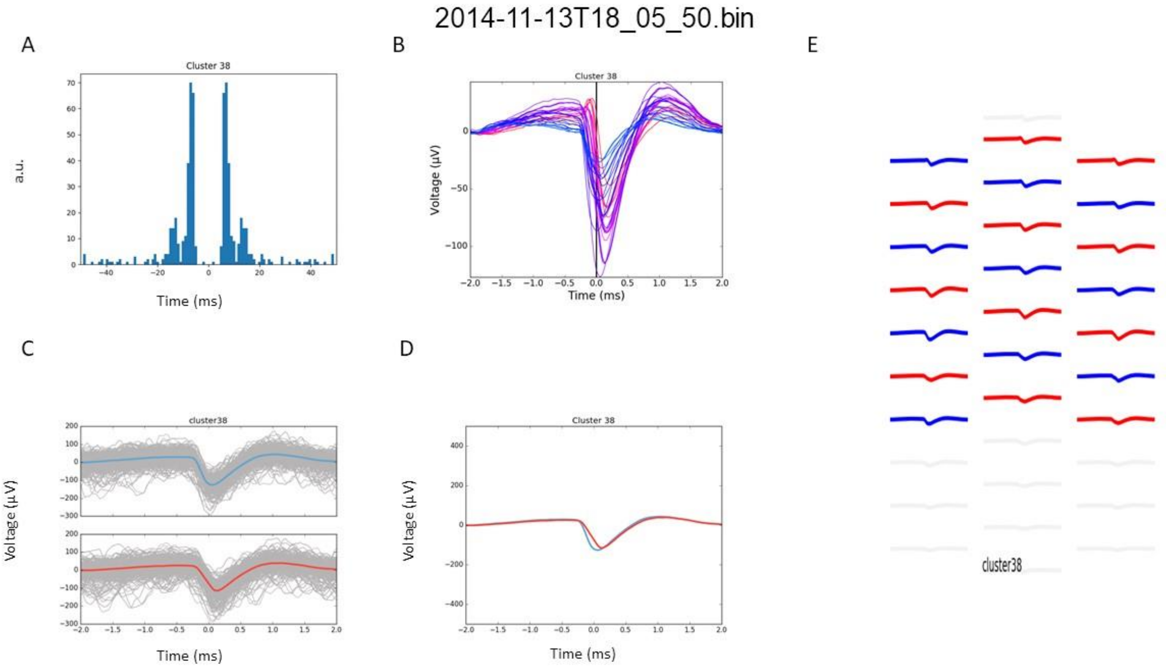
2014-11-13T18_05_50.bin
A
B
E
Cluster 38
a.u.
Time (ms)
Voltage (µV)
C
D
cluster38
cluster38

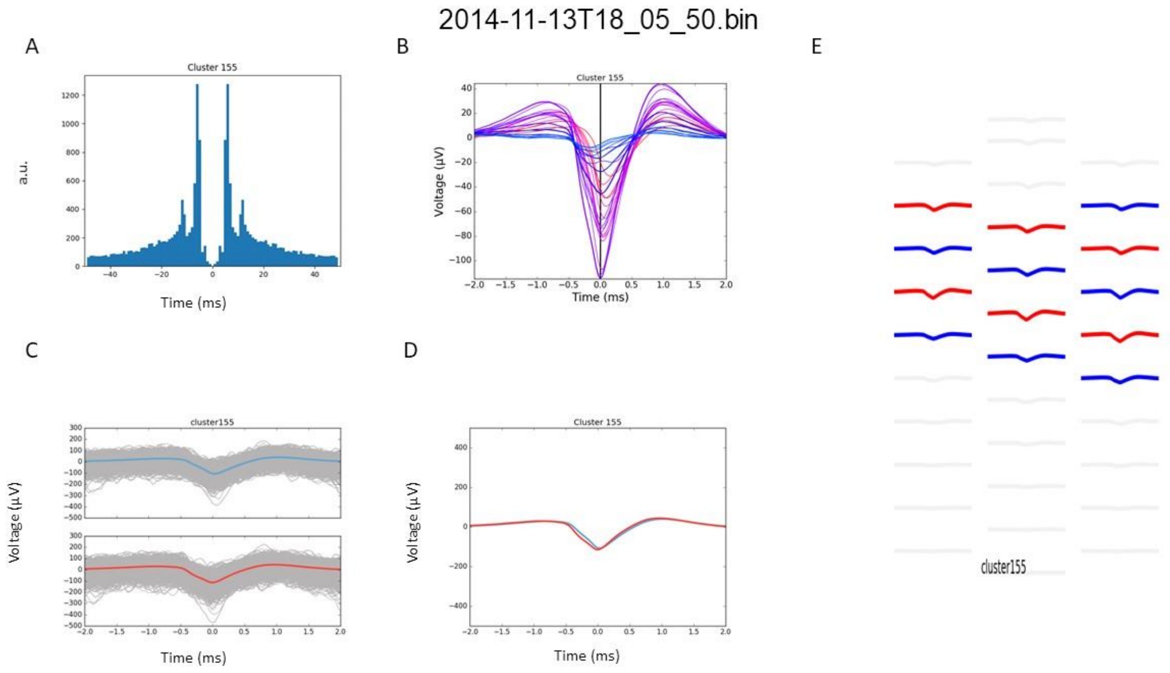
2014-11-13T18_05_50.bin
A
B
C
D
E
Cluster 155
a.u.
Time (ms)
Voltage (µV)
cluster155

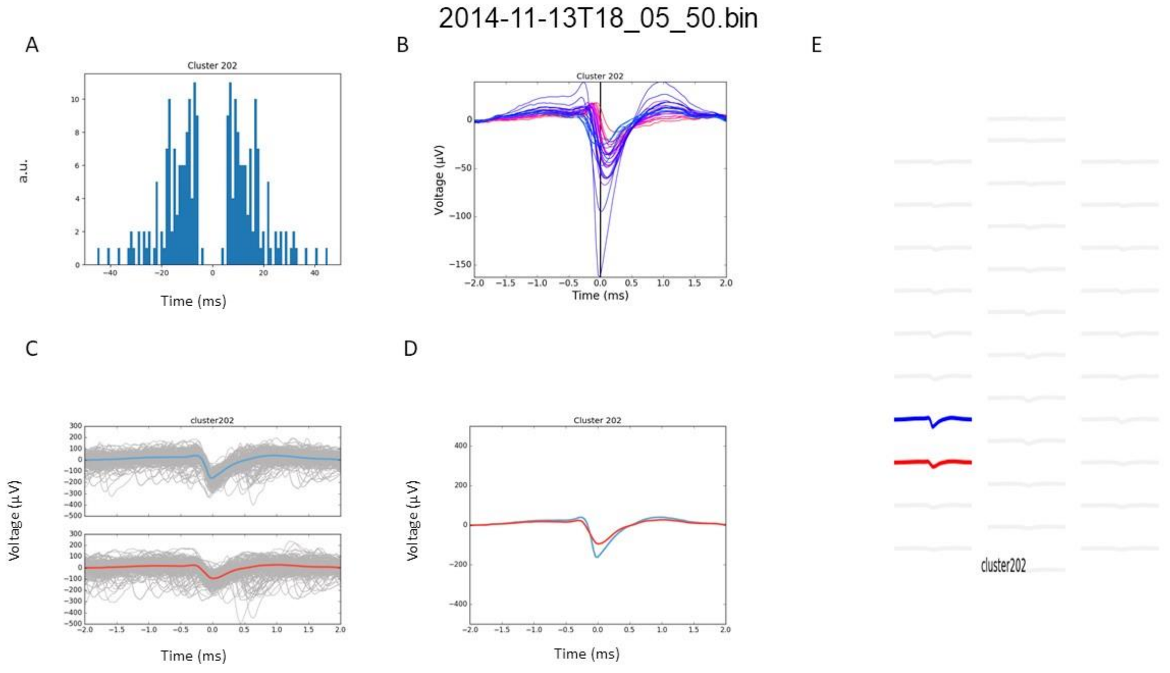
2014-11-13T18_05_50.bin
A
B
C
D
E
Cluster 202
a.u.
Time (ms)
Voltage (µV)
cluster202

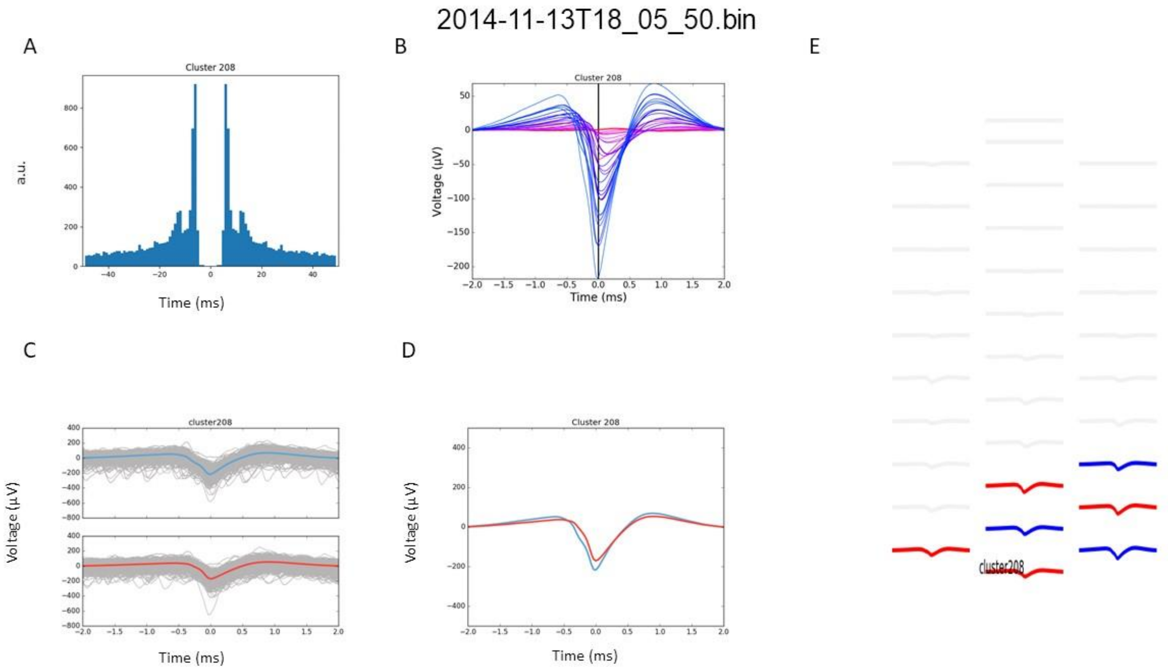
2014-11-13T18_05_50.bin
A
B
E
Cluster 208
a.u.
Time (ms)
Voltage (µV)
C
D
cluster208
Cluster 208
cluster208

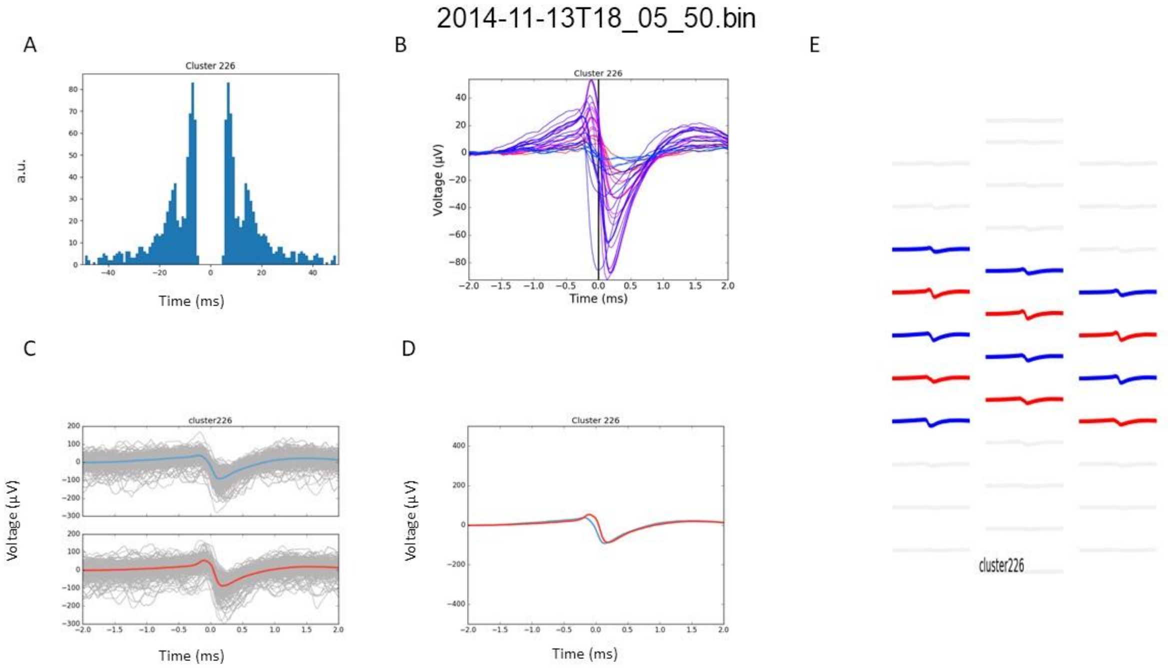
2014-11-13T18_05_50.bin
A
B
E
Cluster 226
a.u.
Time (ms)
Voltage (µV)
C
D
cluster226
Cluster 226
cluster226

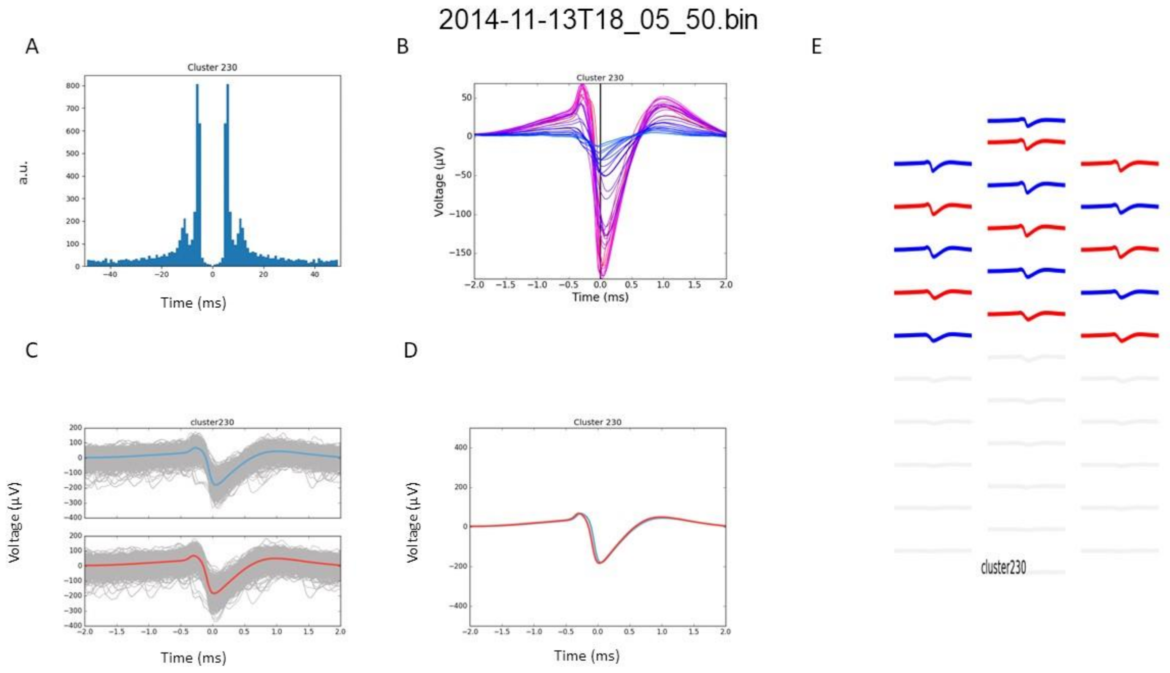
2014-11-13T18_05_50.bin
A
Cluster 230
a.u.
Time (ms)
B
Cluster 230
Voltage (µV)
Time (ms)
E
C
cluster230
Voltage (µV)
Time (ms)
D
Cluster 230
Voltage (µV)
Time (ms)
cluster230

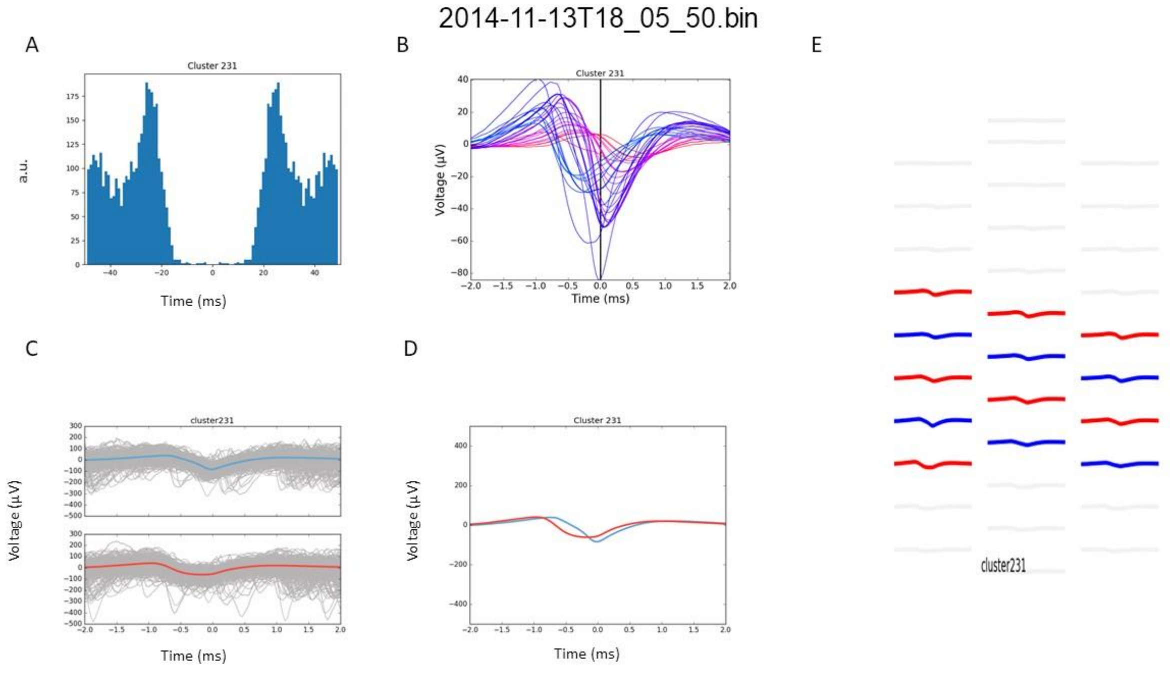
2014-11-13T18_05_50.bin
A
Cluster 231
a.u.
Time (ms)
B
Cluster 231
Voltage (µV)
Time (ms)
E
C
cluster231
Voltage (µV)
Time (ms)
D
Cluster 231
Voltage (µV)
Time (ms)
cluster231

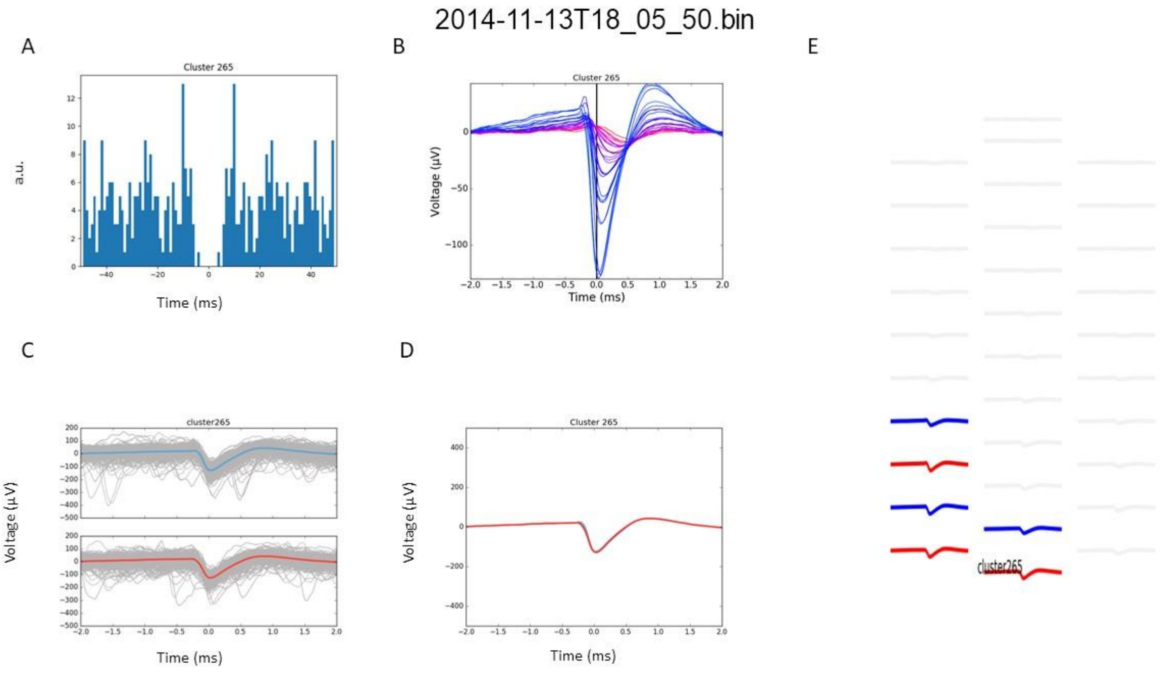
2014-11-13T18_05_50.bin
A
B
E
Cluster 265
a.u.
Time (ms)
Voltage (µV)
C
D
cluster265
Cluster 265
cluster265

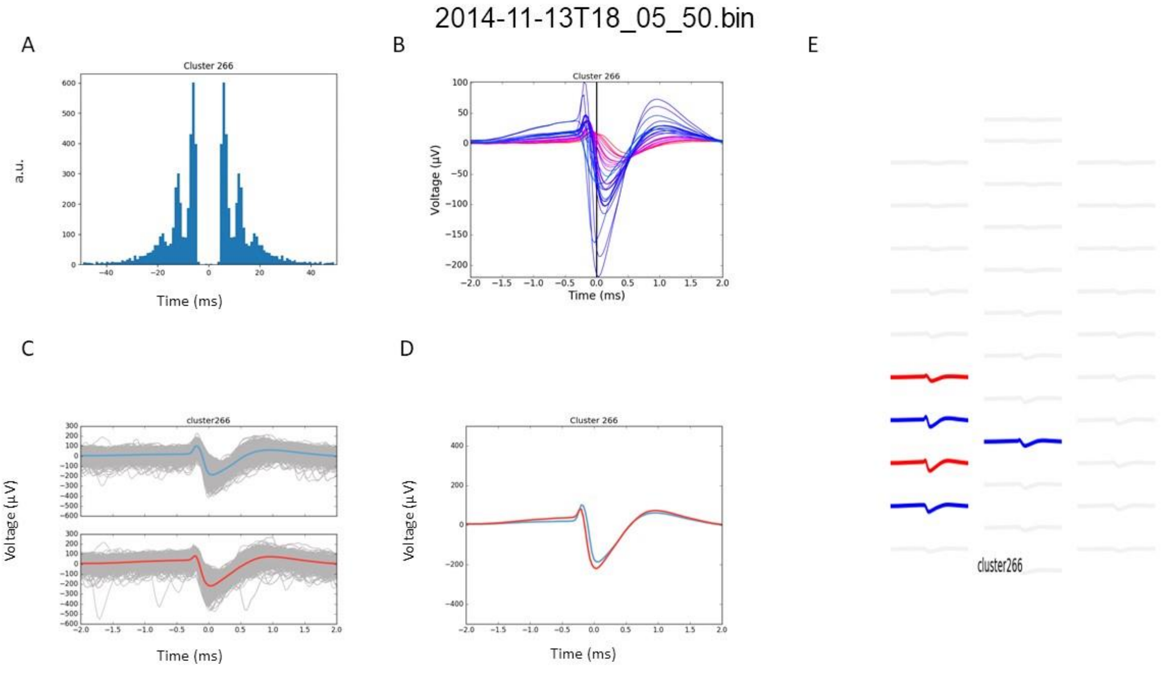
2014-11-13T18_05_50.bin
A
B
E
Cluster 266
a.u.
Time (ms)
Voltage (µV)
C
D
cluster266
Cluster 266
cluster266

2014-11-13T18_05_50.bin

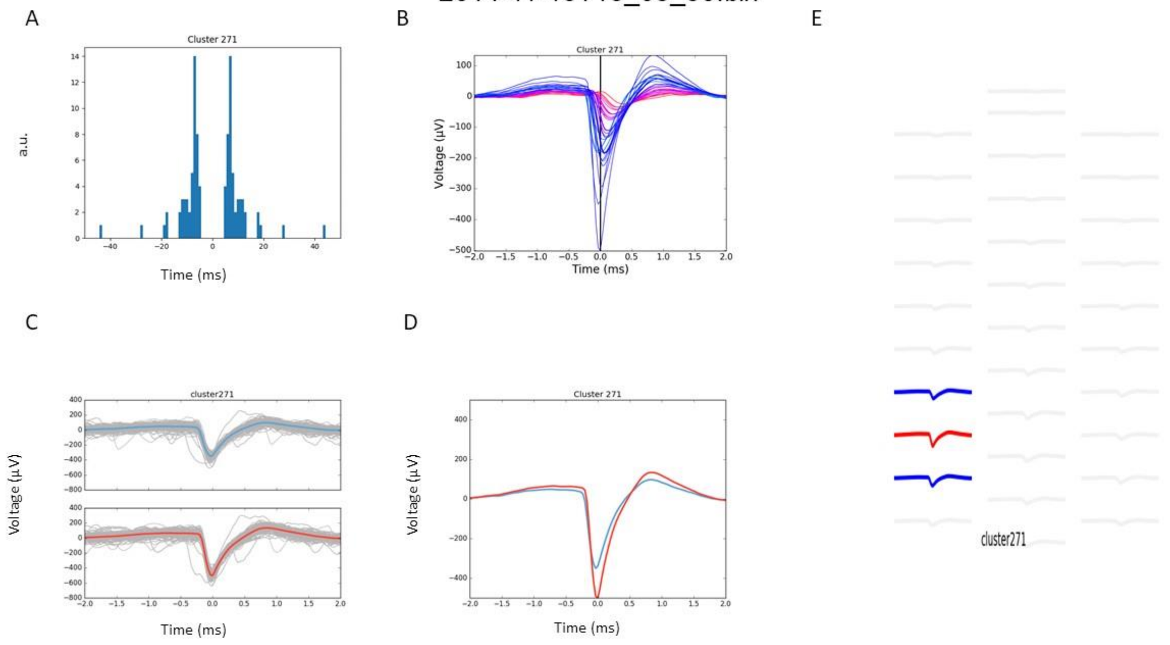

2014-11-25T21_27_13.bin

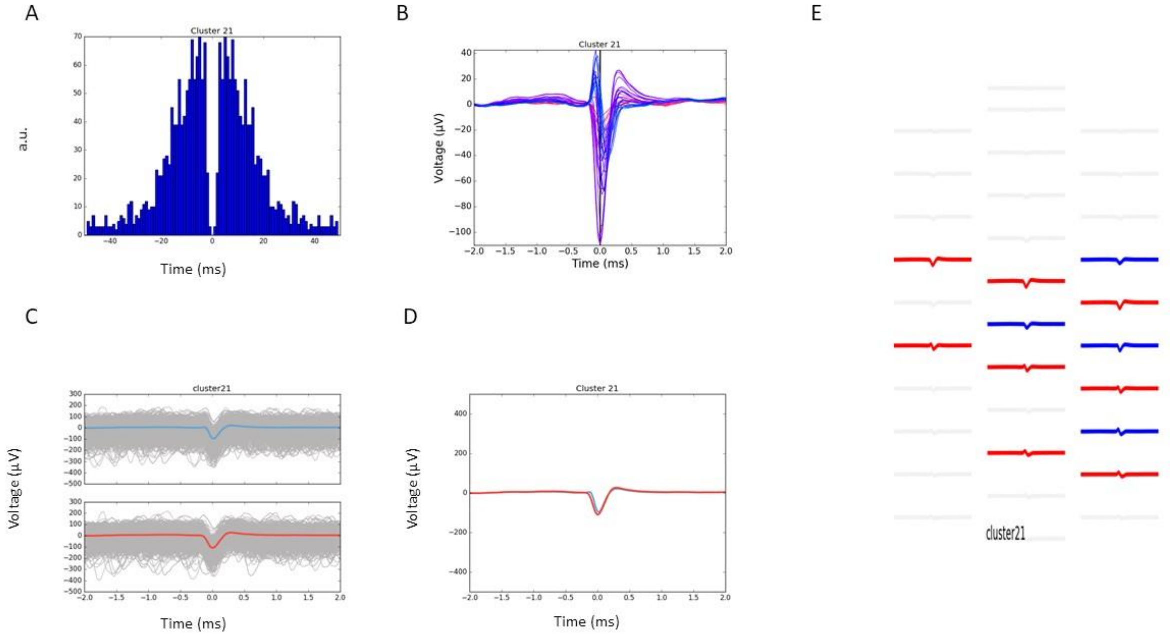

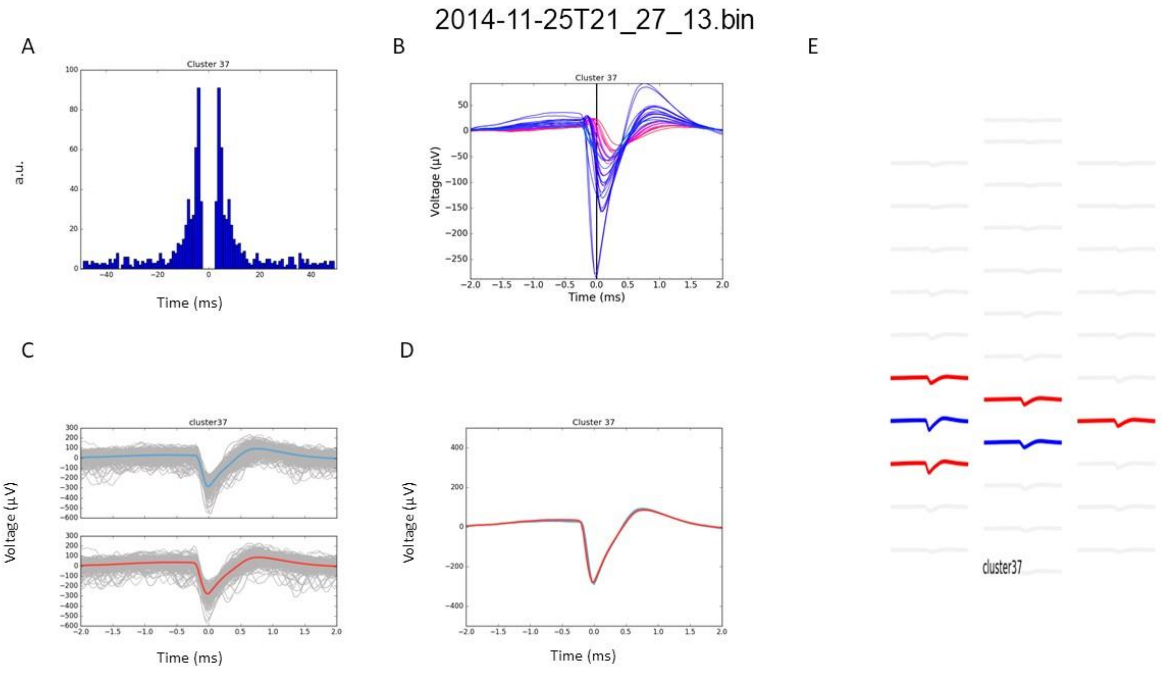
2014-11-25T21_27_13.bin
A
Cluster 37
a.u.
Time (ms)
B
Cluster 37
Voltage (µV)
Time (ms)
E
C
cluster37
Voltage (µV)
Time (ms)
D
Cluster 37
Voltage (µV)
Time (ms)
cluster37

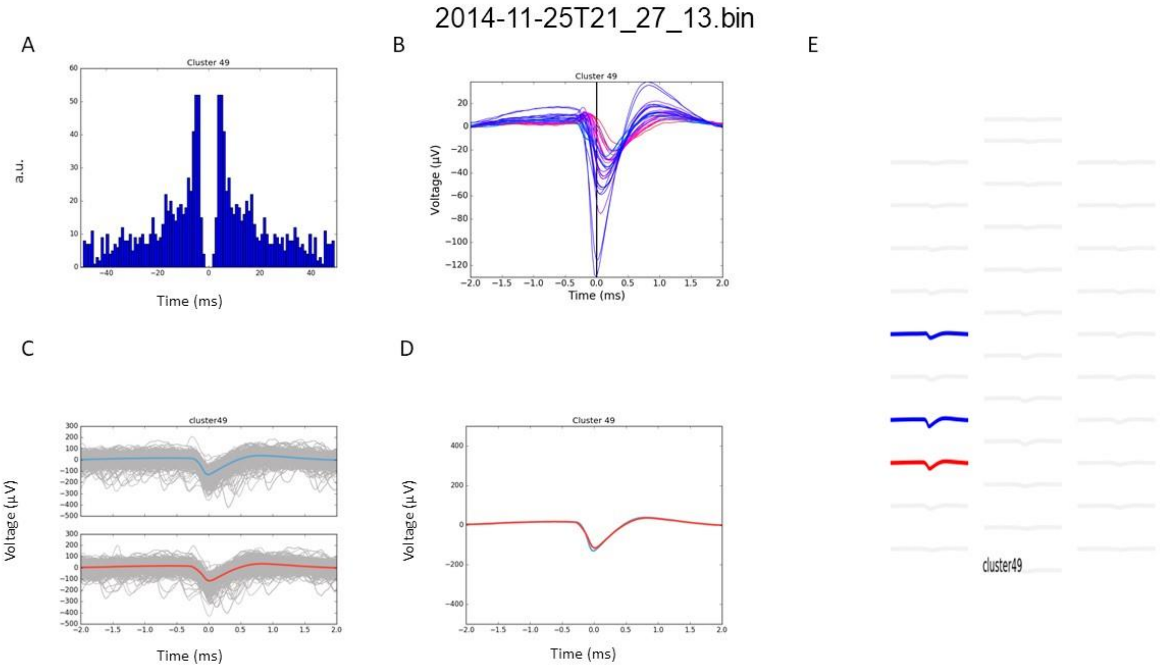
2014-11-25T21_27_13.bin
A
Cluster 49
a.u.
Time (ms)
B
Cluster 49
Voltage (µV)
Time (ms)
E
C
cluster49
Voltage (µV)
Time (ms)
D
Cluster 49
Voltage (µV)
Time (ms)
cluster49

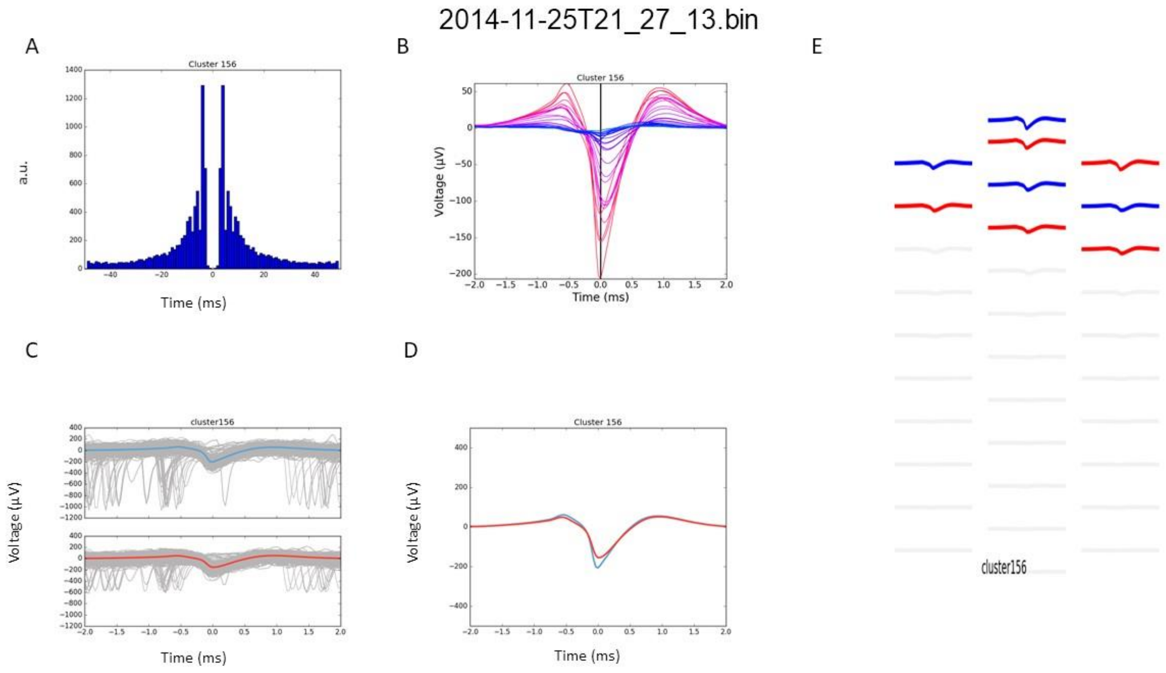
2014-11-25T21_27_13.bin
A
Cluster 156
a.u.
Time (ms)
B
Cluster 156
Voltage (µV)
Time (ms)
C
cluster156
Voltage (µV)
Time (ms)
D
Cluster 156
Voltage (µV)
Time (ms)
E
cluster156

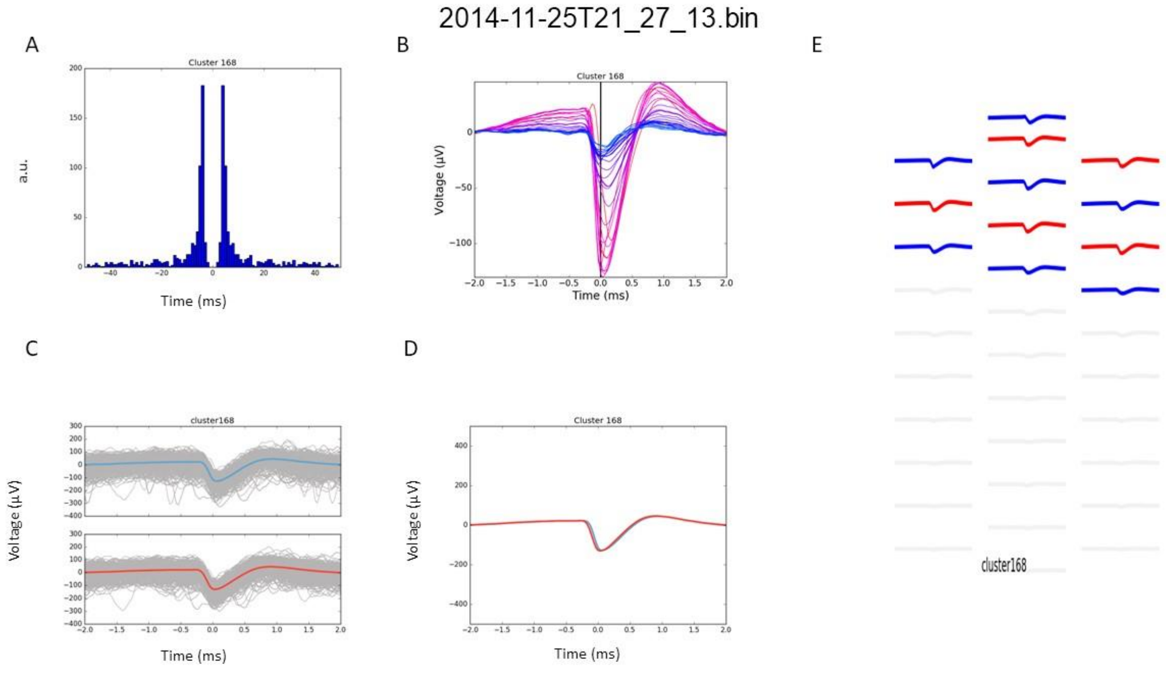
2014-11-25T21_27_13.bin
A
Cluster 168
a.u.
Time (ms)
B
Cluster 168
Voltage (µV)
Time (ms)
C
cluster168
Voltage (µV)
Time (ms)
D
Cluster 168
Voltage (µV)
Time (ms)
E
cluster168

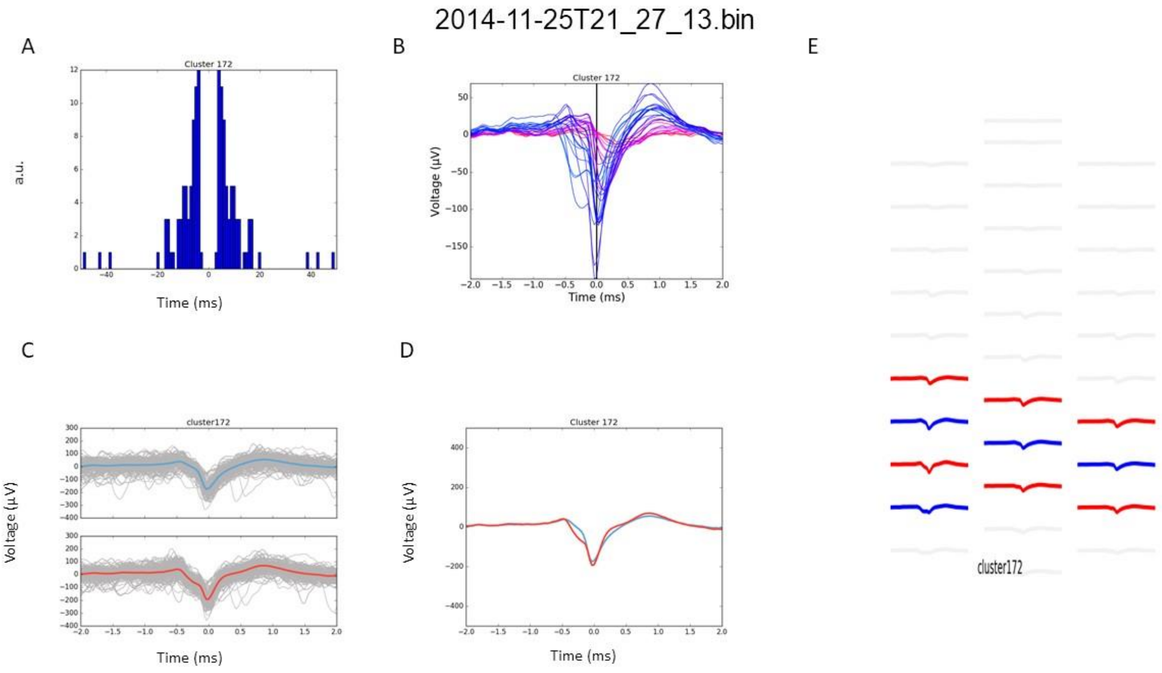
2014-11-25T21_27_13.bin
A
B
E
Cluster 172
a.u.
Time (ms)
Voltage (µV)
C
D
cluster172
Cluster 172
cluster172

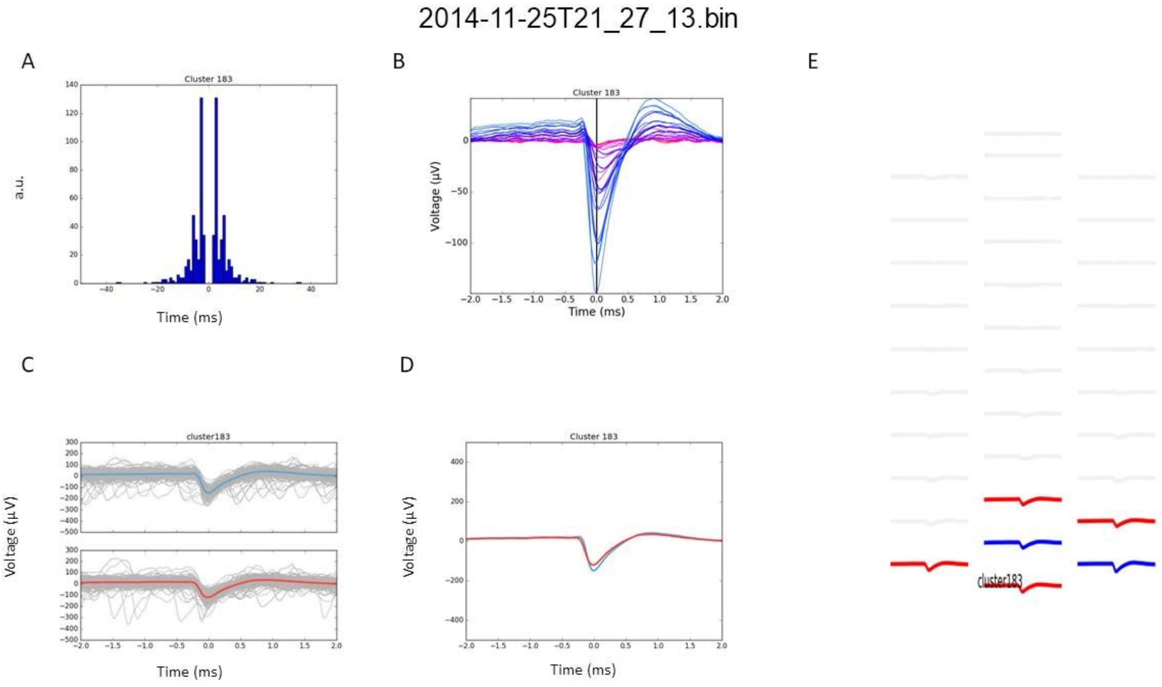
2014-11-25T21_27_13.bin
A
B
E
Cluster 183
a.u.
Time (ms)
Voltage (µV)
C
D
cluster183
Cluster 183
cluster183

2014-11-25T23_00_08.bin

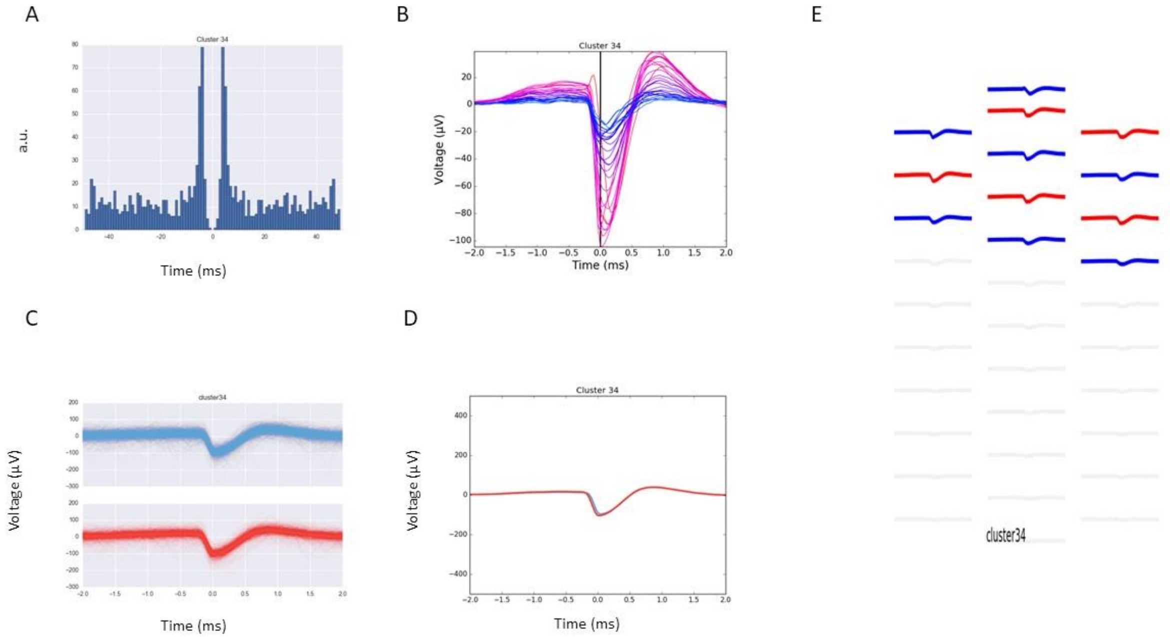

2014-11-25T23_00_08.bin

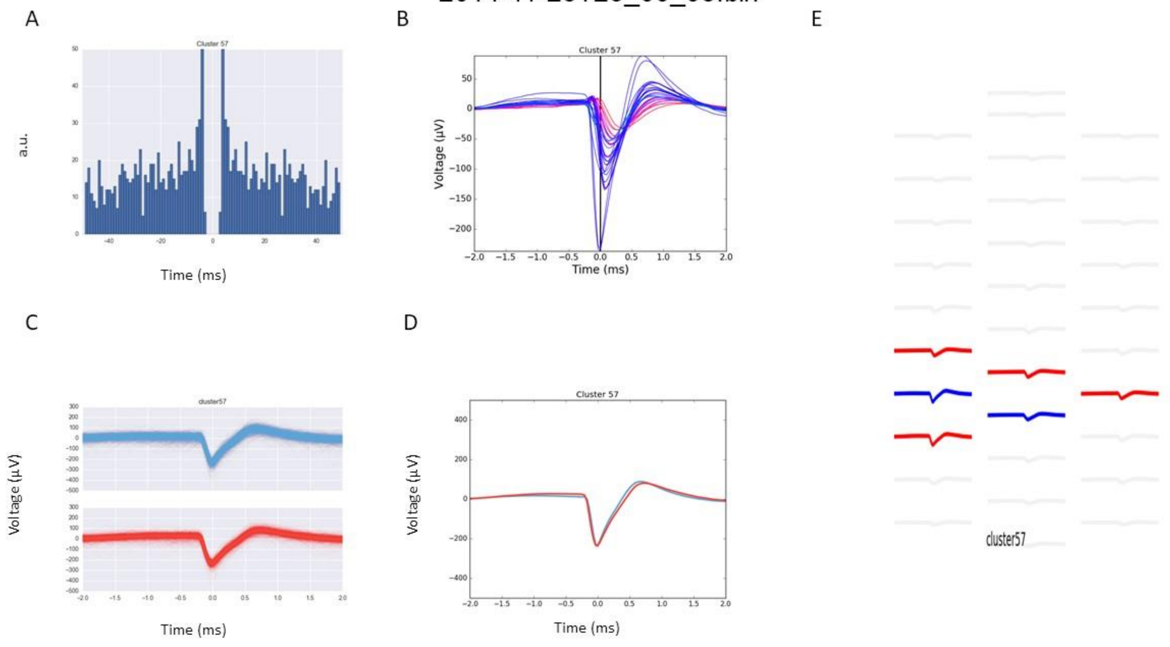

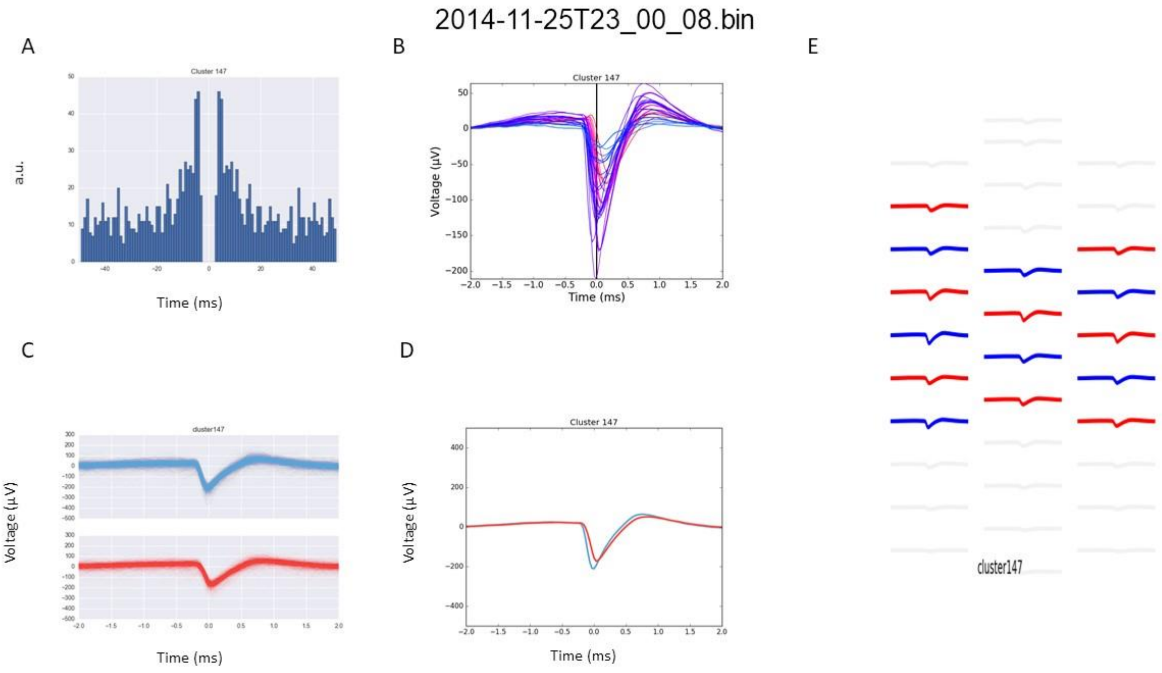
2014-11-25T23_00_08.bin
A
B
E
Cluster 147
a.u.
Time (ms)
Voltage (µV)
C
D
cluster147
Voltage (µV)
Time (ms)
Cluster 147
Voltage (µV)
Time (ms)
cluster147

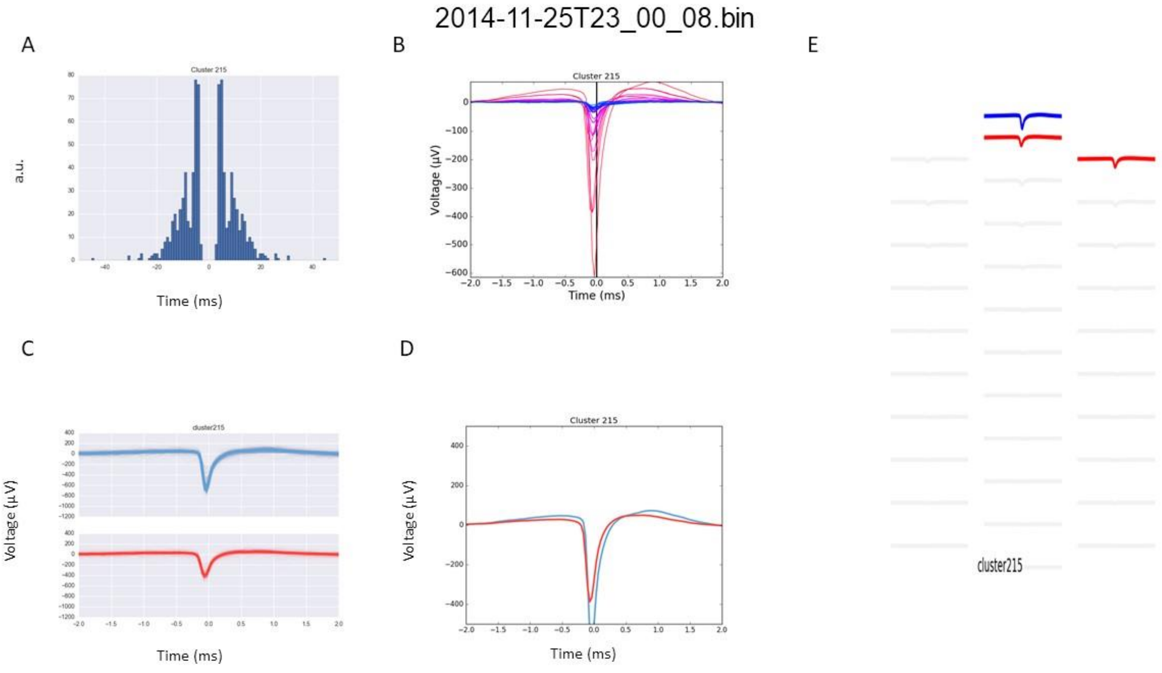
2014-11-25T23_00_08.bin
A
B
E
Cluster 215
a.u.
Time (ms)
Voltage (µV)
C
D
cluster215
Voltage (µV)
Time (ms)
Cluster 215
Voltage (µV)
Time (ms)
cluster215

2014-11-25T23_00_08.bin

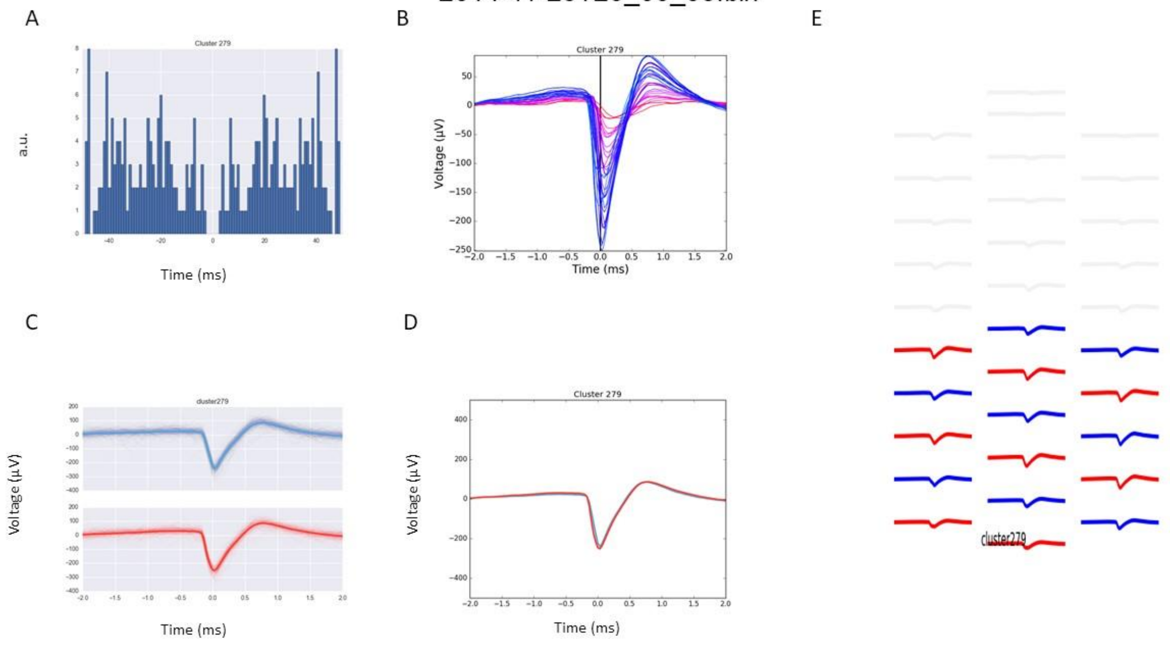

2014-11-25T23_00_08.bin

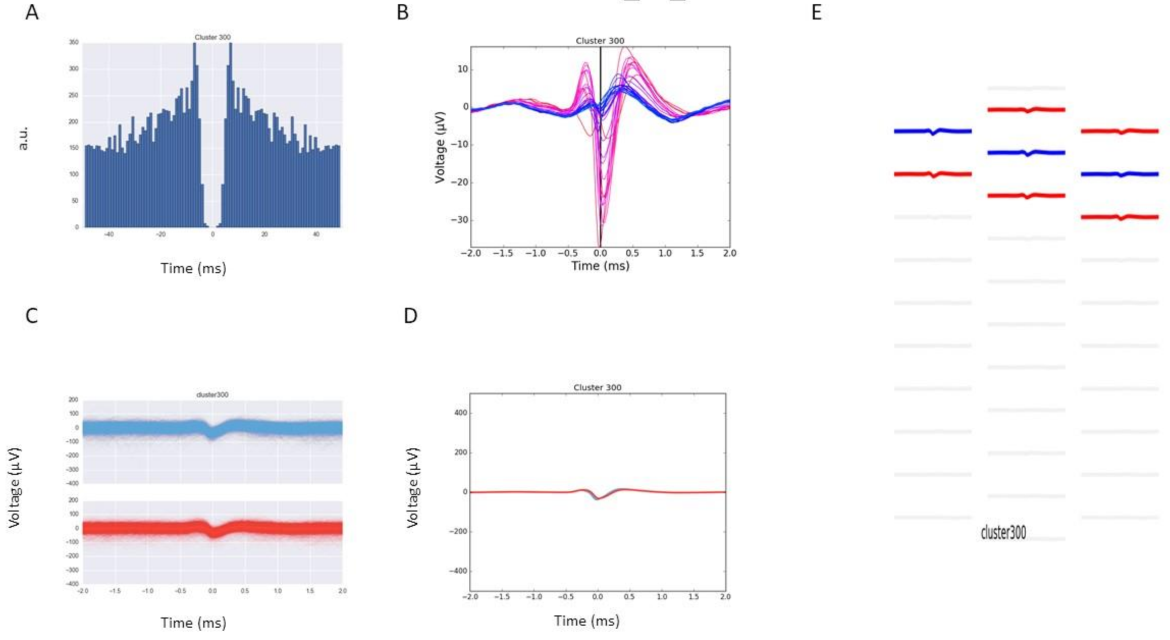

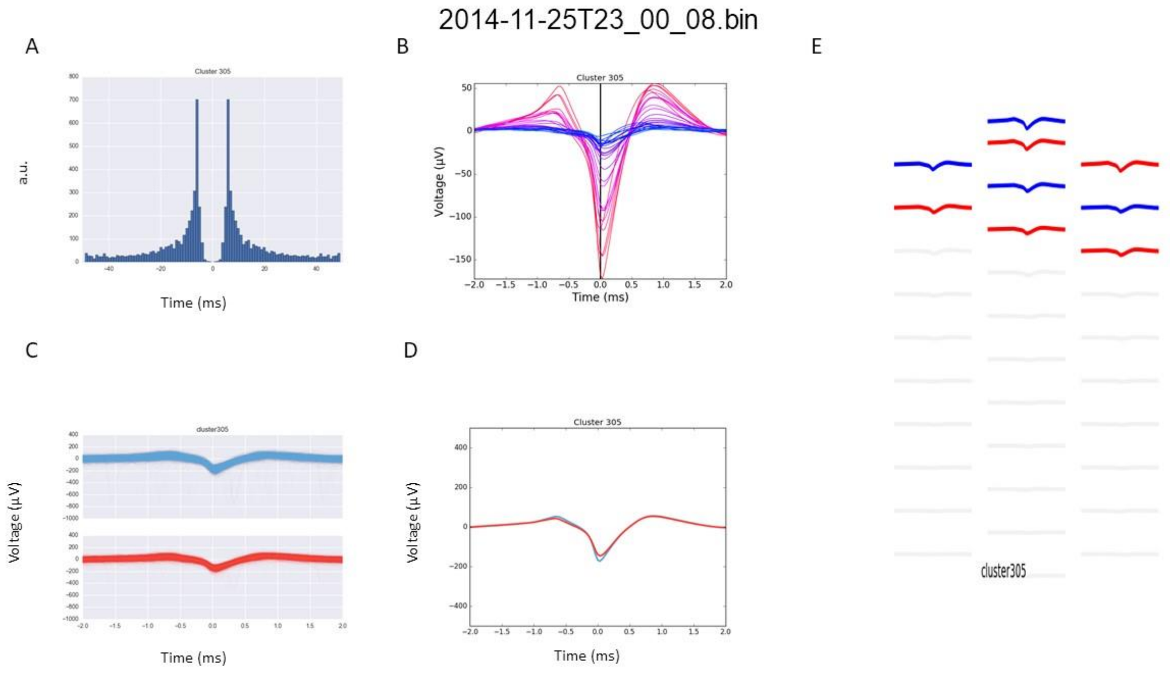
2014-11-25T23_00_08.bin
A
B
E
Cluster 305
a.u.
Time (ms)
Voltage (µV)
C
D
cluster305
Cluster 305
cluster305

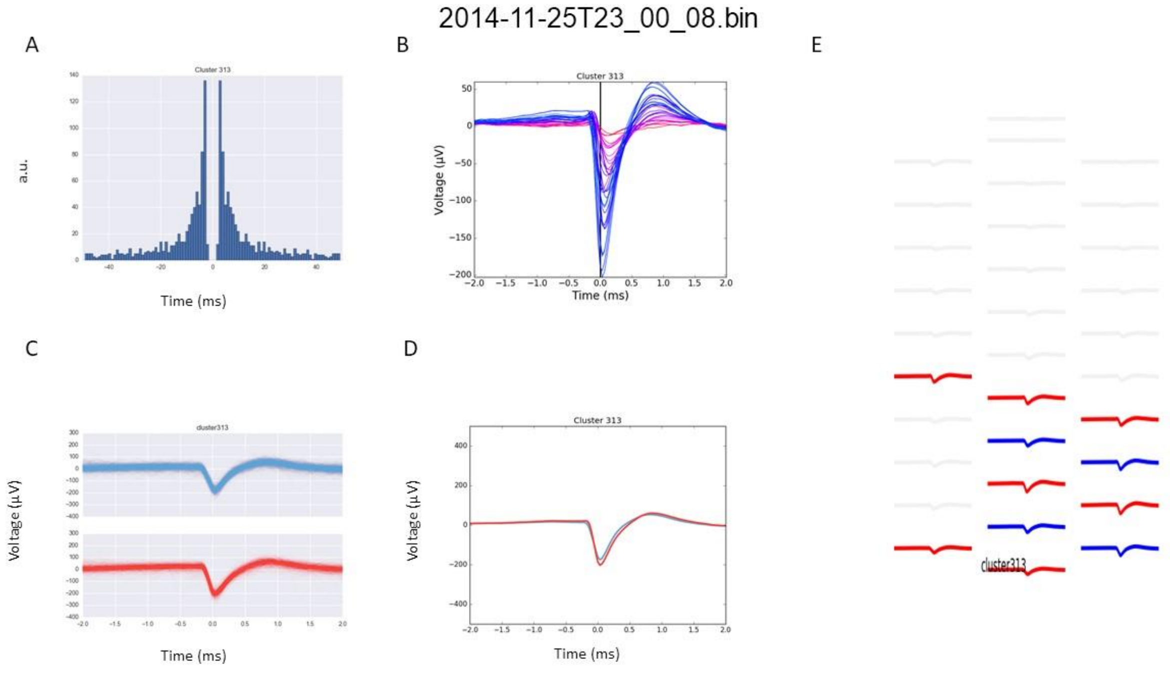
2014-11-25T23_00_08.bin
A
B
E
Cluster 313
a.u.
Time (ms)
Voltage (µV)
C
D
cluster313
Cluster 313
cluster313

2014-11-25T23_00_08.bin

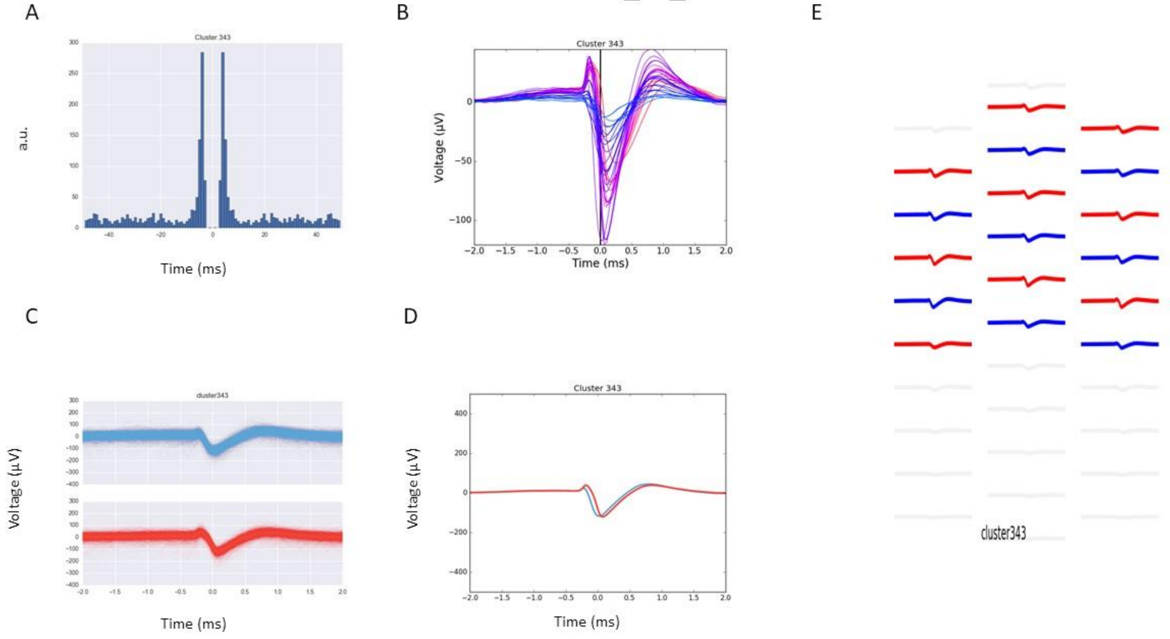

2014-11-25T23_00_08.bin

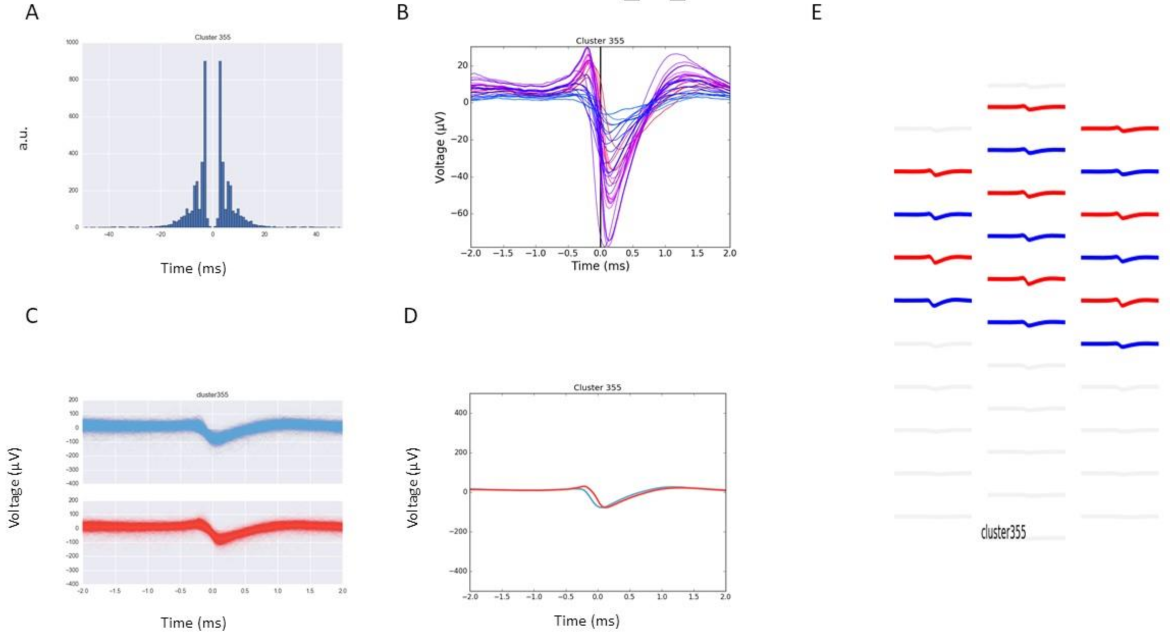

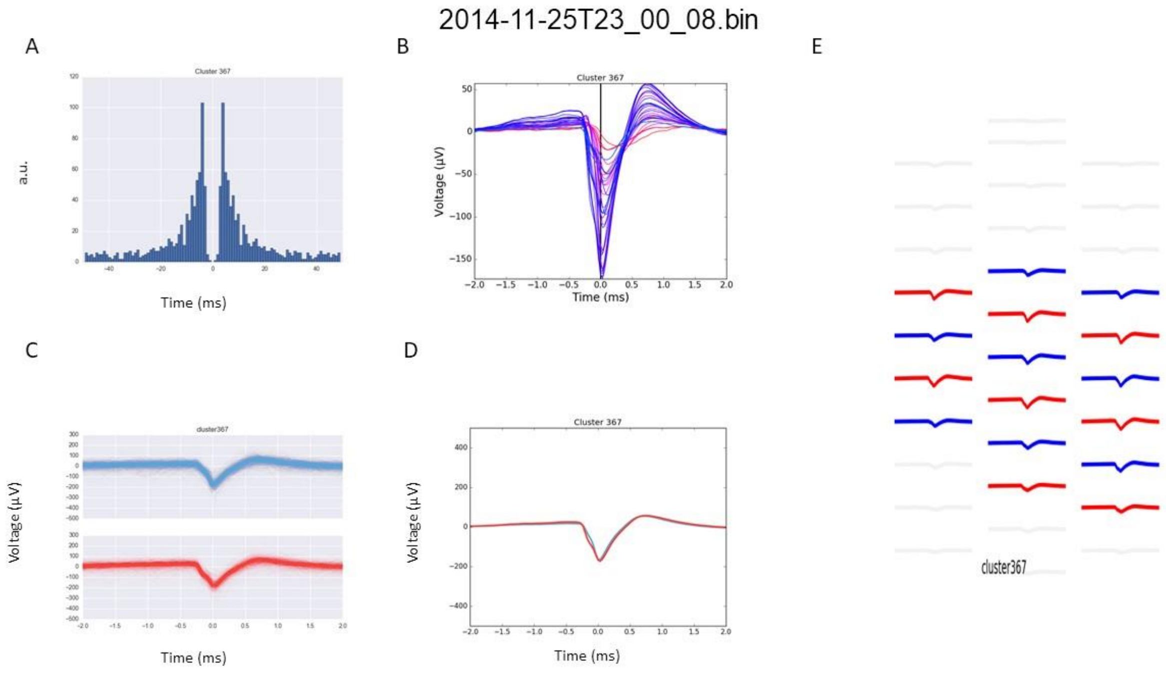
2014-11-25T23_00_08.bin
A
B
E
Cluster 367
a.u.
Time (ms)
Voltage (μV)
C
D
cluster367
Cluster 367
Voltage (μV)
Time (ms)
cluster367

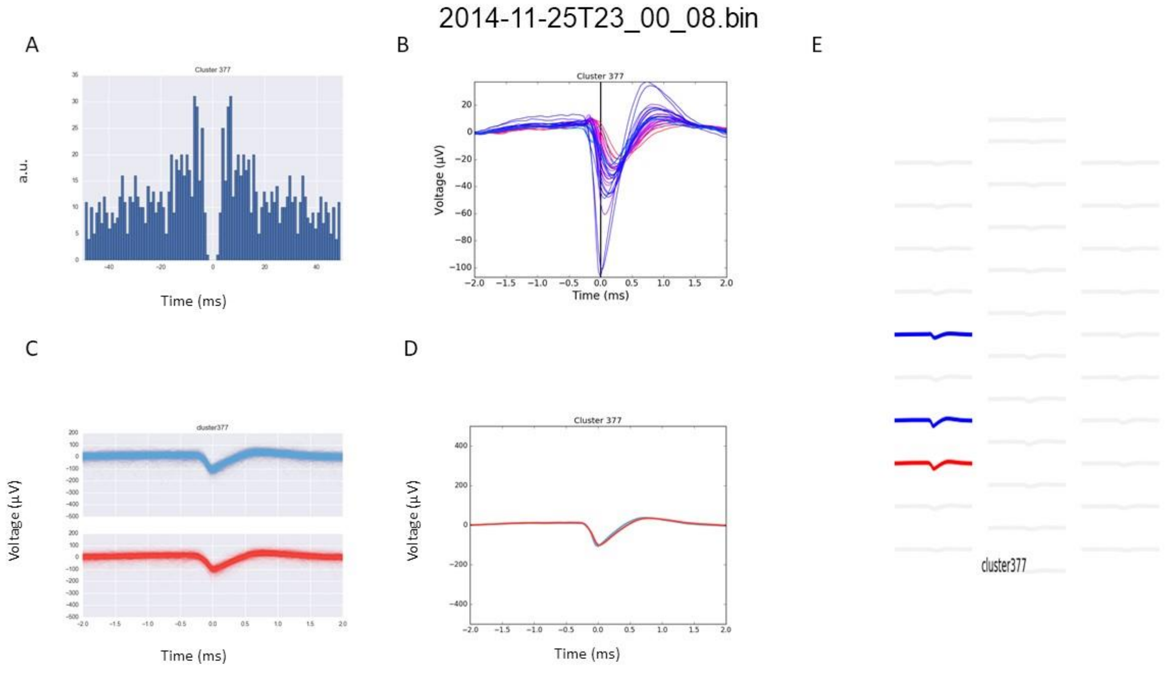
2014-11-25T23_00_08.bin
A
B
E
Cluster 377
a.u.
Time (ms)
Voltage (μV)
C
D
cluster377
Cluster 377
Voltage (μV)
Time (ms)
cluster377

2017-02-02T14_38_11.bin

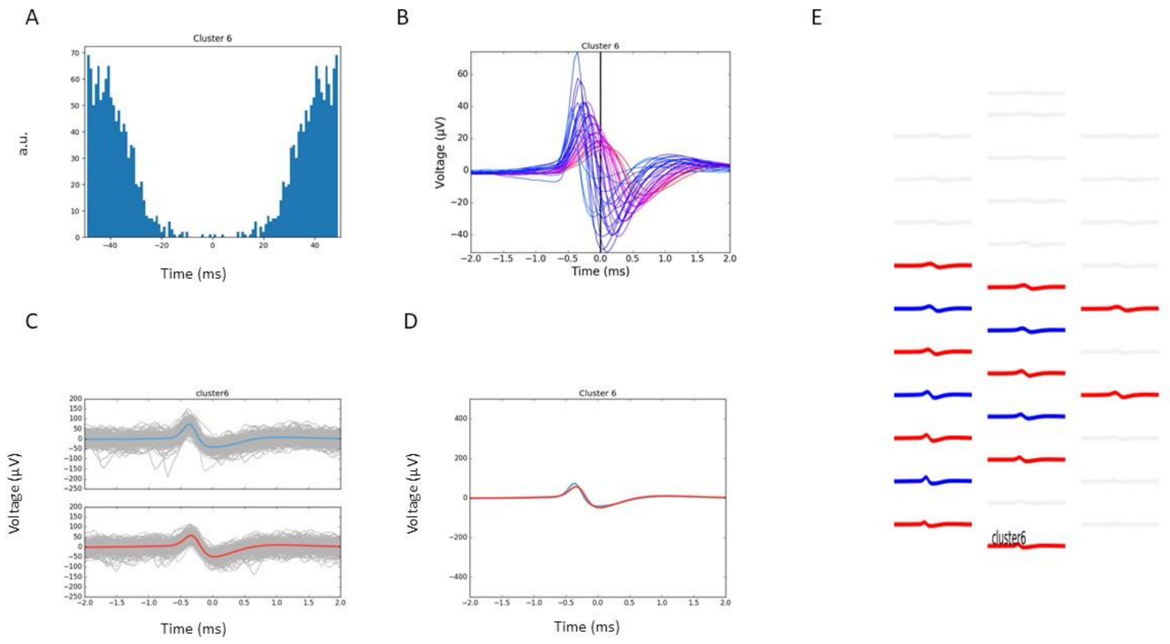

2017-02-02T15_03_44.bin

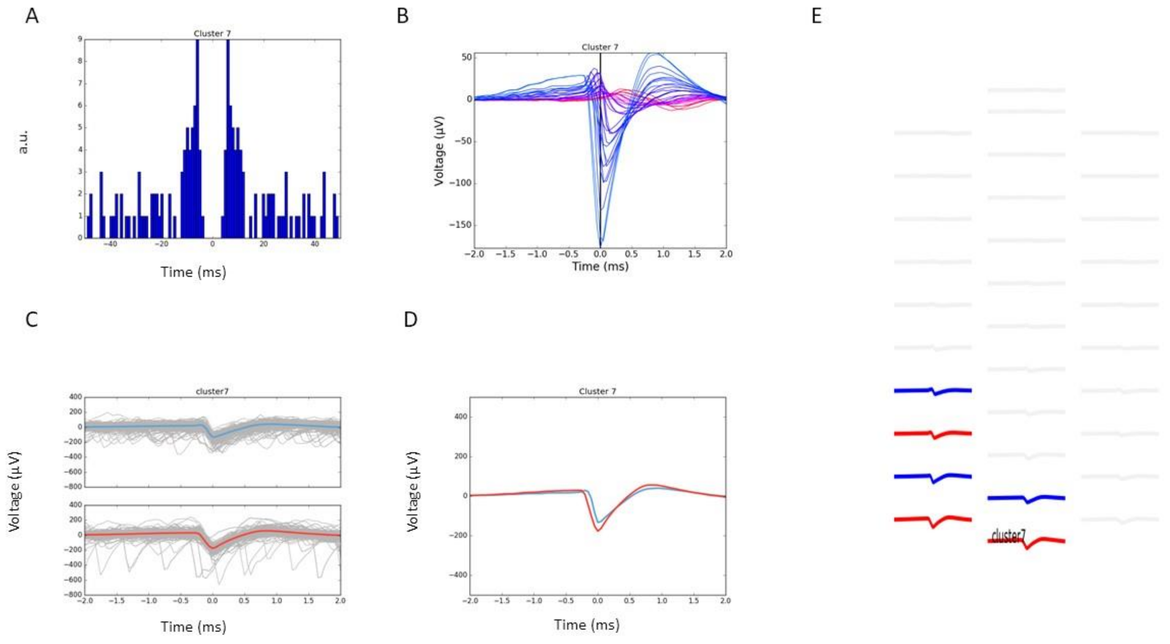

2017-02-02T15_03_44.bin

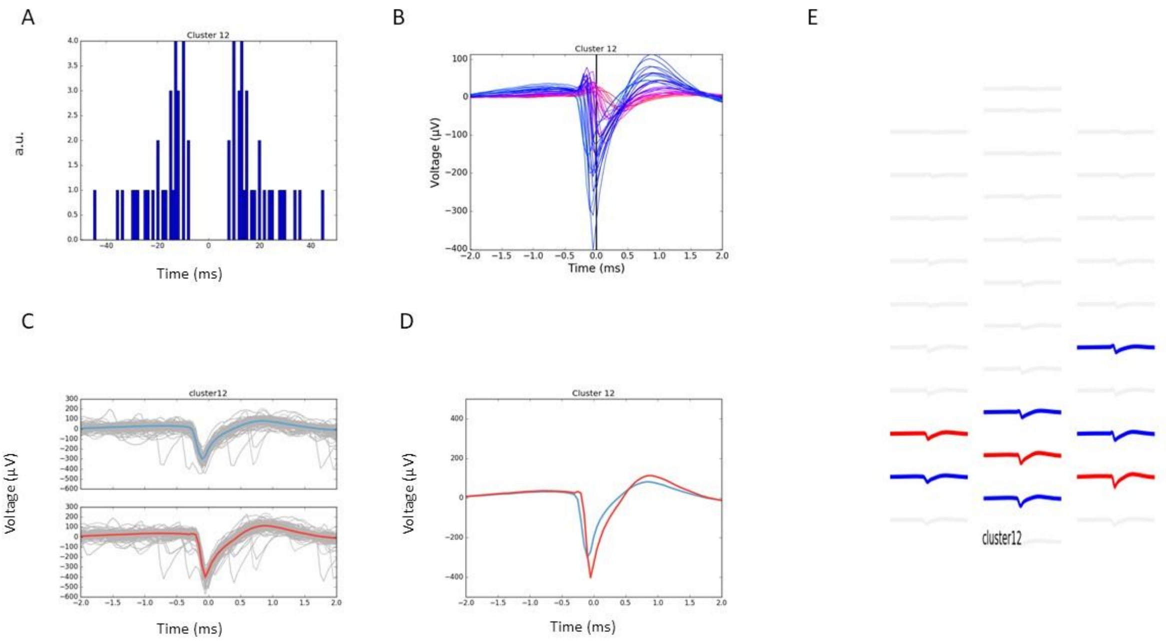

2017-02-02T15_03_44.bin

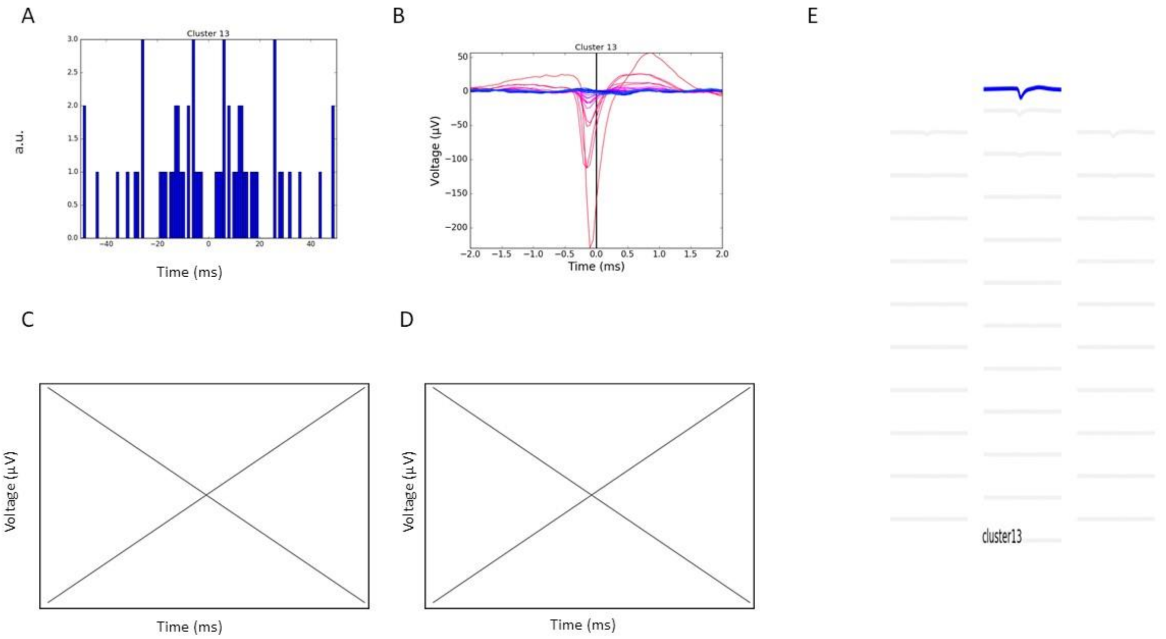

2017-02-02T15_03_44.bin

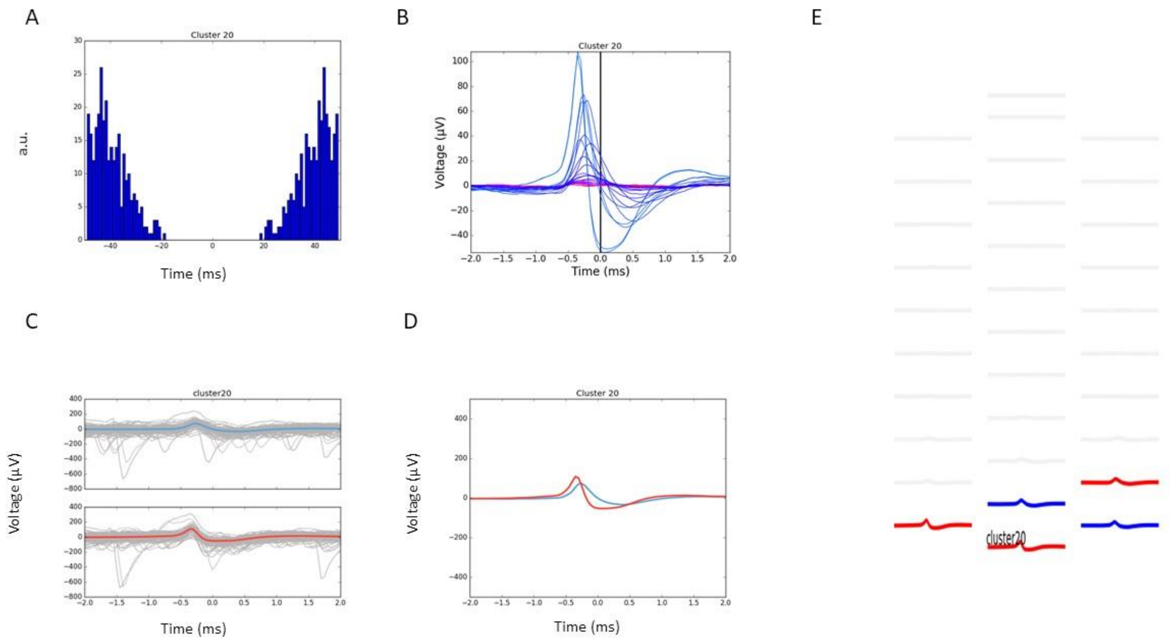

2017-02-02T15_03_44.bin

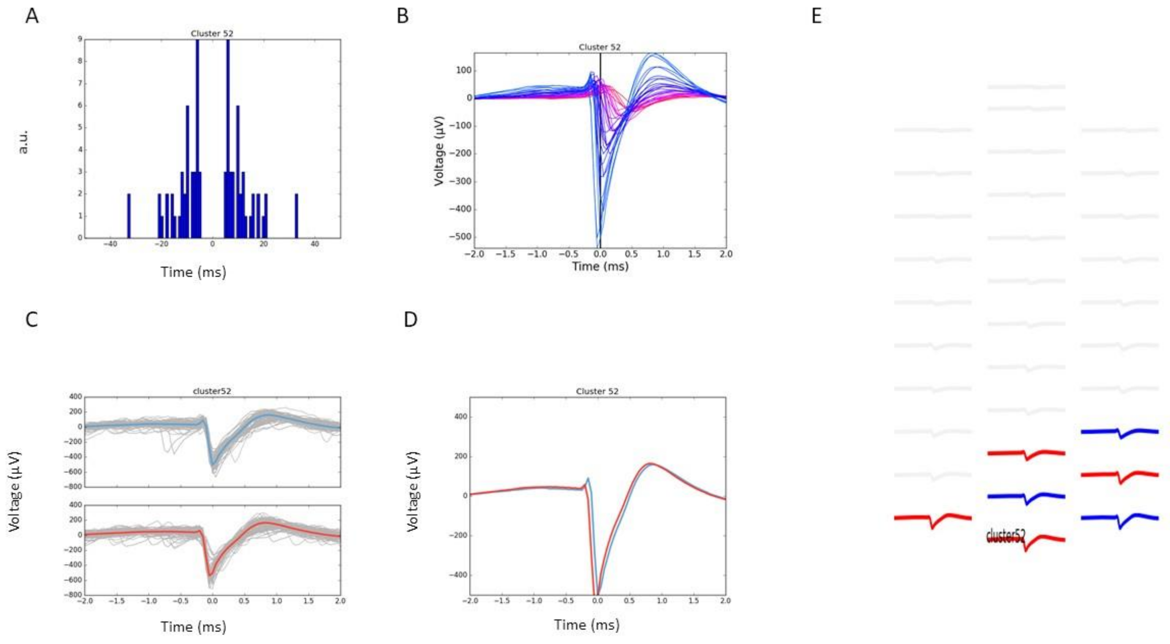

2017-02-02T15_03_44.bin

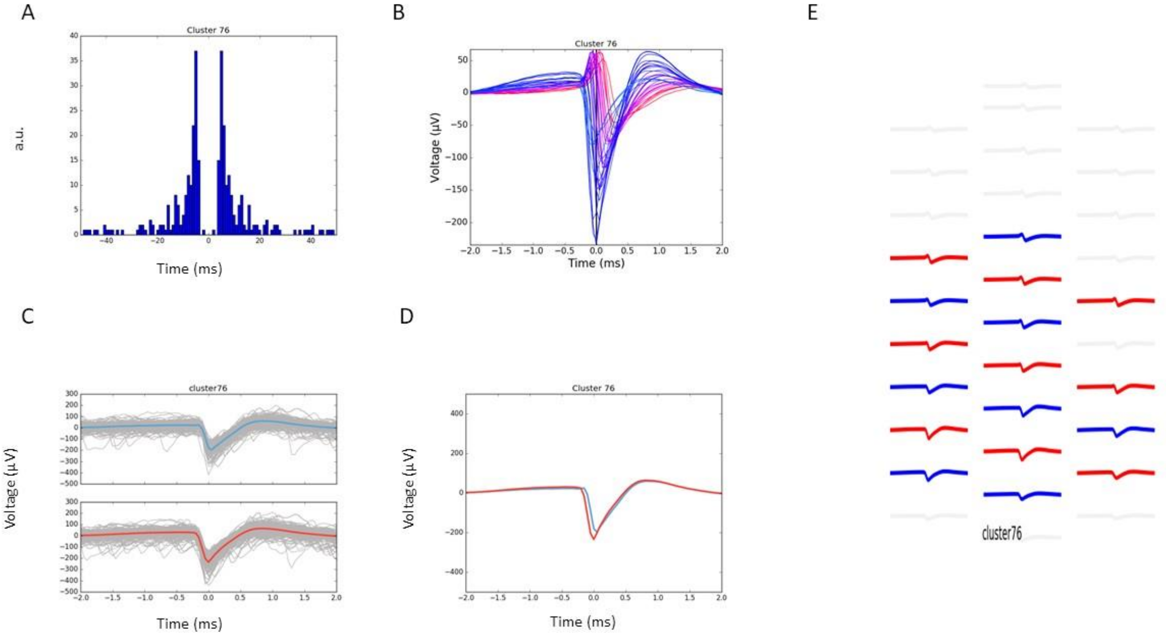

2017-02-02T15_49_35.bin

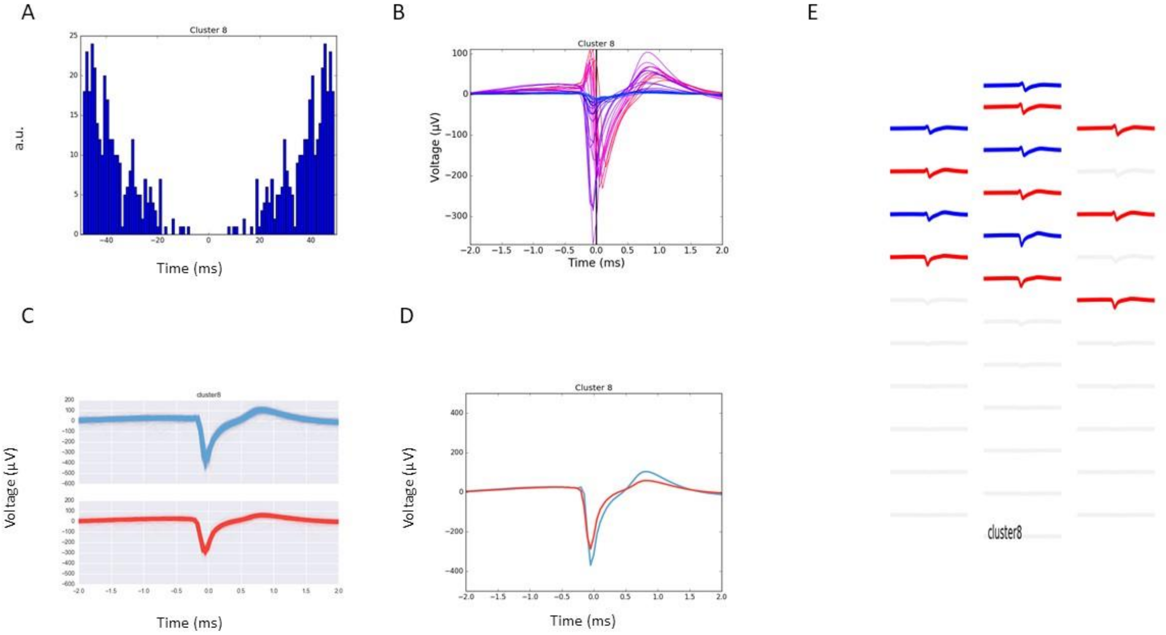

2017-02-02T15_49_35.bin

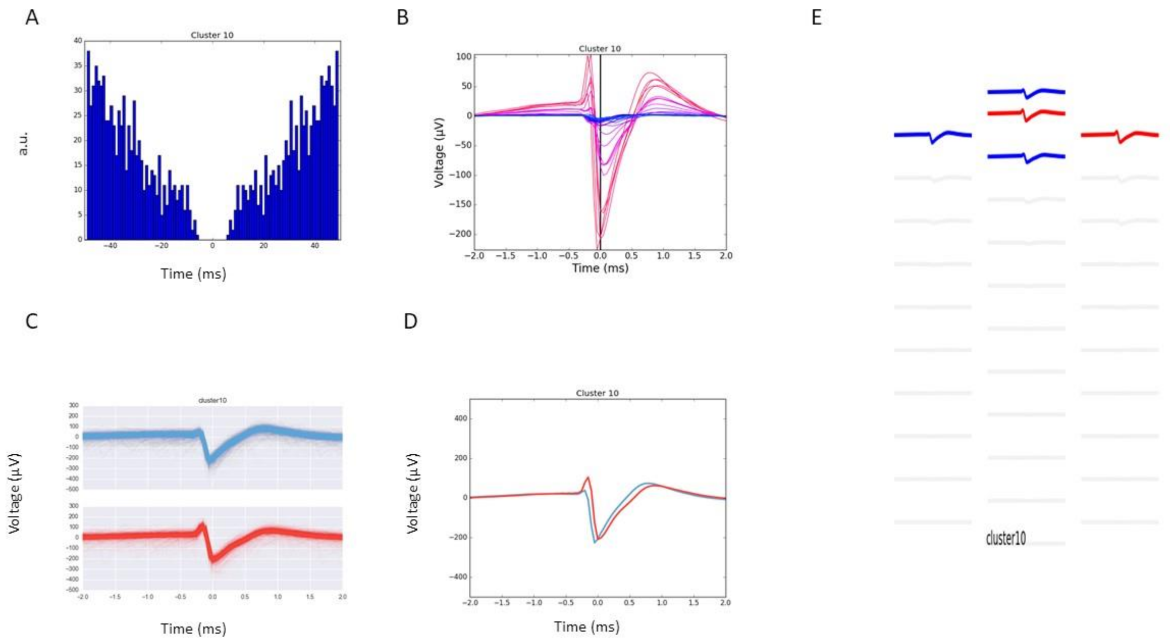

2017-02-02T15_49_35.bin

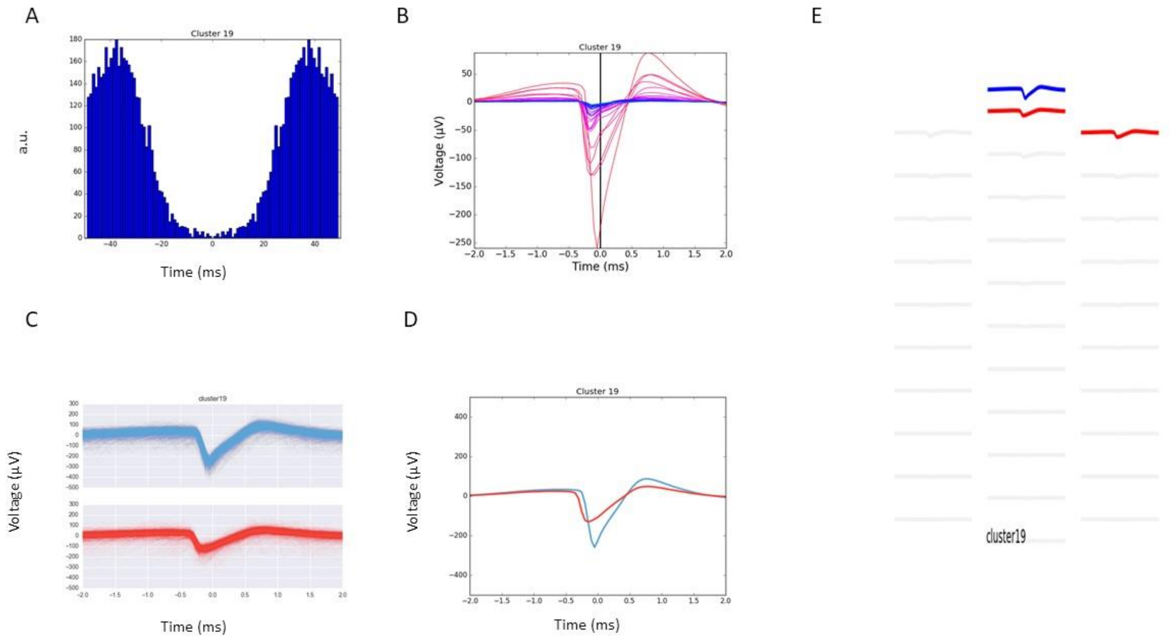

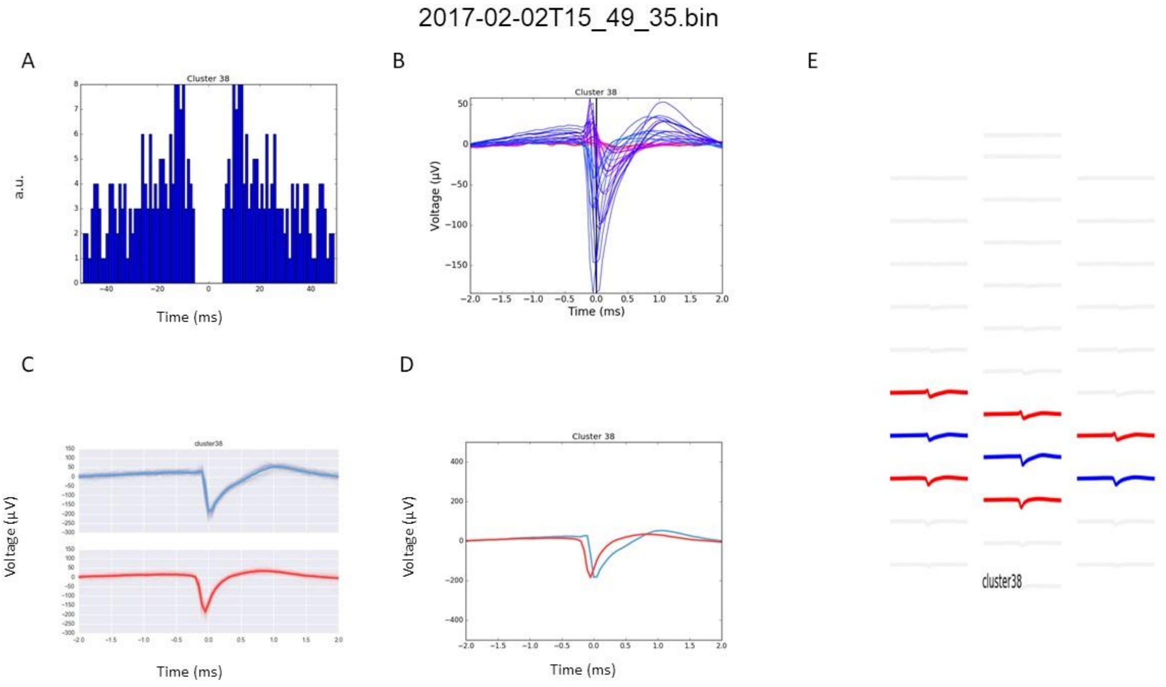
2017-02-02T15_49_35.bin
A
B
E
Cluster 38
a.u.
Time (ms)
Voltage (μV)
C
D
cluster38
Cluster 38
Time (ms)
Voltage (μV)
cluster38

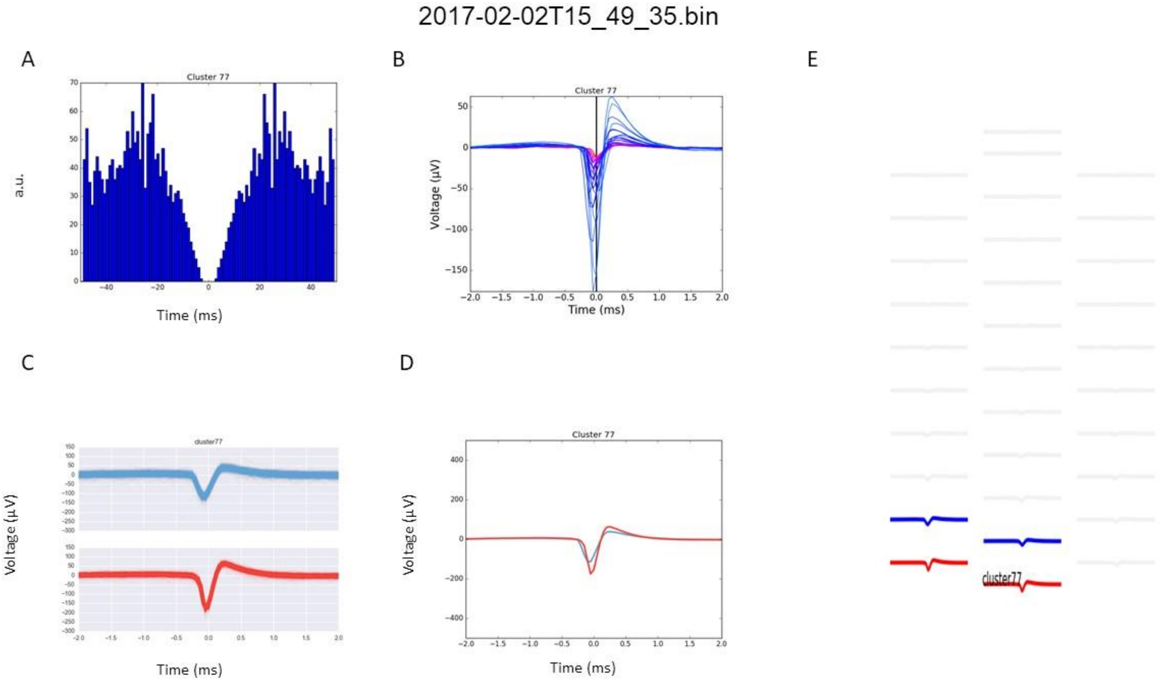
2017-02-02T15_49_35.bin
A
B
E
Cluster 77
a.u.
Time (ms)
Voltage (μV)
C
D
cluster77
Cluster 77
Time (ms)
Voltage (μV)
cluster77

2017-02-02T16_57_16.bin

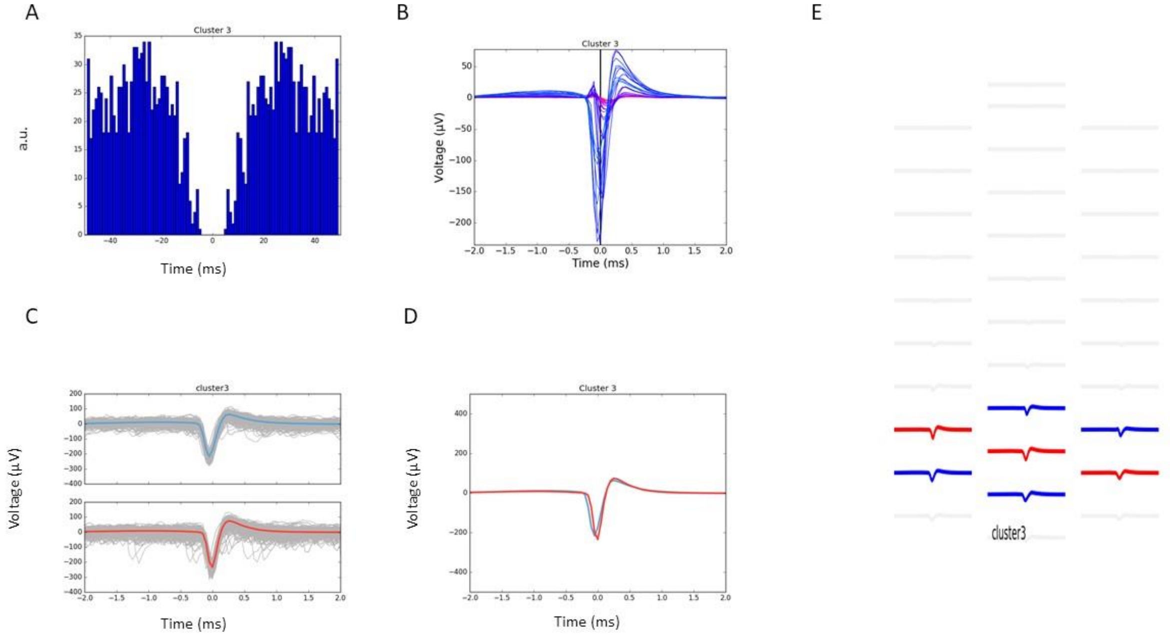

2017-02-02T16_57_16.bin

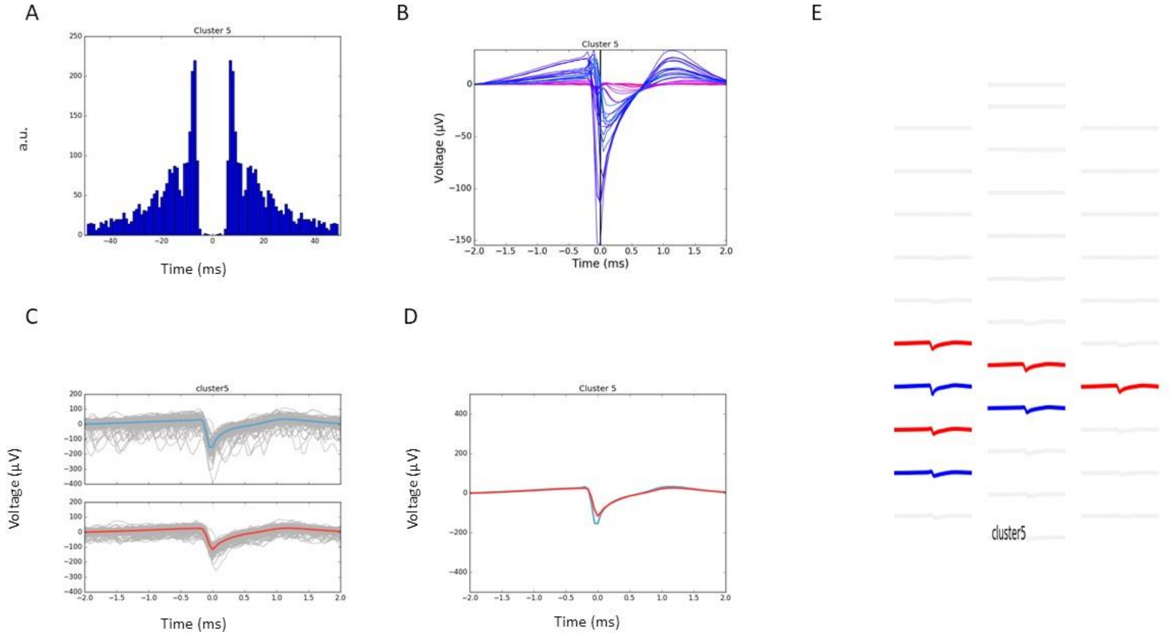

2017-02-02T16_57_16.bin

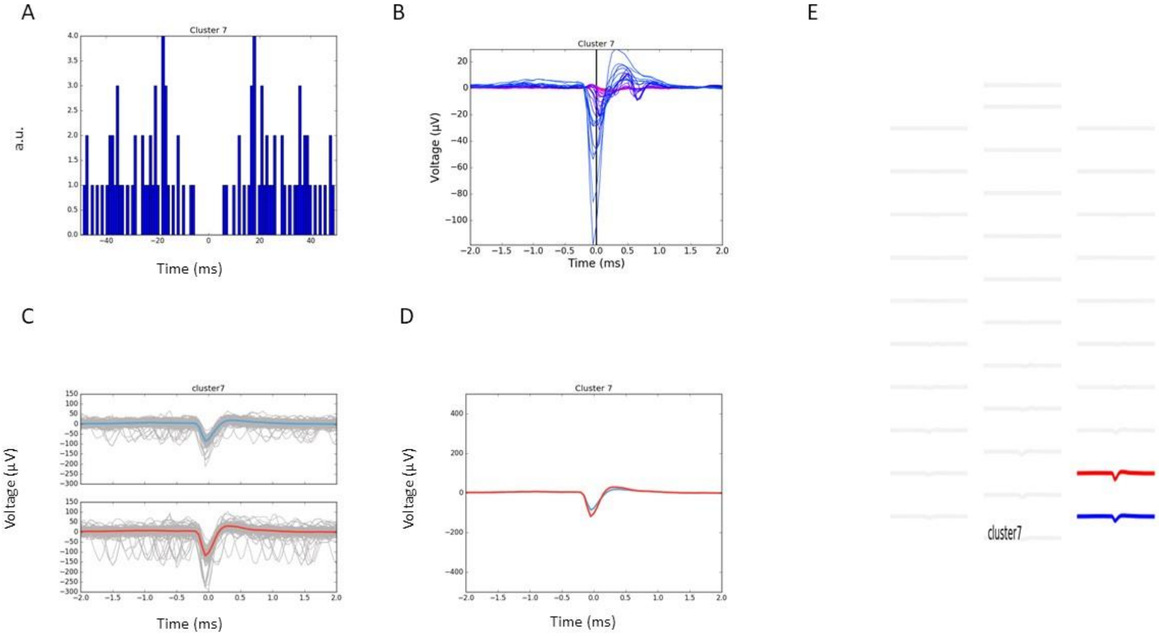

2017-02-02T16_57_16.bin

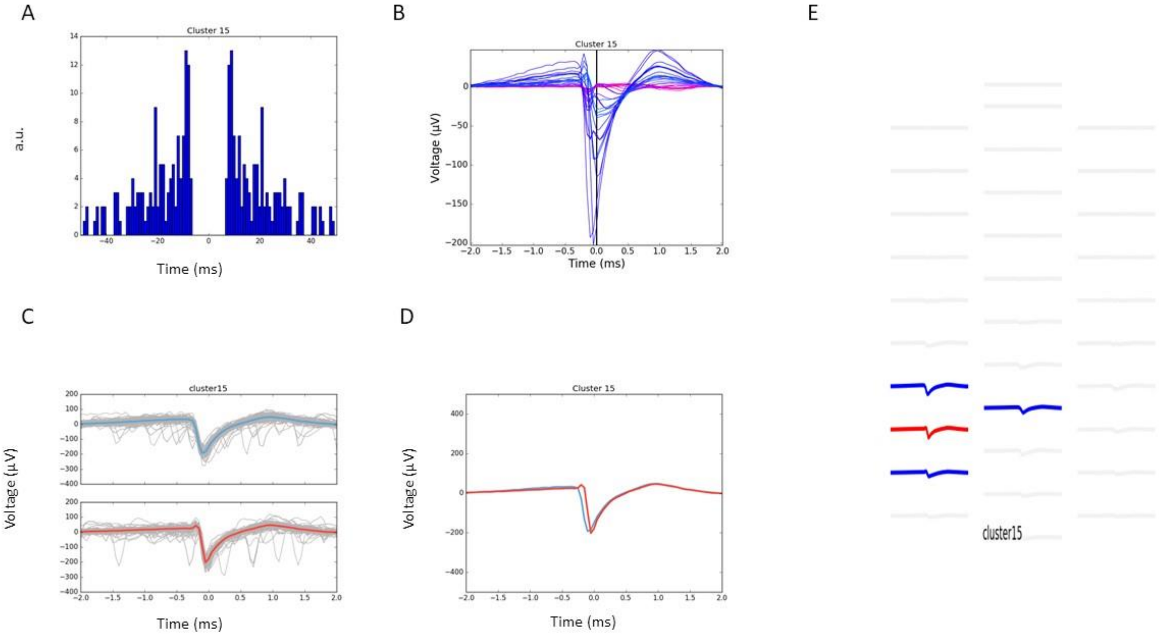

2017-02-02T16_57_16.bin

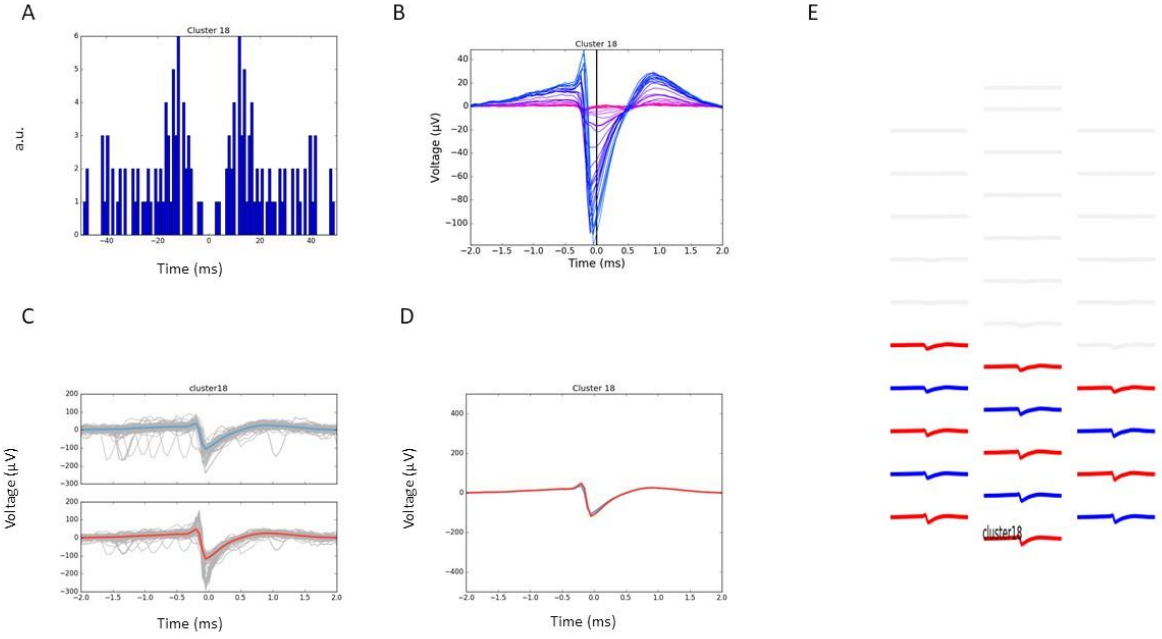

2017-02-02T16_57_16.bin

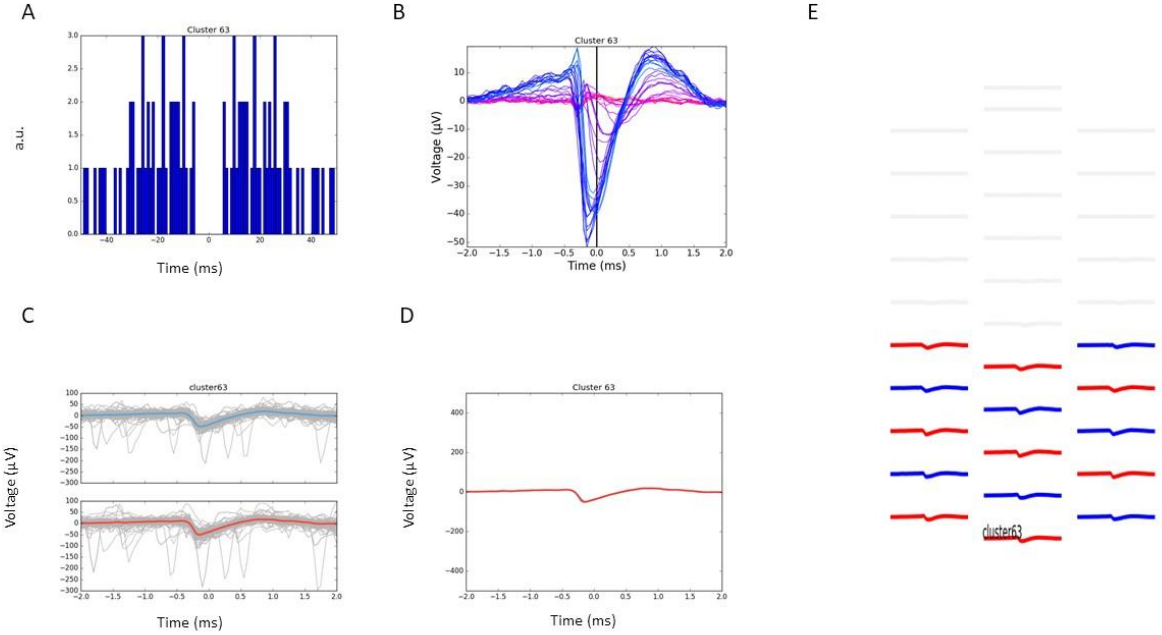

2017-02-02T17_18_46.bin

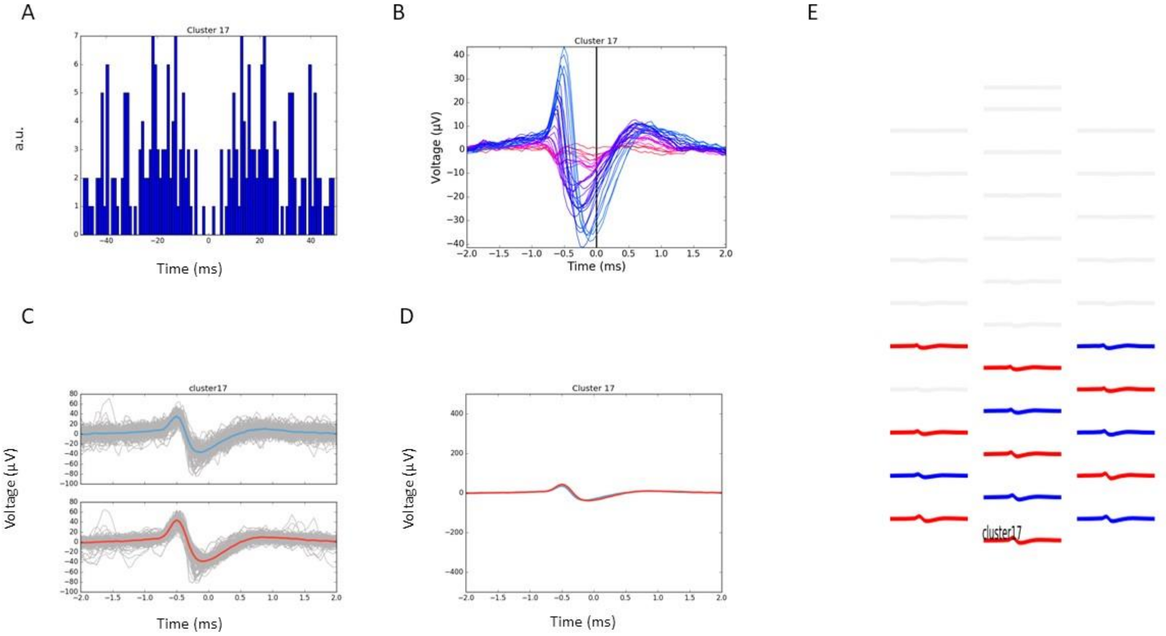

2017-02-02T17_18_46.bin

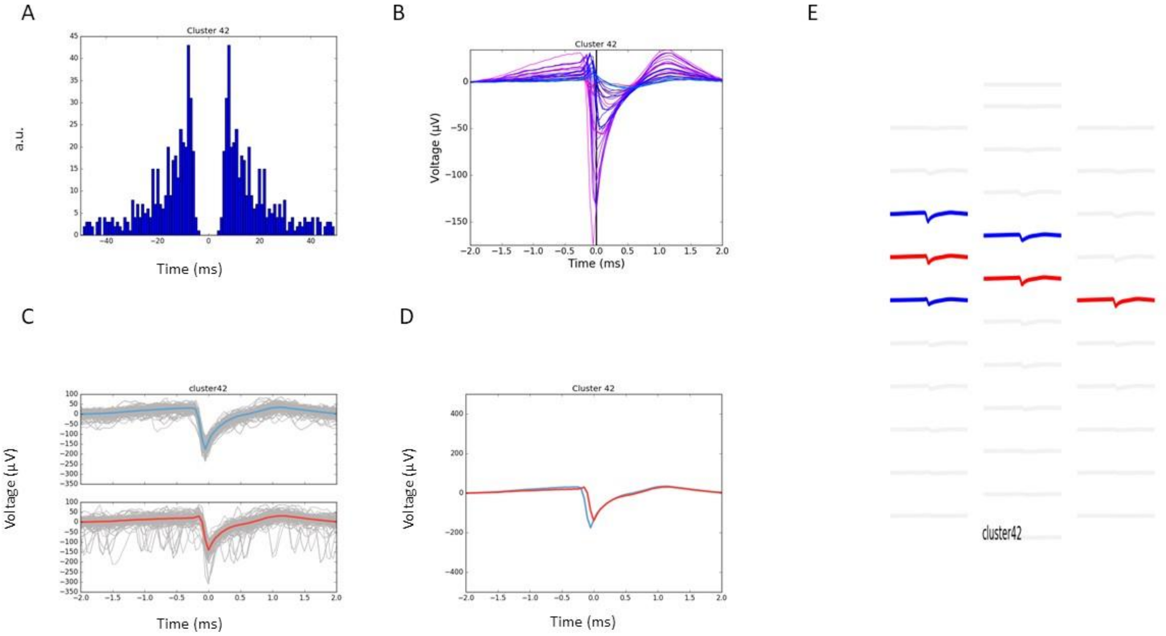

2017-02-02T17_18_46.bin

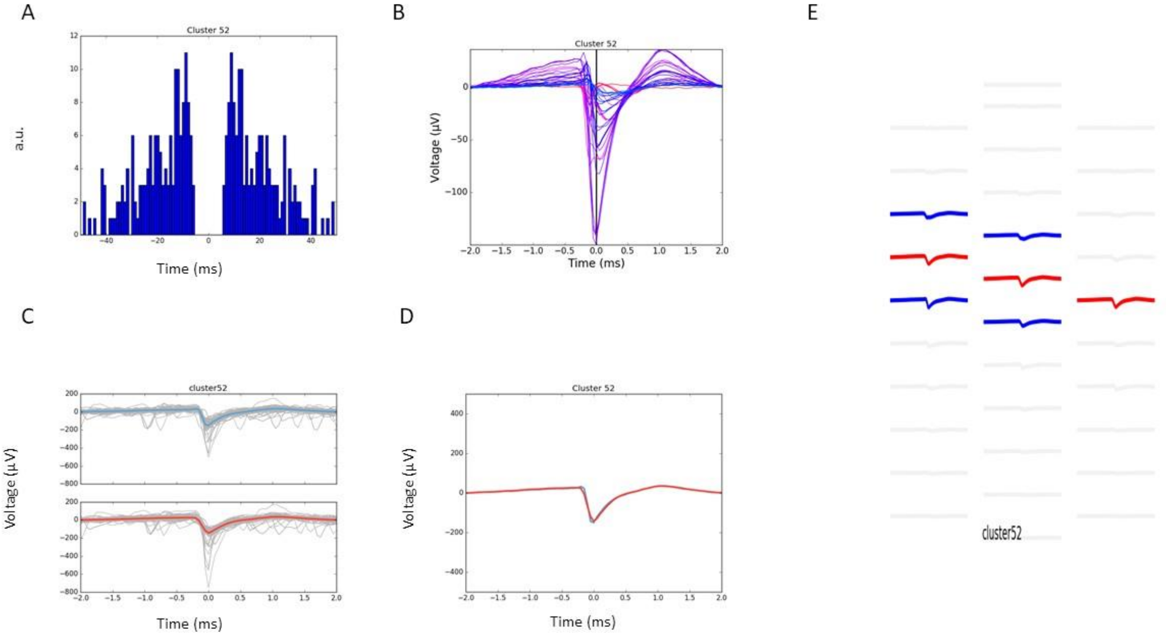

2017-02-02T17_18_46.bin

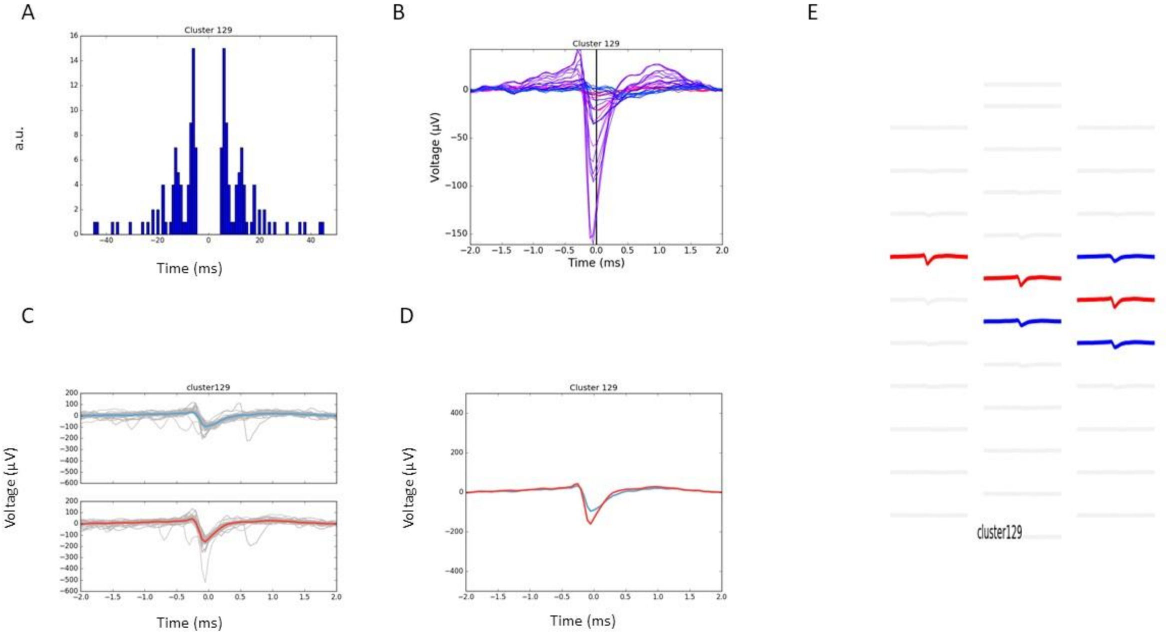

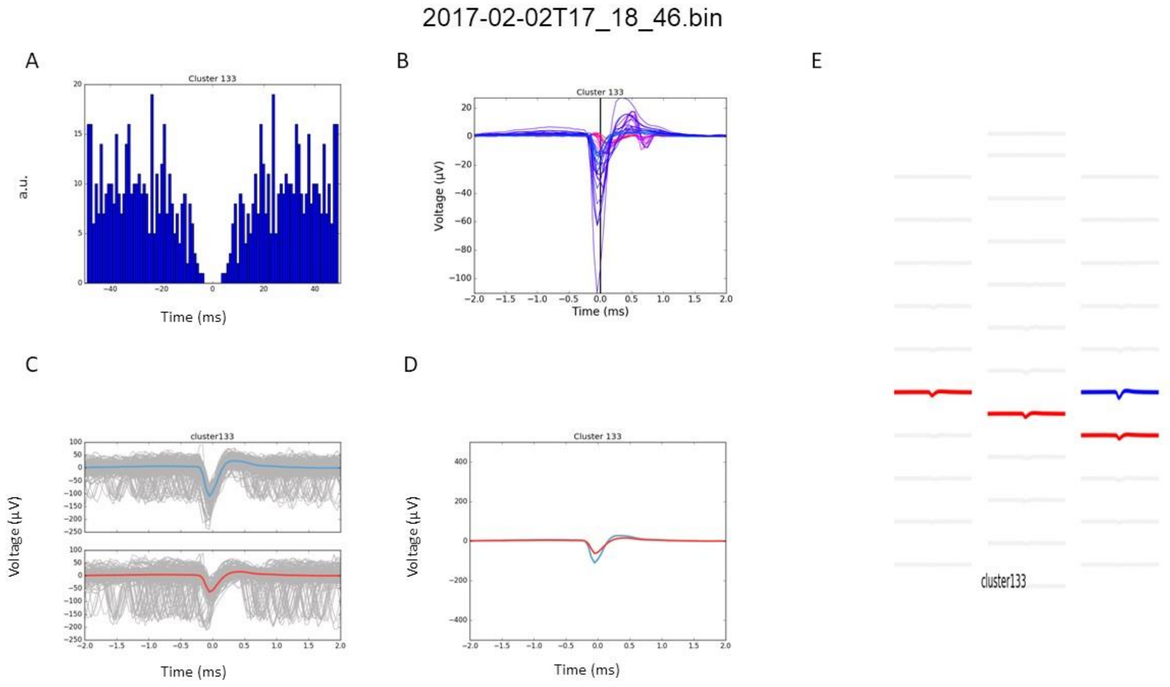

Supplementary Figure 3. Putative neurons (n=103) sorted from all the recordings. For each neuron (also called cluster), the recording is identified on the top. **(A)** Autocorrelogram showing low interspike interval violations; **(B)** Extracellular average time courses for each channel are overlaid; **(C)**-**(D)** The pristine and PEDOT coated electrodes with the highest average peak-to-peak amplitudes are represented as blue and red, respectively; **(E)** A schematic of the polytrode with red and blue colored waveforms denoting the electrodes where the peak-to-peak average amplitude is higher than half of the maximum peak-to-peak average amplitude for this neuron. When only one electrode is present in this representation, **(C)** and **(D)** are not shown. 'a.u.' arbitrary units.

Impedance spectroscopy

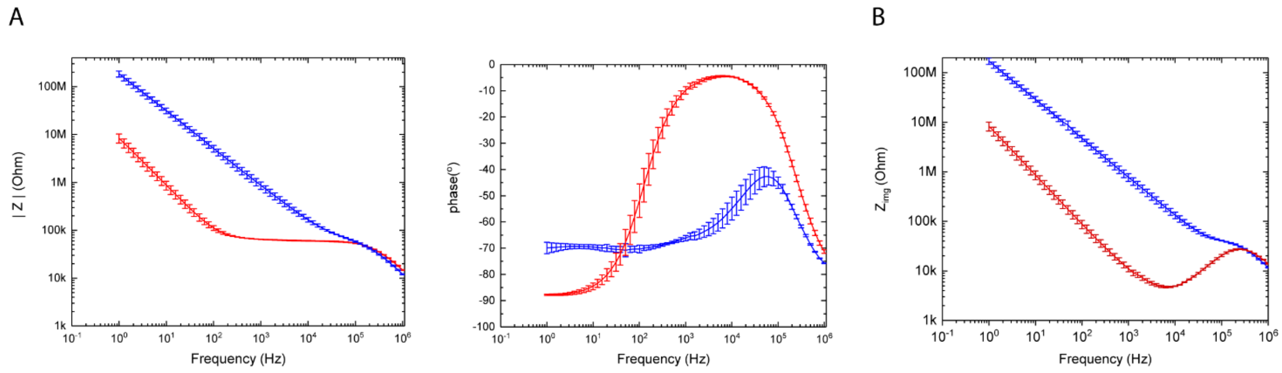

Supplementary Figure 4. Impedance spectroscopy of PEDOT-PSS (n = 3) and pristine (n = 3) electrodes, where red represent PEDOT coated electrodes and blue represent pristine electrodes. **(A)** Bode plot showing the impedance magnitude, |Z|, and phase angle; **(B)** The imaginary part of impedance.

Thermal noise calculation

Thermal noise is commonly believed to be one of the main contributors to the background noise in extracellular recordings [1]–[5]. The thermal noise is given by:

$$\sigma^{th} = \sqrt{4\,k\,T \int_{f1}^{f2} Z_{real}\, df}$$

Where k is the Boltzmann's constant (1.38 x 10^{-23} J/K), T is the temperature in degrees Kelvin, f_1 and f_2 are the lower and upper limits of the recording bandwidth in Hz, and Z_{real} is the real part of the impedance in the respective frequency bandwidth (f_1 to f_2). We assumed T = 293 K and we used OriginPro 2017 software to integrate the Z_{real} in the 200-8000 Hz frequency band. The σth for pristine microelectrodes was 5.0 μV and for coated microelectrodes was 2.8 μV.

Electrode impedance and attenuation of signal amplitude

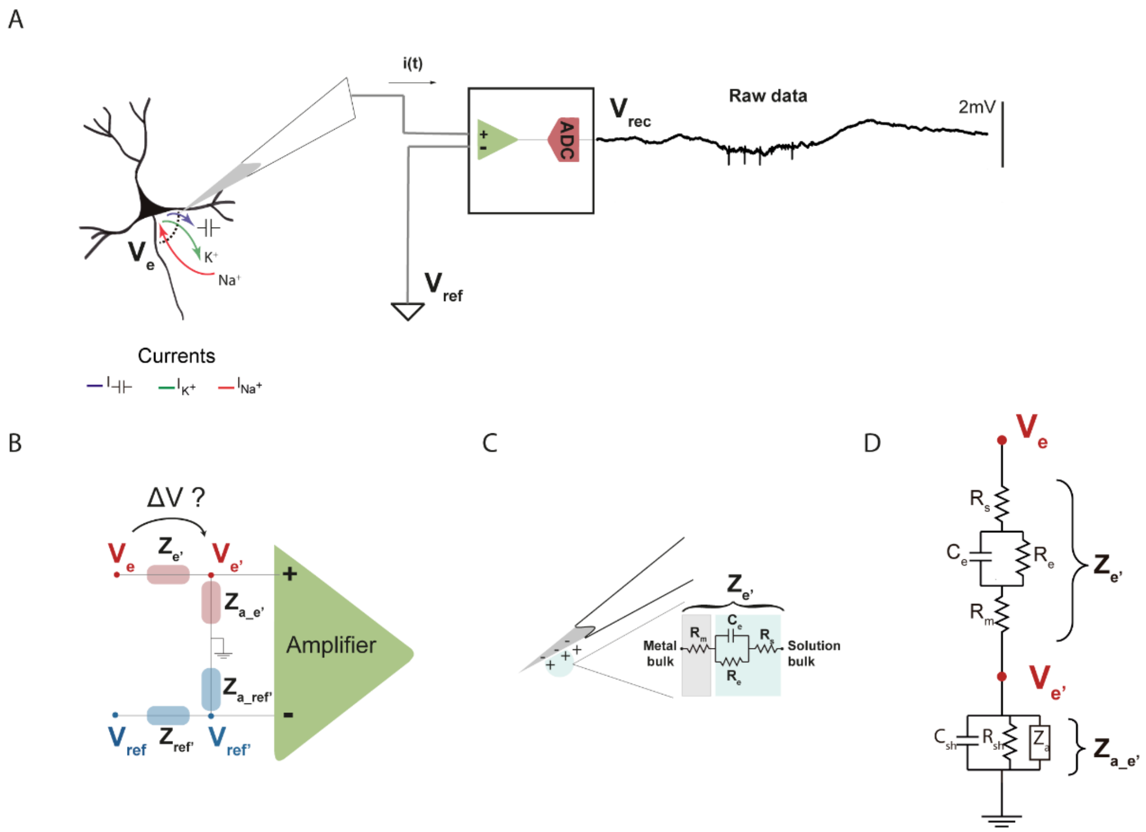

Supplementary Figure 5. Effect of electrode impedance on signal amplitude. **(A)** When a neuron is active, transient changes in its membrane cause currents (ionic and capacitive) to flow into and out of the cell. Each of these transmembrane currents superimpose in the extracellular medium, defining an electric potential (V_e) that surrounds the active neuron [10]. Therefore, to detect the presence of an active neuron in the extracellular space, the electric potential V_e relative to some distant reference must be measured. The potentials V_e and V_{ref} are the potentials impressed at the microelectrode and reference, respectively. In an ideal extracellular recording system, the recorded voltage V_{rec}, will be equal to $V_e – V_{ref}$, and it will be the potential difference that would exist if the tip had no net current flowing into it [11]. Nonetheless, the recorded voltage V_{rec} relies on a recording system. The recording system is composed of the microelectrode(s), reference electrode, and the hardware connected to them, including amplifiers, filters, and analog-to-digital converter (ADC); **(B)** Simplified circuit diagram [6] for understanding the voltage drops and current pathways that occur in a recording system. $Z_{e'}$ and $Z_{ref'}$ represent the effective electrode impedance for recording and reference electrodes, respectively. $Z_{a\_e'}$ represent the effective amplifier input impedance for the electrode and $Z_{a\_ref'}$ the effective amplifier input impedance for the reference. Due to currents that flow to ground through the series combination of the effective electrode impedance $Z_{e'}$ and the effective amplifier input impedance $Z_{a\_e'}$, the potential difference measured by the amplifier ($V_{e'}$ – $V_{ref'}$) may differ from the true potential difference ($V_e – V_{ref}$); **(C)** The electrical double layer at an electrode surface and equivalent circuit model of the interface of a metal microelectrode recording in the brain. When a metal is placed in a saline solution, two phenomena occur: water dipoles close to the metal surface become oriented, and assuming the metal surface is negatively charged, the solution

close to the metal surface become depleted of negative ions (anions), leaving behind a cloud of positive ions (cations). The resulting charge distribution - two narrow regions of equal and opposite charge - is known as the electrical double layer [12]. The effective electrode impedance ($Z_{e'}$) is the sum of the resistance of the solution (R_s), the resistance of the electrode metal (R_m), and the resistance (R_e) and capacitance (C_e) of the double-layer that forms on the metal electrode-extracellular interface; **(D)** Voltage divider: the effective electrode impedance $Z_{e'}$ is connected in series with the input amplifier impedance $Z_{a\_e'}$ which includes the actual input amplifier impedance and the shunting paths to ground outside the amplifier. The shunting routes to ground outside the amplifier are R_{sh} and C_{sh} [7]–[9]. This shunt capacitance arises mainly from the capacitance across the thin insulation isolating an electrode shaft and the surrounding electrolyte, as well as the cumulative capacitance along cables and connectors [11]. This route to ground, parallel to the amplifier, reduces the effective amplifier impedance, and being capacitive, this effect increases with signal frequency [9], [11], [13]. Therefore, the shunt capacitance should be small to create a large shunt impedance, especially when the electrodes have a large impedance [11], [13]. If $Z_{a\_e'}$ is not substantially larger than $Z_{e'}$, $V_{e'}$ will be less than V_e. Therefore, as long as the effective amplifier input impedance is much larger than the effective electrode impedance, the voltage drop in the electrode-extracellular interface is negligible and the potential difference at the amplifier inputs will reflect the actual difference in electric potential, V_e - V_{ref}.

Amplitude of action potentials

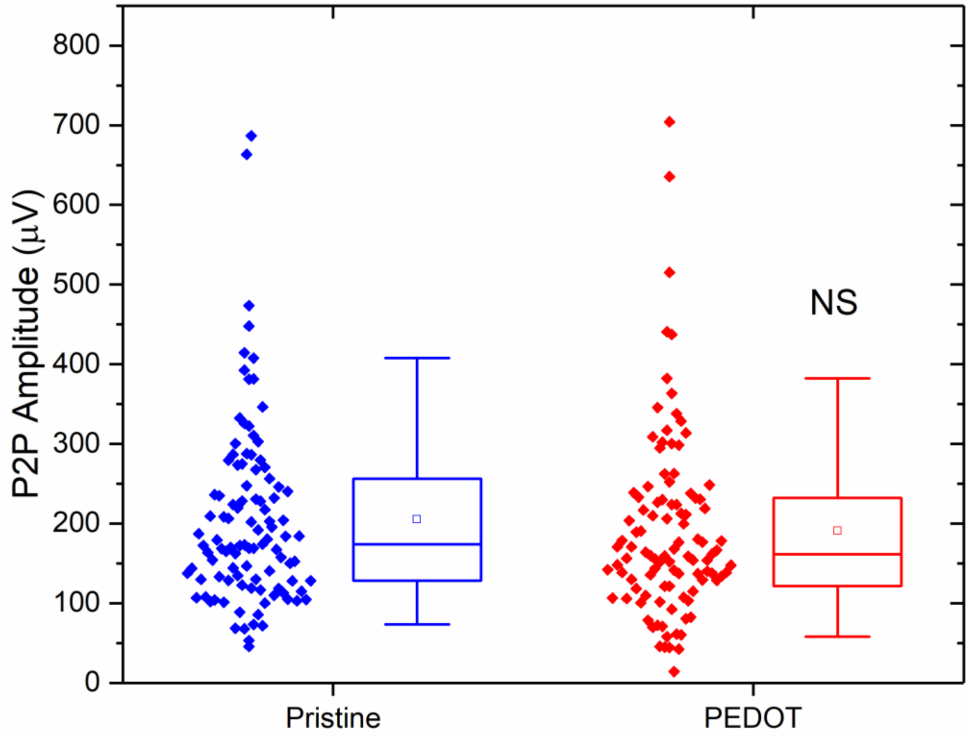

Supplementary Figure 6. Effect of electrode impedance on spikes amplitude. For each of the 103 putative neurons sorted from 11 recordings, the maximum average peak-to-peak (P2P) amplitudes from the pristine and PEDOT electrode groups were plotted. The P2P amplitude values are represented in scatterplots, and boxplots show the distribution of these values. In the boxplots, line: median, square: mean, box: 1st quartile–3rd quartile, and whiskers: 1.5 x interquartile range above and below the box. 'NS' not significant ($p > 0.05$) when compared with pristine electrodes.

References

[1] S. A. Desai, J. D. Rolston, L. Guo, and S. M. Potter, “Improving impedance of implantable microwire multi-electrode arrays by ultrasonic electroplating of durable platinum black.,” *Front. Neuroeng.*, vol. 3, no. May, p. 5, 2010.

[2] A. Hassibi, R. Navid, R. W. Dutton, and T. H. Lee, “Comprehensive study of noise processes in electrode electrolyte interfaces,” *J. Appl. Phys.*, vol. 96, no. 2, pp. 1074–1082, Jul. 2004.

[3] M. O. Heuschkel, M. Fejtl, M. Raggenbass, D. Bertrand, and P. Renaud, “A three-dimensional multi-electrode array for multi-site stimulation and recording in acute brain slices,” *J. Neurosci. Methods*, vol. 114, no. 2, pp. 135–148, 2002.

[4] J. B. Johnson, “Thermal Agitation of Electricity in Conductors,” *Phys. Rev.*, vol. 32, no. 1, pp. 97–109, Jul. 1928.

[5] H. Nyquist, “Thermal Agitation of Electric Charge in Conductors,” *Phys. Rev.*, vol. 32, no. 1, pp. 110–113, Jul. 1928.

[6] T. C. Ferree, P. Luu, G. S. Russell, and D. M. Tucker, “Scalp electrode impedance, infection risk, and EEG data quality,” *Clin. Neurophysiol.*, vol. 112, no. 3, pp. 536–544, Mar. 2001.

[7] G. Baranauskas, E. Maggiolini, E. Castagnola, A. Ansaldo, A. Mazzoni, G. N. Angotzi, A. Vato, D. Ricci, S. Panzeri, and L. Fadiga, “Carbon nanotube composite coating of neural microelectrodes preferentially improves the multiunit signal-to-noise ratio,” *J. Neural Eng.*, vol. 8, no. 6, p. 66013, Oct. 2011.

[8] S. F. Lempka and C. C. McIntyre, “Theoretical Analysis of the Local Field Potential in Deep Brain Stimulation Applications,” *PLoS One*, vol. 8, no. 3, p. e59839, Mar. 2013.

[9] M. J. Nelson, P. Pouget, E. A. Nilsen, C. D. Patten, and J. D. Schall, “Review of signal distortion through metal microelectrode recording circuits and filters,” *J. Neurosci. Methods*, vol. 169, no. 1, pp. 141–157, Mar. 2008.

[10] G. T. Einevoll, C. Kayser, N. K. Logothetis, and S. Panzeri, “Modelling and analysis of local field potentials for studying the function of cortical circuits.,” *Nat. Rev. Neurosci.*, vol. 14, no. 11, pp. 770–85, Oct. 2013.

[11] D. A. Robinson, “The electrical properties of metal microelectrodes,” *Proc. IEEE*, vol. 56, no. 6, pp. 1065–1071, 1968.

[12] R. Musa, “Design, fabrication and characterization of a neural probe for deep brain stimulation and recording.,” 2011.

[13] M. E. J. Obien, K. Deligkaris, T. Bullmann, D. J. Bakkum, and U. Frey, “Revealing neuronal function through microelectrode array recordings,” *Front. Neurosci.*, vol. 8, no. JAN, p. 423, Jan. 2015.
